# Diversity and distribution of the Huastec Mayan medicinal plants: hotspots for bioprospecting and conservation

**DOI:** 10.3897/BDJ.13.e170091

**Published:** 2025-12-08

**Authors:** Madeleyne Cupido, José Arturo De-Nova, Virginia Gabriela Cilia-López, Francisco Javier Pérez-Vázquez, Pablo Delgado-Sánchez

**Affiliations:** 1 Universidad Autónoma de San Luis Potosí, San Luis Potosí, Mexico Universidad Autónoma de San Luis Potosí San Luis Potosí Mexico

**Keywords:** biodiversity, ethnomedicine, Huasteca, risk category, traditional knowledge

## Abstract

The multi-ethnic biocultural region Huasteca in Mexico has a variety of practices, including the use of traditional plant-based medicine. Here, we analyse the diversity and distribution of the medicinal plants used by the Tenek culture, to identify hotspots for bioprospecting and conservation. We describe their diversity, growth forms, origin and endemism, classes of diseases, parts used and conservation risk status. A total of 468 plant species used for medicinal purposes were recorded, from 113 families and 350 genera. More than 50% of the species richness is concentrated in 10 families and 11 genera. A total of 418 species are native, 23 of them are endemic, while 50 are non-native. These species are obtained from one or more habitats, including 356 from the wild, 237 from home gardens, 111 from milpas, 20 from sugarcane plantations and 16 from disturbed habitats. The most common growth form is herbaceous and the most commonly used parts are leaves. Geographic hotspots with high species richness, disease classes diversity and high ethnomedicinal index values occur in Aquismón, San Antonio and Tantoyuca. Our results show that the biocultural region Huasteca is a reservoir of current benefits and future option values for bioprospecting and preserving of biocultural heritage. Further studies should employ phylogenetic approaches to elucidate evolutionary patterns and processes implied selection of medicinal plants by local people of medicinal plants. This will help identify lineages with phytochemicals, improve bioprospecting and ensure benefits for local people. As knowledge holders, they contribute to the conservation of medicinal plants.

## Introduction

**Figure 1.**
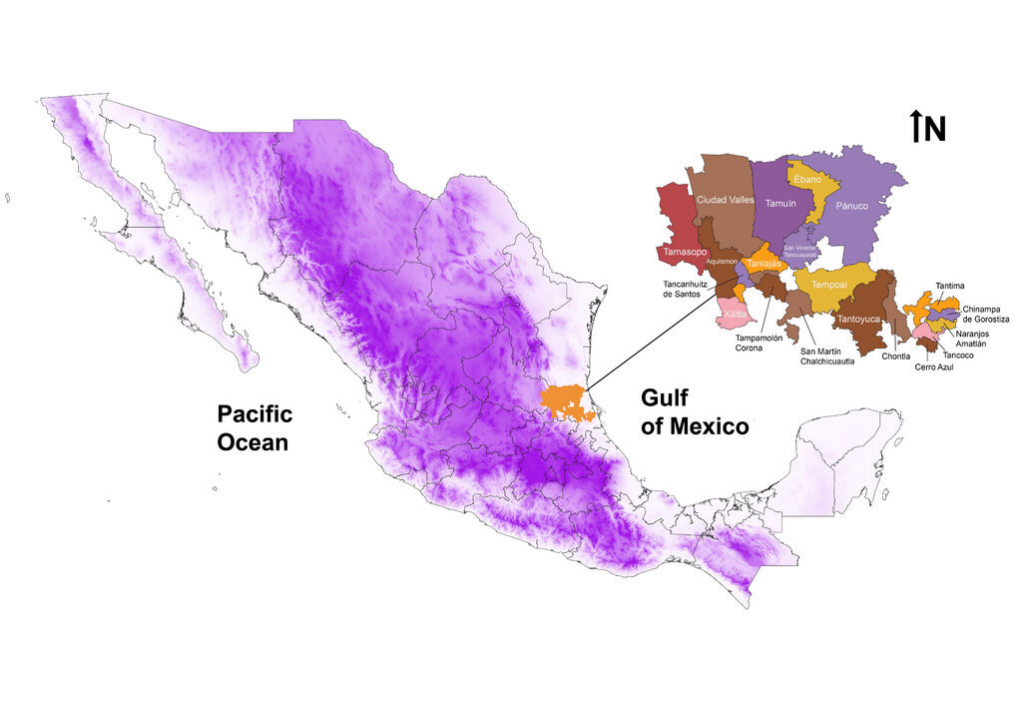
Tenek territory and their municipalities in States of San Luis Potosí and Veracruz, Mexico. The purple colour gradient illustrates the orography.

The use of plants has been an integral part of the traditional systems of medicine for centuries for humans around the world (Cox 1994, Roopashree et al. 2024). Plants have played an important role in traditional medicine in the world and, currently, ca. 23,842 plant species have been recorded as medicinal in the world (Pironon et al. 2024). Due to overexploitation, expansion of alien invasive species and climate change, medicinal plants are facing an increasing risk of habitat destruction and extinction (Huang et al. 2011, Huang et al. 2012, Pandey et al. 2018). Currently, identifying hotspots (critical areas for preservation) is a useful method for conservation efforts, because it helps select priority areas for conservation and improves the sustainable use of biodiversity (Myers et al. 2000). This identification of biodiversity hotspots is highly recommended in order to take into account multiple conservation indicators, such as the diversity of species found in a region, endemic species, threatened species and nationally protected species (Forest et al. 2007, Huang et al. 2012), as well as the diversity of human disease types found in a given region. However, despite being the second country in terms of medicinal plant use, Mexico has not carried out this type of study yet (Muñetón-Pérez 2009, Loraine and Mendoza-Espinoza 2010, Cruz-Pérez et al. 2021). Currently, traditional medicine in Mexico has a strong ancestral tradition in the use of medicinal plants. Between 3,000 and 4,500 species are used (Bye 1993, Argueta et al. 1994, CONABIO 2006, Muñetón-Pérez 2009) by more than 56 ethnic groups (Martin et al. 2011). Their ancestral presence and interactions with the environment and local communities have allowed the development of multiple forms of knowledge and the use of natural resources, especially plants, to address various health problems. The biocultural region Huasteca, located in north-eastern Mexico (Fig. 1), has been described as multi-ethnic and a biocultural region (Alcorn 1984), where different practices have been developed over time, such as traditional medicine, which is part of the local population's lives and used to treat different diseases and symptoms (Domínguez and Alcorn 1985). The most important ethnic group is the Tenek people, a descendant lineage of the ancient Maya with a rich biocultural heritage (Alcorn 1984, De-Nova et al. 2024). This heritage is the result of the territory's complex geographical and geological configuration of their territory, in the so-called Mexican transition zone between the Nearctic and Neotropical biogeographic regions. The landscape of their plant diversity is estimated to contain ca. 1,858 species of vascular plants, mainly with a tropical affinity, that inhabit the different types of seasonally dry tropical forests (De-Nova et al. 2024). It has recently been indicated from an evolutionary framework that the Huastec Mayan (Tenek) useful plants represent a vast source of current benefits and future option values (i.e. options to benefit from future uses of biodiversity) for human well-being (De-Nova et al. 2024).

Traditional medicine, based on plants, contributes significantly to the health of the population, especially in indigenous communities (WHO 2013). A high proportion of information on traditional medicine has been recorded in different parts of the world, but these data are often scattered in isolated studies on a particular species or lost, as this knowledge is passed down orally from generation to generation. Our objective was to analyse the diversity and distribution of the Huastec Mayan medicinal plants used by the Tenek in the biocultural region Huasteca to spatially identify ethnomedicinal hotspots for bioprospecting and conservation. We describe taxonomic diversity, growth forms, origin and endemism, classes of diseases, parts used and risk categories. We also discuss the relevance of these plants as a source of option values, particularly in the context of the search for pharmacological compounds.

## Material and methods

### Study area

The study area is the Tenek territory in the biocultural region Huasteca, located in north-eastern Mexico, mainly in the States of Veracruz, Hidalgo and San Luis Potosi, including high agrobiodiversity, multiple uses and option values of biodiversity for human well-being (De-Nova et al. 2024). The types of vegetation range from subtropical and tropical forests at lower altitudes (50-800 m) to cloud and pine-oak forests at higher altitudes (600-2,000 m). This region is located between the physiographic provinces of the coastal plain of the Gulf of Mexico and the Sierra Madre Oriental and corresponds to the hydrological region of the Panuco River (Fig. 1). The environments in the region provide important abiotic conditions that promote a high diversity of vegetation types, making it one of the priority inland regions of Mexico. Its flora includes 1,858 species (742 with some ethnobotanic use), mainly from tropical affinity that are elements of tropical and seasonally dry tropical forests, but also from temperate pine and oak forests and relicts of cloud forests (De-Nova et al. 2024). This study focuses on the Tenek people and their territory, who have inhabited the region and have managed and shaped the ecosystem for more than 3,000 years (Alcorn 1984). Over the last century, however, changes in land use caused by the commercial expansion of agriculture and livestock have significantly impacted the environment. In critical areas of the Huasteca, most of the original vegetation has disappeared and been replaced by areas of extensive agriculture and livestock (Reyes Hernández et al. 2006).

### Database

An updated database of plant species used in traditional medicine by the Tenek culture was compiled, based on the ethnobotanical atlas published by Alcorn (1984). This atlas recorded information and data on the various uses of plants in the region. We included information on the medicinal plants listed in this atlas, including additional data on each species. This information was recently published in the catalogue of medicinal plants of the biocultural region Huasteca (Cupido et al. 2023). Nomenclature was updated using the Taxonomic Name Resolution Service (TNRS), version 5.2 (Boyle et al. 2013) available in Plants of the World Online (POWO 2025). The database was developed with the following fields: family, species, common local names, habitat, origin (native or non-native), growth form, extinction risk category according to the Mexican NOM-059-SEMARNAT-2010 (SEMARNAT 2010), as assessed by authorities and the Red List of Threatened Species (IUCN 2023).

### Disease classification

The diseases and symptoms treated with Tenek medicinal plants correspond to a complex knowledge system that includes ancient, inherited practices, influenced by the West since the conquest. These were classified according to the ICD-11 (2024) of the World Health Organisation as follow: certain infectious or parasitic diseases (CIPD), diseases of the blood or blood-forming organs (DBBO), diseases of the circulatory system (DCS), diseases of the digestive system (DGS), diseases of the ear or mastoid process (DEMP), diseases of the visual system (DVS), diseases of the genitourinary system (DGUS), diseases of the musculoskeletal system or connective tissue (DMSC), diseases of the nervous system (DNS), diseases of the respiratory system (DRS), diseases of the skin (DS), endocrine, nutritional or metabolic diseases (ENMD), factors influencing health status or contact with health services (FIHS), injury, poisoning or certain other consequences of external causes (IPEC), mental, behavioural or neurodevelopmental disorders (MBND), neoplasms (NE), pregnancy, childbirth or the puerperium (PCP) and symptoms, signs or clinical findings not elsewhere classified (NEC). Additionally, we included culture-bound syndromes (CBS).

### Geographical distribution

A second database with geographical data was compiled with records of all the vascular plant species documented in the municipalities, historically occupied by the Tenek people (Tenek territory), extracted from the Herbarium SLPM database (SLPM 2025) and the GBIF platform (GBIF 2024), in order to identify hotspots that represent critical geographical areas for preserving biocultural heritage (Cámara-Leret and Bascompte 2021). The GBIF data include specimens from 40 different herbaria in Mexico and around the world, as well as direct observations curated by experts. Particular attention was paid to our institutional herbarium, SLPM, since it contains the largest collection of botanical vouchers from the region. Additionally, a copy of all specimens collected Alcorn (1984) is deposited there. An extraction was made for only the medicinal species included in the first database, including geographic coordinates and voucher information. No exclusion criteria were applied, except for specimens with gaps in relevant distribution or taxonomic information, as well as geographical inconsistencies. The years of the collection range from 1930 to 2024; however, 87% of the collection is from 1970 to 2024. Then, we assigned the classes of diseases of each species indicated in our first database (Suppl. material 1) and these were assigned to each geographical record. We used QGIS v.2.16.3 (QGIS 2025) to visualise the geographical distribution of medicinal plant species richness (SR) and number of categories covered in grid cells of 0.045 decimal degrees (ca. 5 km^2^). To spatially identify ethnomedicinal hotspots highlighting the clustering of medicinal plants attending a wide number of classes of diseases, we calculated an ethnomedicinal index (EI) as:

\begin{varwidth}{50in}\begin{equation*}
            EI = \Sigma_{i = 1} ^{n} \frac{n_{i}}{N}
        \end{equation*}\end{varwidth}

where *n* is the number of classes of diseases treated for the *i*-th species in a grid cell and *N* is the total number of classes of diseases treated for all species in the region (here 19 classes).

## Results

### Taxonomic richness and diversity

**Figure 2.**
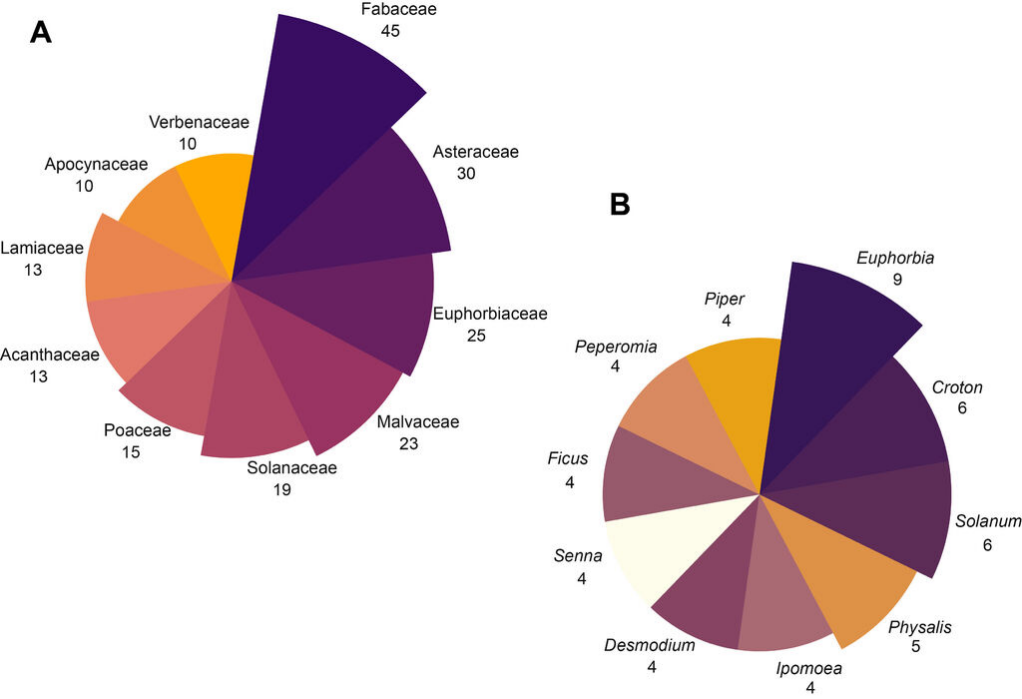
Highest richness of medicinal plant species in the Tenek territory by families (A) and genera (B).

**Figure 3.**
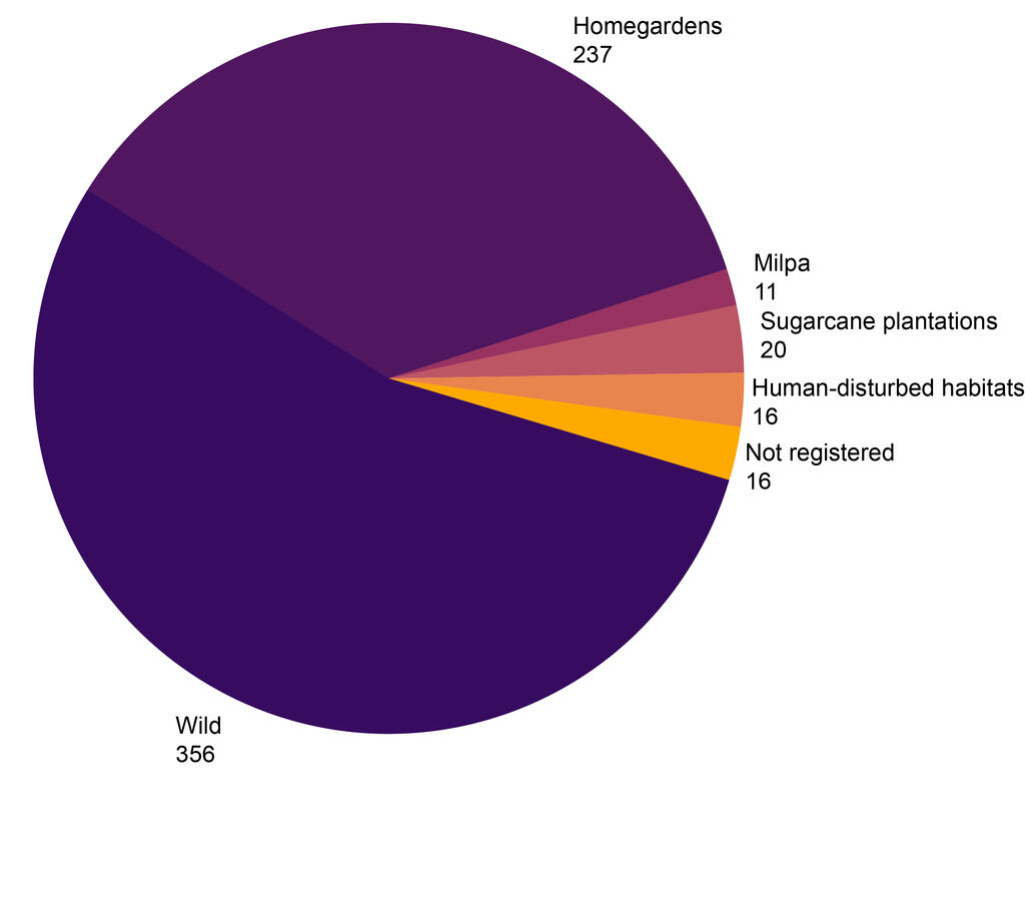
Number of medicinal plants species recorded by habitat type in the Tenek territory.

**Figure 4.**
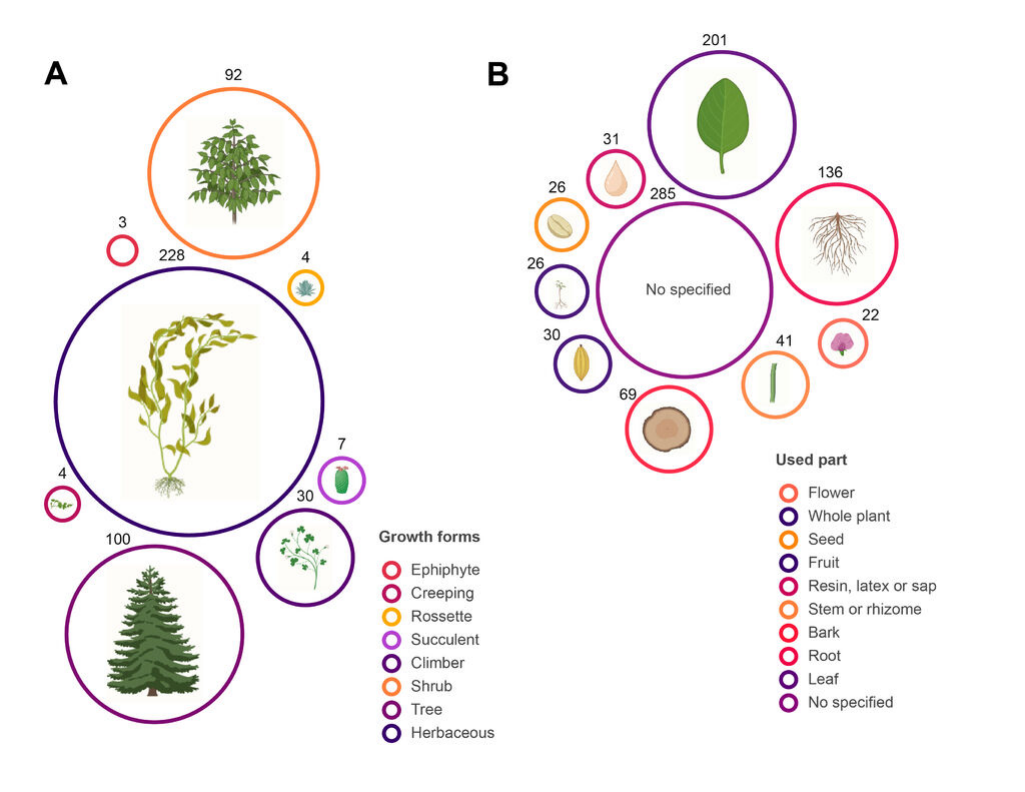
Species richness of medicinal plants in the Tenek territory growth forms (A) and used parts (B).

We recorded a total of 468 plant species used in the Huastec Mayan traditional medicine in the Tenek territory, belonging to 113 families and 350 genera (Suppl. material 1 and Suppl. material 2). The families with the highest number of useful species (Fig. 2A) are Fabaceae (45 species), Asteraceae (30), Euphorbiaceae (25), Malvaceae (23), Solanaceae (19), Poaceae (15), Acanthaceae (13), Lamiaceae (13), Apocynaceae (10) and Verbenaceae (10), representing 43.37% of the total richness. The remaining 102 families include fewer than 10 species. The genera with the highest number of species (Fig. 2B) are *Euphorbia* L. (9), *Croton* L. (6) and *Solanum* L. (5). A total of 418 species are native (23 endemic) and 50 are non-native (Suppl. material 1 and Suppl. material 2). The most common habitats where medicinal plants are collected and obtained (Fig. 3) are in the wild (356 species), in home gardens (237) and in milpa (111). The most used growth forms (Fig. 4A) are herbaceous (228 species), trees (100) and shrubs (92). Growth forms such as rosette, creeping and epiphyte have less medicinal use. The most used parts (Fig. 4B) are leaves (201 species), followed by roots (136) and bark (69). A total of 141 species are considered in the IUCN Red List: 128 Least Concern, five Data Deficient, four Endangered, two Vulnerable, one Critically Endangered and one Near Threatened and three in the NOM-059-SEMARNAT-2010: two Threatened and one Subject to Special Protection (Suppl. material 1).

### Disease classification

Table 1 shows the number of diseases and symptoms, as well as the number of species used for mitigation and treatment. The five disease classes with the highest values are: 1) DS, with 65 different diseases and symptoms treated by 177 species; 2) NEC with 56 different diseases and symptoms and treated by 270 species; 3) DGS with 44 different diseases and symptoms treated by 161 species; 4) CBS with 40 different diseases and symptoms treated by 137 species; 5) CIPD with 30 different diseases and symptoms and 135 treated by species. A total of 259 species are used to treat more than three disease classes, 88 treat only two and 121 treat only one.

### Geographical distribution

**Figure 5.**
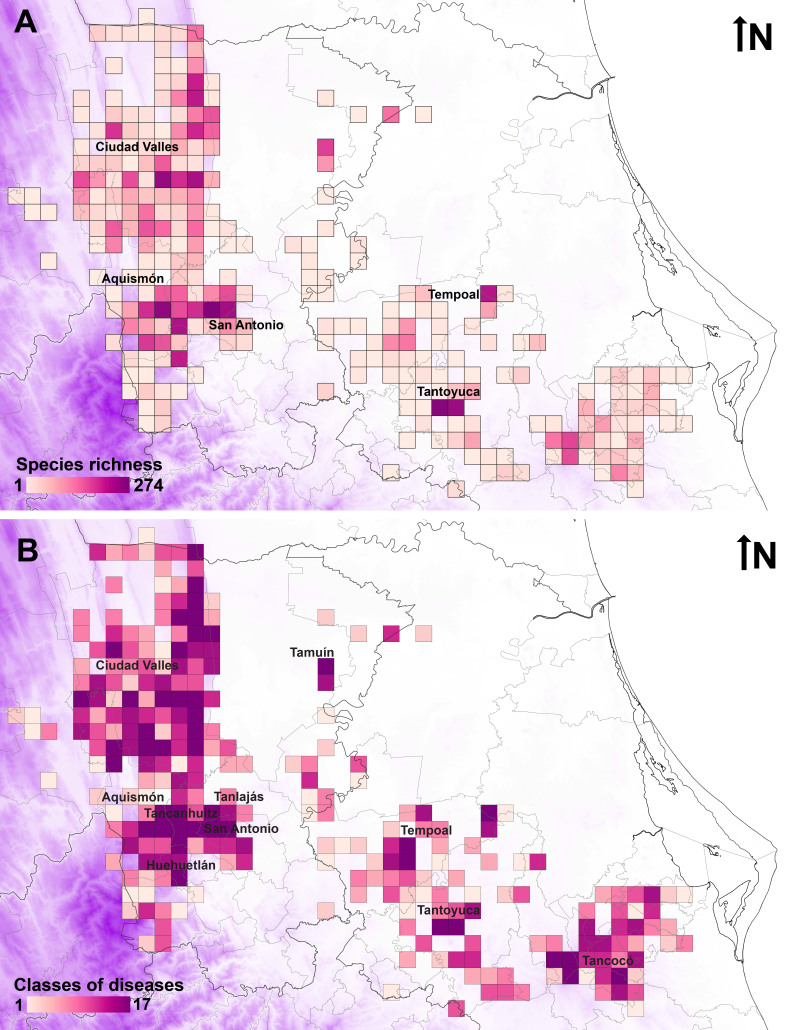
Medicinal plants in the Tenek territory by species richness (A) and classes of diseases (B).

**Figure 6.**
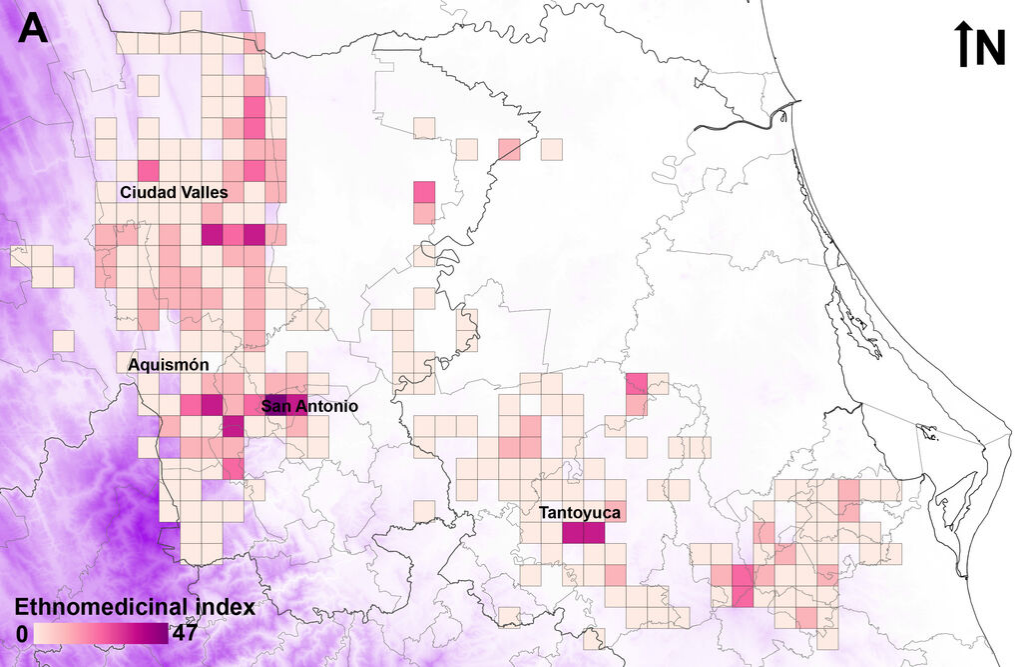
A) Ethnomedicinal index (EI) in the Tenek territory.

The geographical database for medicinal plants in the Tenek territory includes 5,341 records for 432 species in 275 grid cells (Suppl. material 3). Species richness is concentrated mainly in five municipalities (Fig. 5A): San Antonio (304 species, with two grid cells including 92 and 274 species), Ciudad Valles (261 species, with four grid cells including 50, 58, 79 and 100 species), Aquismón (221 species, with one grid cell including 106 species) in San Luis Potosí and Tantoyuca (195 species, with two grid cells that include 93 and 108 species) and Tempoal (114 species with a grid cell with 59 species) in Veracruz. The municipalities with more classes of diseases covered by plants (Fig. 5B) are Aquismón (19 classes, with eight grid cells with more than 14 classes), Ciudad Valles (19 classes, with 28 grid cells with more than 14 classes), Huehuetlán (19 classes, with three grid cells with more than 14 classes), San Antonio (19 classes, with two grid cells with more than 14 classes), Tamuín (19 classes, with one grid cell with more than 14 classes), Tancanhuitz (19 classes, with three grid cells with more than 14 classes) and Tanlajás (19 classes, with one grid cell with more than 14 classes) in San Luis Potosí and Tancocó (19 classes, with one grid cell with more than 14 classes), Tantoyuca (19 classes, with two grid cells with more than 14 classes) and Tempoal (19 classes, with four grid cells with more than 14 classes) in Veracruz. Four ethnomedicinal hotspots (Fig. 6) are located in municipalities San Antonio (two grid cells; ethnomedicinal index of 46.7 and 15.3) Tantoyuca (two grid cells; ethnomedicinal index of 21.8 and 15.9), Ciudad Valles (two grid cells; ethnomedicinal index of 17.4 and 15.3) and Aquismón (one grid cell; ethnomedicinal index of 17.5).

## Discussion

### Diversity of the Huastec Mayan medicinal plants

Documenting the diversity of medicinal plant species used in Tenek healthcare practices highlights the extensive use and effectiveness of traditional knowledge, as well as its significance to cultural heritage. According to Domínguez and Alcorn (1985) and Cupido et al. (2023), at least 550 plant species are identified as medicinal for the Tenek culture and, here, we compiled valuable information on the use practices for 468 of these species, which represent a critical source of bioactive phytochemical compounds for bioprospecting. Our findings aligns with the results of other studies that also identified the families Fabaceae, Asteraceae, Euphorbiaceae, Malvaceae, Solanaceae, Poaceae, Acanthaceae and Lamiaceae as having the most diverse medicinal uses (Bessada et al. 2015, Cilia-López et al. 2021, Islam et al. 2019, Rolnik and Olas 2021). This also agrees with the richest families used in traditional Mexican medicine, Asteraceae, Lamiaceae and Solanaceae (Bye et al. 1995). This selection of families is related to active compounds with therapeutic effects, as mentioned by Caballero et al. (2004). It suggests that phytochemicals with potential for bioprospecting are not phylogenetically randomly distributed.

The most commonly used plant genera in the Huastec Mayan traditional medicine are also relevant in other parts of the world for health and some of them have been the basis of commercial drugs. The genus *Euphorbia* includes ca. 1,600 species and at least 158 are used in traditional medicine in the world (Ernst et al. 2015). Plants in the genus *Euphorbia* have been highlighted for their chemical diversity with constituents with biological activities, such as anti-inflammatory, antileishmanial, antimicrobial, antioxidant, antitumoral, cytotoxic, immunomodulatory and neuroprotective (Chamkhi et al. 2022). Some of them have inspired commercial drugs such as Picato®, which contains Ingenol Mebutate found in the latex of the *Euphorbia peplus* L., a species used in Australian traditional medicine as a treatment for diseases of the skin, such as skin cancer and actinic keratosis (Fidler and Goldberg 2014, Ernst et al. 2015). The species *Euphorbia heterophylla* L., used for the classes culture-bound syndromes, diseases of the circulatory system, digestive system, respiratory system, pregnancy, childbirth or the puerperium and symptoms, signs or clinical findings, not elsewhere classified, has been studied previously, showing bioactivity and wound-healing potential (Omale and Friday 2010). The genus *Croton*, another Euphorbiaceae, includes ca. 1,300 species distributed throughout the world and several of them have traditional uses and pharmacological effects to treat diabetes, malaria, sexually transmitted diseases, cancer, inflammation, fever, digestive problems and fungal infections (Moremi et al. 2021). Although *Croton cortesianus* Kunth is used for the Tenek in certain infectious or parasitic diseases, diseases of the blood or blood-forming organs, diseases of the visual system, digestive system, genitourinary system, skin, injury, poisoning or certain other consequences of external causes and symptoms, signs or clinical findings not elsewhere classified, no research on its phytochemical, bioactivity or pharmacological properties has been conducted. The genus *Solanum* includes 2,500 to 3,000 species distributed worldwide in tropical and subtropical regions and several of them have been reported with medicinal traditional uses and pharmacological effects (Chidambaram et al. 2022). This genus has received much interest since biological activities such as anticancer, hepatoprotective, antimalarial, anthelmintic and others have been reported (Elizalde-Romero et al. 2021). Some species like *Solanum diphyllum* L., which is used in culture-bound syndromes, diseases of the ear or mastoid process, respiratory system, skin and symptoms, signs or clinical findings not elsewhere classified, have been studied previously showing bioactivity anticarcinogenic in the breast and colon (Hamada et al. 2010). The genus *Desmodium* contains about 350 species, mainly distributed in tropical and subtropical regions of the world and their biological activities, such as cardiovascular, cerebrovascular and immune system regulation, as well as anti-inflammatory, cytotoxic, antiparasitic, antioxidant and antiradical, antidiabetic, antipyretic and antibacterial activities, have been reported (Kurian and Paddikkala 2010, Muanda et al. 2011, Zhu et al. 2011). Medicinal uses of *Desmodium incanum* DC., used in diseases of the skin, injury, poisoning or certain other consequences of external causes and symptoms, signs or clinical findings not elsewhere classified, have been reported for promoting appetite, sleep, stamina, pain relief and possible treatment of hyperglycaemia and, although its use is not widespread, it has been shown that the plant is used as a natural defence against pests and bacteria (Beroni et al. 2023).

### Main diseases and plant used parts

Some of the main classes of diseases treated by the Huastec Mayan traditional medicine, such as diseases of the skin, are common health problems that affect people of all ages, from newborns to the elderly. Plant-based treatments for this class have been used for centuries, inspiring commercial drugs, such as Histoplastin Red® and Contractubex Gel® (Tsioutsiou et al. 2022). Several species and uses for diseases of the skin have also been recorded in the Tenek territory, for example, *Kalanchoe pinnata* (Lam.) Pers. is used to treat skin lumps, tumours, hard inflammations, sores, boils, ‘huuts'’ (boils, bad sores) and erysipelas. Another main class is diseases of the digestive system, one of the most common types of human disease treated with medicinal plants worldwide (Tangjitman et al. 2015). In the Tenek territory, a variety of species and uses have also been recorded to treat diseases of the digestive system, which are treated by medicinal plants. For example, *Ocimum campechianum* Mill. is used to treat stomach pain, stomach problems, vomiting diarrhoea and abdominal distension. Recurrent diseases, depending on the conditions and climate of the region where the people live, could drive the direction of the selection of medicinal plant species and their traditional practices of use through generations. The Tenek people live in a hot and humid tropical environment that could facilitate certain health problems. Previously, Domínguez and Alcorn (1985), have indicated several recurrent diseases, highlighting malnutrition (especially in children), chronic parasitic problems, allergies, asthma, tuberculosis, fungal infections, mycetoma, back problems, arthritis, ear infections, dysentery, diarrhoea, alcoholism and traumatic injuries. A particular disease class is IPEC, which has 19 different symptoms, including snakebites, a serious cause of accidental poisoning in San Luis Potosí (Tay et al. 2002, García-Willis et al. 2009). In the biocultural region Huasteca, the local people mention species of the Bothrops genus (lanceheads locally known as nauyaca) and the Elapidae family (coral snake) as medically important snakes. A total of 21 plant species are used to treat their bites. Some of these species are also used in other parts of the world for the same purpose. Examples include *Acacia cornigera* (L.) Willd., *Cleoserrata serrata* (Jacq.) Iltis, *Mikania cordifolia* (L.f.) Willd., *Nicotiana tabacum* L., *Solanum torvum* Sw., *Spondias mombin* L., *Rivina humilis* L. (see Coe and Anderson (2005), Hernandez et al. (2007), Nelson et al. (2007), Samy et al. (2008), Sanz-Biset et al. (2009), Caballero-George and Gupta (2011), Vásquez et al. (2013), Hasan et al. (2015), Kadir et al. (2015)), which supports the idea of ethnobotanical convergence previously mentioned by Garnatje et al. (2017)

As in other parts of the world, the traditional knowledge of the Huastec Mayan medicinal plants is linked to their biological, chemical and physical diversity, as well as to the biocultural memory of the people (Alcorn 1984, Toledo and Barrera-Bassols 2008). Ethnopharmacological studies in other parts of Mexico (e.g. Chiapas, Oaxaca, Veracruz and Yucatan) indicate that aromatic organoleptic properties are decisive for medicinal purposes (Leonti 2011). The morphological traits that Tenek people consider in the selection of medicinal plant species are diverse and the organoleptic properties of each species are also considered; however, they use mainly leaves from herbs to treat different diseases, which is the preferred used part recorded in other ethnobotanical studies. For example, for the selection of the ‘epazote’ (*Dysphania ambrosioides* (L.) Mosyakin & Clemants), the colour, size and shape of the leaf, in addition to the organoleptic characteristics, are criteria used by the mestizo inhabitants of Mazateco descent of Santa María Tecomavaca, Oaxaca, to differentiate its use as medicinal from various types of condiments (Blanckaert et al. 2012).

### Implications for plant conservation

The biocultural region Huasteca has experienced a significant reduction and fragmentation of tropical montane and semi-deciduous forest, caused by agricultural expansion (citrus, coffee), illegal logging and livestock farming, mainly since 1970 (Barthas 1996, Leija-Loredo et al. 2011, De-Nova et al. 2025). Although some areas where medicinal plant species occur have been transformed or disturbed, most of these species and their specimens have been preserved since then in the biocultural region Huasteca, highlighting its importance as conservation hotspots. Additionally, the habitats, from which locals retrieve medicinal plants (wild and home gardens), demonstrate a prevalent sense of preserving biodiversity values, such as medicinal resources and possible future options.

In Mexico, the official norm NOM-059-SEMARNAT-2010 (SEMARNAT 2010) lists the species at risk, based on the identification of those that are endemic, endangered, socially, economically, culturally or scientifically important, representative, unique or linked to evolutionary or other biologically significant processes in the country. Tenek medicinal plants are mainly extracted from wild habitats, where 144 species are registered in some risk category. Some of them are relevant for the high number of diseases and symptoms they treat. For example, *Campyloneurum phyllitidis* (L.) C.Presl, listed as endangered by the NOM-059-SEMARNAT-2010, is used for DCS, such as heart pain, DEMP, such as ringing in ears and NEC, such as dizziness. *Ateleia gummifera* (DC.) D.Dietr., listed as "endangered” in the IUCN list, is used for DS and the treatment of foot fungus. *Cedrela odorata* L., considered vulnerable by the IUCN and subject to special protection by the NOM-059-SEMARNAT-2010, is reported to treat various diseases such as anaemia, pain, cold extremities, dizziness, sleep, oedema, cough, malaria, wounds and cultural diseases, such as ‘hik'elomtalaab’ (spirit loss due to fright; symptoms include lack of appetite, gastrointestinal problems, pale complexion and, occasionally, fever), ‘haluk'laab’ (a term applied to those who are very sick and fail to respond to usual remedies; caused by unborn child seeking to take the ill person’s spirit in order to be born) and ‘tsamneklith’ (illness effected by a dead soul directly, patient being weak and dreaming of dead people) (Alcorn 1984). The wild cotton, *Gossypium hirsutum* L., a vulnerable species according to the IUCN, is traditionally used for its antimicrobial properties and has significant antifungal activity (De Lima et al. 2022) and it is reported to treat various diseases and symptoms for the Tenek people, such as chest pain, stomach pain, diarrhoea, green diarrhoea, waist pain, labour and preterm labour, urinary problems, anti-asthmatic and dysentery (Alcorn 1984). *Exothea copalillo* (Schltdl.) Radlk. is endangered according to the IUCN and is used by the Tenek to treat epilepsy, ‘ts'ebtsinal’ (loss of consciousness) and sickness (Alcorn 1984). *Esenbeckia berlandieri* Baill. is considered endangered by the IUCN and the Tenek use it for headache and vomiting.

Previously, it has been indicated that it is relevant to continue studying local communities in the biocultural region Huasteca to elucidate the selection processes on plant traits that allow the identification of benefits from option values of nature present in their territory (De-Nova et al. 2024). Understanding local knowledge of nature and the traditional use practices could allow the development of appropriate management strategies that are based on both scientific and local knowledge (Ijatuyi et al. 2025). Systematised information on medicinal plants represents a nature-based solution to address emerging diseases such as COVID-19; for example, in Cameroon, a total of 230 species were recorded as potential sources of ingredients for the fight against COVID-19, based on the assumption that these plants have been used to manage at least three of its common symptoms (Fongnzossie-Fedoung et al. 2023). In the Yucatan Peninsula, Mexico, 28 plant species have been reported to treat pre- and post-symptoms of SARS-CoV-2 with 336 bioactive compounds that are related to medicinal properties (Ríos-Oviedo et al. 2025). For the Tenek territory, at least 179 species are used to treat 59 symptoms of DRS and CIPD, which could be studied in more detail to know if they are used to treat some symptoms of COVID-19. Traditional medicine depends on the rich diversity of plants and the knowledge related to their use as phytotherapy (WHO 2013) and it has been essential for maintaining health and well-being in different cultures around the world. Previously, it has been indicated that botanical knowledge, based on systematised herbarium specimens, is essential for the correct identification of plant species to avoid errors in the study of medicinal plants. This prevents nomenclatural errors and facilitates the standardisation of ethnobotanical information by using scientific rather than common names (Cilia-López et al. 2021). Advances in medicinal plant inventories, combining ethnobotanical and ethnotaxonomic information with new systematics, allow the application of new phylogenetic approaches to elucidate the evolutionary history of species, which accelerates the discovery of new drugs for the treatment of different diseases (Cupido et al. 2024).

Documenting medicinal plants could empower local communities, strengthen cultural identity, improve community health solutions, support sustainable development and safeguard biodiversity. It could also inspire new ways to integrate transdisciplinary research in which Indigenous peoples are key stakeholders, contributors, participants and beneficiaries of all incoming revenue. Previously, it was mentioned that medicinal plant species are not merely chemical factories for extraction and exploitation; rather, they are symbiotic partners that have shaped modern societies, improved human health and extended human lifespans (Davis and Choisy 2024). When conducted ethically and collaboratively, knowledge remains rooted in its community context, benefitting the people who have safeguarded it for generations. More recently, Gitima et al. (2025) indicated that understanding the community's knowledge of and dependence on traditional plant-based medicine is essential to creating effective, sustainable conservation strategies that will protect these valuable plant resources in the long term. Sharing of benefits is expected by international accords, especially the Nagoya Protocol, which identified clear policies around access and benefit sharing (ABS) meant to ensure the prior informed consent of Indigenous communities and to guarantee their receipt of benefits (Davis and Choisy 2024). The protocol also encourages scientific research which contributes to the conservation and sustainable use of biological diversity, particularly in developing countries, including through simplified measures on access for non-commercial research purposes. Despite their importance, ethnobotanical data are largely inaccessible. They are not usually digitised and are often buried in unindexed literature and museums. Thus, open-access digital databases, such as the “Catálogo de plantas medicinales de la region biocultural Huasteca” (Cupido et al. 2023, Cupido and De-Nova 2025) are particularly valuable for bioprospecting studies because they synthesise botanical information, traditional knowledge and practices. This is central to identifying medicinal plants and developing conservation strategies that include all stakeholders (see Buenz et al. (2018), Kumar et al. (2021), Davis and Choisy (2024)).

Finally, it has previously been suggested that preserving hotspots of biocultural heritage, particularly in areas of high linguistic diversity, such as those identified in the present research, is essential for preserving bioculturally significant medicinal plant species (Cámara-Leret and Bascompte 2021, Davis and Choisy 2024). It is also important to mention that, like other knowledge systems, traditional systems are plural and layered (see Todd et al. (2023)). Rather than being linear, they are embedded in social life and transmitted through a mosaic of pathways, including oral traditions, ritual practices, women’s networks, apprenticeships, markets, community initiatives, intercultural education and digital platforms. Although some of the information in our database comes from previous research conducted several decades ago in the biocultural region Huasteca, recent studies (Alonso-Castro et al. 2012, Casanova-Pérez et al. 2022), student thesis, visits, interviews with local people and botanical collections show that traditional medicinal knowledge and practices are still widely used and transmitted, not only through traditional methods, but also through new methods, such as intercultural education and digital platforms.

A wide and diverse fraction of biodiversity used as medicine for different cultures highlights the relevance of the persistence of traditional knowledge as an alternative to address major socioeconomic and political changes, related to health services and ultimately emphasises the importance of recognising this knowledge as a part of their biocultural heritage, a result of biological and social convergence over time. Given the great biological and cultural diversity of the Mexican territory, systematised ethnobotanical inventories could highlight regions that are little explored, opening the way for new studies on natural resources and the possibility to apply new evolutionary approaches to improve bioprospecting for the discovery of new drugs and emphasises the importance of preserving biocultural heritage (Cupido et al. 2024). In addition, the characterisation and identification of phytochemical compounds is fundamental not only to contribute to the knowledge of the species and its potential biotechnological applications, but also to improve bio-valorisation, as well as programmes with the population to promote the appropriate use of traditional medicine (Cupido et al. 2022).

## Conclusions

The relevance of cartography in exploring hotspots for bioprospecting and conserving natural ethnomedicinal resources that have known benefits and value for human well-being has not been widely studied. Our synthesis of the existing information on diversity and distribution of medicinal plant species in the Tenek territory reveals geographical zones with a high number of medicinal plant species, classes of diseases treated and ethnomedicinal indices. They represent hotspots for biodiversity conservation and nature option values for future benefits (Futuyama 1995, Faith et al. 2010). The municipalities with the main diversity of ethnomedicinal plant species and classes of diseases (San Antonio, Ciudad Valles, Aquismón, Tantoyuca and Tempoal) could be considered as a natural drugstore with a high variety of valuable options that represent the future benefits provided by biodiversity (Futuyama 1995, Faith et al. 2010). The EI (ethnomedicinal index) allows identifying particular hotspots with the greatest potential to develop bioprospecting activities in Aquismón, San Antonio, Ciudad Valles and Tantoyuca, where medicinal plants are assets highlighting the economic value of biodiversity (Costanza et al. 1997). Public policies and biodiversity conservation strategies are more likely to succeed in the long term if adequate methodologies are used to identify and allocate economic resources to benefit local inhabitants (Wang et al. 2022). The presence of natural protected areas in the biocultural region Huasteca, such as the Sierra del Abra Tanchipa Biosphere Reserve in Ciudad Valles, ensures the conservation of ethnomedicinal resources, where disturbance levels have been previously noted as low (De-Nova et al. 2018) and the participation of locals, government and enterprises have strengths and opportunities to combine bioprospecting and conserving biodiversity and traditional knowledge. Additionally, the future marketing and commercial trade of medicinal plants provide significant opportunities for local communities to profit through initiatives, based on ethical, equitable and sustainable principles. This profit stems from their traditional ethnobotanical knowledge and proximity to the resources, both of which are considered forms of biocultural heritage.

## Supplementary Material

EA967516-47C7-53EF-9F43-E39898C95B31 10.3897/BDJ.13.e170091.suppl1 Supplementary material 1 Table S1 Data type Species and classes of disease Brief description Table S1. Plant species used in the Huastec Mayan traditional medicine, the classes of disease they treat according to ICD-11 and the CBS category and their risk status according to NOM-059 and IUCN. Certain infectious or parasitic diseases (CIPD), diseases of the blood or blood-forming organs (DBBO), diseases of the circulatory system (DCS), diseases of the digestive system (DGS), diseases of the ear or mastoid process (DEMP), diseases of the visual system (DVS), diseases of the genitourinary system (DGUS), diseases of the musculoskeletal system or connective tissue (DMSC), diseases of the nervous system (DNS), diseases of the respiratory system (DRS), diseases of the skin (DS), endocrine, nutritional or metabolic diseases (ENMD), factors influencing health status or contact with health services (FIHS), injury, poisoning or certain other consequences of external causes (IPEC), mental, behavioural or neurodevelopmental disorders (MBND), neoplasms (NE), pregnancy, childbirth or the puerperium (PCP) and symptoms, signs or clinical findings not elsewhere classified (NEC). Additionally, we included culture-bound syndromes (CBS). File: oo_1472730.pdf https://binary.pensoft.net/file/1472730 Madeleyne Cupido & Arturo De-Nova

BE58DA85-39F2-52FD-ADFC-00C0BD559591 10.3897/BDJ.13.e170091.suppl2 Supplementary material 2 Table S2 Data type Plant species Brief description Table S2. Database of the plant species used in the Huastec Mayan traditional medicine, taxonomy, common names, origin, growth forms, habitat, risk status according to NOM-059 and IUCN, classes of disease they treat according to ICD-11 and the CBS category. File: oo_1472731.pdf https://binary.pensoft.net/file/1472731 Madeleyne Cupido & Arturo De-Nova

BF8CCDA3-D5BF-51F6-A6D8-2ECC9DCDEEDC 10.3897/BDJ.13.e170091.suppl3 Supplementary material 3 Table S3 Data type Occurrences Brief description Geographical data from records of the vascular plant species with ethnomedicinal information documented in the Tenek territory (https://doi.org/10.5281/zenodo.15798528). File: oo_1472732.csv https://binary.pensoft.net/file/1472732 Madeleyne Cupido & Arturo De-Nova

## Figures and Tables

**Table 1.** Overview of the disease classes treated by the Huastec Mayan medicinal plants according to ICD-11 (2024) and the CBS category.

Disease class	Diseases and symptoms	No. species
Certain infectious or parasitic diseases (CIPD)	30	135
Diseases of the blood or blood-forming organs (DBBO)	11	24
Diseases of the circulatory system (DCS)	14	32
Diseases of the digestive system (DGS)	44	161
Diseases of the ear or mastoid process (DEMP)	5	19
Diseases of the visual system (DVS)	11	15
Diseases of the genitourinary system (DGUS)	15	64
Diseases of the musculoskeletal system or connective tissue (DMSC)	26	52
Diseases of the nervous system (DNS)	4	16
Diseases of the respiratory system (DRS)	29	75
Diseases of the skin (DS)	65	177
Endocrine, nutritional or metabolic diseases (ENMD)	3	13
Factors influencing health status or contact with health services (FIHS)	3	10
Injury, poisoning or certain other consequences of external causes (IPEC)	19	74
Mental, behavioural or neurodevelopmental disorders (MBND)	15	26
Neoplasms (NE)	3	3
Pregnancy, childbirth or the puerperium (PCP)	23	71
Symptoms, signs or clinical findings, not elsewhere classified (NEC)	56	270
Culture-bound syndromes (CBS)	40	137

## References

[B13416030] Alcorn Janis B. (1984). Huastec Mayan ethnobotany.

[B13630869] Alonso-Castro AJ,, Maldonado-Miranda JJ, Zarate-Martinez A, del Rosario Jacobo-Salcedo M (2012). Medicinal plants used in the Huasteca Potosina, México. Journal of ethnopharmacology.

[B13589225] Argueta A,, Cano A, Rodarate ME (1994). Atlas de las Plantas Medicinales de la Medicina Tradicional Mexicana.. Instituto Nacional Indigenista, Mexico, DF.

[B13416286] Barthas B, (1996). De la selva al naranjal (transformaciones en la agricultura indígena en la Huasteca potosina).

[B13415549] Beroni Shenell, Goldson-Barnaby Andrea, Petrea Facey (2023). A mini review of *Desmodium
incanum* - An underutilized herb in Jamaica. Caribbean Journal of Science.

[B13415558] Bessada Sílvia M. F., Barreira João C. M., Oliveira M. Beatriz P. P. (2015). Asteraceae species with most prominent bioactivity and their potential applications: A review. Industrial Crops and Products.

[B13415567] Blanckaert Isabelle, Paredes-Flores Martín, Espinosa-García Francisco J., Piñero Daniel, Lira Rafael (2012). Ethnobotanical, morphological, phytochemical and molecular evidence for the incipient domestication of Epazote (*Chenopodium
ambrosioides* L.: Chenopodiaceae) in a semi-arid region of Mexico. Genetic Resources and Crop Evolution.

[B13415577] Boyle Brad, Hopkins Nicole, Lu Zhenyuan, Raygoza Garay Juan Antonio, Mozzherin Dmitry, Rees Tony, Matasci Naim, Narro Martha L., Piel William H., Mckay Sheldon J., Lowry Sonya, Freeland Chris, Peet Robert K., Enquist Brian J. (2013). The taxonomic name resolution service: an online tool for automated standardization of plant names. BMC Bioinformatics.

[B13630851] Buenz EJ,, Verpoorte R, and Bauer BA (2018). The ethnopharmacologic contribution to bioprospecting natural products. Annual Review of Pharmacology and Toxicology.

[B13589181] Bye R,, Ramamorthy TP, Bye R, Lot A, Fa J (1993). Biological diversity of Mexico. Origins and distribution..

[B13416046] Bye Robert, Linares Edelmira, Estrada Eric, Springer (1995). Phytochemistry of Medicinal Plants.

[B13606765] Caballero-George C,, Gupta MP, (2011). A quarter century of pharmacognostic research on Panamanian flora: a review. Planta Medica.

[B13416059] Caballero J, Cortés L, Martínez-Alfaro MA, Lira-Saade R (2004). Uso y manejo tradicional de la diversidad vegetal, In: Biodiversidad de Oaxaca.

[B13590403] Cámara-Leret R,, Bascompte J, (2021). Language extinction triggers the loss of unique medicinal knowledge. Proceedings of the National Academy of Sciences.

[B13630886] Casanova-Pérez C,, Delgado-Caballero CE, Cruz-Bautista P, Casanova-Pérez L (2022). Plantas medicinales usadas por los Tének en la Huasteca, México. CienciaUAT.

[B13416203] Chamkhi Imane, Hnini Mohamed, Aurag Jamal (2022). Conventional medicinal uses, phytoconstituents, and biological activities of *Euphorbia
officinarum* L.: A systematic review. Advances in Pharmacological and Pharmaceutical Sciences.

[B13415596] Chidambaram Kumarappan, Alqahtani Taha, Alghazwani Yahia, Aldahish Afaf, Annadurai Sivakumar, Venkatesan Kumar, Dhandapani Kavitha, Thilagam Ellappan, Venkatesan Krishnaraju, Paulsamy Premalatha, Vasudevan Rajalakshimi, Kandasamy Geetha (2022). Medicinal plants of *Solanum* species: The promising sources of phyto-insecticidal compounds. Journal of Tropical Medicine.

[B13415613] Cilia-López Virginia Gabriela, Cariño-Cortés Raquel, Zurita-Salinas Luis Ricardo, Cilia-López Virginia Gabriela, Cariño-Cortés Raquel, Zurita-Salinas Luis Ricardo (2021). Ethnopharmacology of the Asteraceae family in Mexico. Botanical Sciences.

[B13606729] Coe FG,, Anderson GJ, (2005). Snakebite ethnopharmacopoeia of eastern Nicaragua. Journal of Ethnopharmacology.

[B13589234] CONABIO (2006). Capital natural y bienestar social.

[B13415624] Costanza Robert, d'Arge Ralph, de Groot Rudolf, Farber Stephen, Grasso Monica, Hannon Bruce, Limburg Karin, Naeem Shahid, O'Neill Robert V., Paruelo Jose, Raskin Robert G., Sutton Paul, van den Belt Marjan (1997). The value of the world's ecosystem services and natural capital. Nature.

[B13416067] Cox Paul Alan, Symposium Ciba Foundation (1994). Ciba Foundation Symposium 185 - Ethnobotany and the Search for New Drugs.

[B13588703] Cruz-Pérez AL,, Barrera-Ramos J, Bernal-Ramírez L, Bravo-Avilez D (2021). Actualized inventory of medicinal plants used in traditional medicine in Oaxaca, Mexico. Journal of Ethnobiology and Ethnomedicine.

[B13416294] Cupido Madeleyne, De-Nova Arturo, Guerrero-González María L., Pérez-Vázquez Francisco Javier, Méndez-Rodríguez Karen Beatriz, Delgado-Sánchez Pablo (2022). GC-MS analysis of phytochemical compounds of *Opuntia
megarrhiza* (Cactaceae), an endangered plant of Mexico. PeerJ Organic Chemistry.

[B13416238] Cupido M., De-Nova J. A., Cilia-Lopez V. G. (2023). Catálogo de plantas medicinales de la región biocultural Huasteca. https://biocultural.uaslp.mx/.

[B13415651] Cupido M., De-Nova J. A., Cilia-López V. G. (2024). Aproximaciones evolutivas en etnobotánica de plantas medicinales y bioprospección. Botanical Sciences.

[B13415642] Cupido Madeleyne, De-Nova J A (2025). Catálogo de plantas medicinales de la región biocultural Huasteca. Revista Universitarios Potosinos.

[B13630811] Davis CC,, Choisy P, (2024). Medicinal plants meet modern biodiversity science. Current biology.

[B13415668] De Lima Luciene Ferreira, Andrade-Pinheiro Jacqueline Cosmo, Freitas Maria Audilene, da Silva Adriely Idalina>, Fonseca Victor Juno Alencar, da Silva Taís Gusmão, da Silva Josefa Carolaine Pereira, de Lima Rosilaine Honorato, Sales Débora Lima, Neves Rejane Pereira, de Brito Edy Sousa, Ribeiro Paulo Riceli Vasconcelos, Canuto Kirley Marques, Coutinho Henrique Douglas Melo, Siyadatpanah Abolghasem, Kim Bonglee, Morais-Braga Maria Flaviana Bezerra (2022). Anti-Candida properties of *Gossypium
hirsutum* L.: Enhancement of fungal growth, biofilm production and antifungal resistance. Pharmaceutics.

[B13607065] De-Nova JA,, De-Nova E, Flores Rivas J, Reyes-Hernández H, Guevara Sada S,, Moreno-Casasola P (2025). La deforestación, la fragmentación y la conectividad.

[B13416080] De-Nova J A, Castillo-Lara Pedro, Salinas Rodríguez M M, Fortanelli-Martínez J., Mora-Olivo A, Reyes-Hernández H. (2018). Reserva de la Biosfera Sierra del Abra Tanchipa. Biodiversidad y acciones para su conservación.

[B13416159] De-Nova José Arturo, Villegas-Ortega Daniela Sofía, Cupido Madeleyne, Cilia-López Virginia Gabriela (2024). Evolutionary clustering in neotropical biocultural heritage: the Huastec Mayan useful plants. Botanical Journal of the Linnean Society.

[B13415690] Domínguez Xorge A., Alcorn Janis B. (1985). Screening of medicinal plants used by Huastec Mayans of northeastern Mexico. Journal of Ethnopharmacology.

[B13415699] Elizalde-Romero Cristina Alicia, Montoya-Inzunza Luis Aurelio, Contreras-Angulo Laura Aracely, Heredia J. Basilio, Gutiérrez-Grijalva Erick Paul (2021). *Solanum* fruits: phytochemicals, bioaccessibility and bioavailability, and their relationship with their health-promoting effects. Frontiers in Nutrition.

[B13415709] Ernst Madeleine, Grace Olwen M., Saslis-Lagoudakis C. Haris, Nilsson Niclas, Simonsen Henrik Toft, Rønsted Nina (2015). Global medicinal uses of *Euphorbia* L. (Euphorbiaceae). Journal of Ethnopharmacology.

[B13415720] Faith Daniel P, Magallón Susana, Hendry Andrew P, Conti Elena, Yahara Tetsukazu, Donoghue Michael J (2010). Evosystem services: an evolutionary perspective on the links between biodiversity and human well-being. Current Opinion in Environmental Sustainability.

[B13415731] Fidler Brooke, Goldberg Tamara (2014). Ingenol Mebutate Gel (Picato). Pharmacy and therapeutics.

[B13415740] Fongnzossie-Fedoung E,, Biwole Achille Bernard, Nyangono Biyegue Christine Fernande, Ngansop Tounkam Marlene, Akono Ntonga Patrick, Nguiamba Véronique Priscille, Essono Damien Marie, Forbi Funwi Preasious, Tonga Calvin, Nguenang Guy Merlin, Kemeuze Victor, Sonwa Denis Jean, Tsabang Nole, Bouelet Isabelle Sandrine, Tize Zra, Boum Alexandre Teplaira, Momo Solefack Marie Caroline, Betti Jean Lagarde, Nouga Bissoue Achille, Lehman Leopold Gustave, Mapongmetsem Pierre Marie, Nneme Nneme Leandre, Ngono Ngane Rosalie Annie, Ngogang Yonkeu Jeanne (2023). A review of Cameroonian medicinal plants with potentials for the management of the COVID-19 pandemic. Advances in Traditional Medicine.

[B13415769] Forest Félix, Grenyer Richard, Rouget Mathieu, Davies T. Jonathan, Cowling Richard M., Faith Daniel P., Balmford Andrew, Manning John C., Procheş Şerban, van der Bank Michelle, Reeves Gail, Hedderson Terry A. J., Savolainen Vincent (2007). Preserving the evolutionary potential of floras in biodiversity hotspots. Nature.

[B13416313] Futuyama D. J (1995). The uses of evolutionary biology. Science.

[B13603975] García-Willis CE,, Vela-Ortega R, Maya-Leal ME (2009). Epidemiología de la mordedura por ofidio en pacientes pediátricos.. Boletín Médico del Hospital Infantil de México.

[B13604001] Garnatje T,, Peñuelas J, Vallès J (2017). Ethnobotany, Phylogeny, and 'Omics' for Human Health and Food Security. Trends in Plant Science.

[B13416094] GBIF (2024). Global Biodiversity Information. https://www.gbif.org/.

[B13630842] Gitima G,, Gebre A, Berhanu Y, Wato T (2025). Exploring indigenous wisdom: Ethnobotanical documentation and conservation of medicinal plants in Goba District, Southwest Ethiopia. Scientific African.

[B13416102] Hamada Fatma A, Hamed Arafa I, Sheded Mohamed G, Shaheen Abdel Samai M (2010). Macro, micro-morphological and bioactivity aspects of naturalized exotic *Solanum
diphyllum* L.. Al-Azhar Bulletin of Science.

[B13606747] Hasan MN,, Azam MNK, Ahmed MN, Hirashima A (2015). A randomized ethnomedicinal survey of snakebite treatment in southwestern parts of Bangladesh. Journal of Traditional and Complementary Medicine.

[B13606692] Hernandez MR,, Avila-Bello CH, Morales-Mavil JE (2007). Etnobotánica y ecología de plantas utilizadas por tres curanderos contra la mordedura de serpiente en la región de Acayucan, Veracruz, México. Boletin de la Sociedad Botanica de Mexico.

[B13415795] Huang Jihong, Chen Bin, Liu Canran, Lai Jiangshan, Zhang Jinlong, Ma Keping (2012). Identifying hotspots of endemic woody seed plant diversity in China. Diversity and Distributions.

[B13415806] Huang L, Peng Huasheng, Xiao Peigen (2011). Development trend of traditional Chinese medicine resources. Zhongguo Zhong Yao Za Zhi.

[B13416254] ICD-11 (2024). International Classification of Diseases (ICD-11) of the World Health Organization. https://www.who.int/standards/classifications/classification-of-diseases.

[B13588991] Ijatuyi EJ,, Lamm A, Yessoufou K, Suinyuy T (2025). Integration of indigenous knowledge with scientific knowledge: A systematic review. Environmental Science & Policy. Environmental Science & Policy.

[B13416185] Islam MS, Ara H, Ahmad KI, Uddin MM (2019). A review on medicinal uses of different plants of Euphorbiaceae family. Universal Journal of Pharmaceutical Research.

[B13416111] IUCN (2023). The IUCN Red List of Threatened Species. https://www.iucnredlist.org/en.

[B13606756] Kadir MF,, Karmoker JR, Alam MR, Jahan SR (2015). Ethnopharmacological survey of medicinal plants used by traditional healers and indigenous people in Chittagong Hill Tracts, Bangladesh, for the treatment of snakebite. Evidence-Based Complementary and Alternative Medicine.

[B13630860] Kumar A,, Kumar S, Ramchiary N, Singh P (2021). Role of traditional ethnobotanical knowledge and indigenous communities in achieving sustainable development goals. Sustainability.

[B13415843] Kurian Gino A., Paddikkala Jose (2010). Oral delivery of insulin with *Desmodium
gangeticum* root aqueous extract protects rat hearts against ischemia reperfusion injury in streptozotocin induced diabetic rats. Asian Pacific Journal of Tropical Medicine.

[B13415852] Leija-Loredo Edgar G., Reyes-Hernández Humberto, Fotanelli-Martínez Javier, Palacio-Aponte Gerardo (2011). Situación actual del bosque de niebla en el estado de San Luis Potosí, México. Investigación y Ciencia de la Universidad Autónoma de Aguascalientes.

[B13415861] Leonti Marco (2011). The future is written: Impact of scripts on the cognition, selection, knowledge and transmission of medicinal plant use and its implications for ethnobotany and ethnopharmacology. Journal of Ethnopharmacology.

[B13588681] Loraine S., Mendoza-Espinoza JA, (2010). Las plantas medicinales en la lucha contra el cáncer, relevancia para México. Revista Mexicana de Ciencias Farmacéutica.

[B13589155] Martin G,, Camacho-Benavides CI, Del Campo-García CA, Anta-Fonseca S (2011). Indigenous and community conserved areas in Oaxaca, Mexico. J Environ Manage.. Journal of Environmental Management.

[B13415870] Moremi Matenyane P., Makolo Felix, Viljoen Alvaro M., Kamatou Guy P. (2021). A review of biological activities and phytochemistry of six ethnomedicinally important South African *Croton* species. Journal of Ethnopharmacology.

[B13415879] Muanda François Nsemi, Bouayed Jaouad, Djilani Abdelouaheb, Yao Chunyan, Soulimani Rachid, Dicko Amadou (2011). Chemical composition and, cellular evaluation of the antioxidant activity of *Desmodium
adscendens* eaves. Evidence-Based Complementary and Alternative Medicine.

[B13588759] Muñetón-Pérez P, (2009). Plantas medicinales: un complemento vital para la salud de los mexicanos. Entrevista con el Dr. Erick Estrada Lugo.. Revista Digital Universitaria.

[B13415890] Myers Norman, Mittermeier Russell A., Mittermeier Cristina G., da Fonseca Gustavo A. B., Kent Jennifer (2000). Biodiversity hotspots for conservation priorities. Nature.

[B13607028] Nelson LS,, Shih MJ, Balick MJ (2007). Handbook of Poisonous and Injurious Plants.

[B13415834] Omale J, Friday Emmanuel T (2010). Phytochemical composition, bioactivity and wound healing potential of *Euphorbia
heterophylla* (Euphorbiaceae) leaf extract. International Journal on Pharmaceutical and Biomedical Research.

[B13415900] Pandey Aseesh, Chandra Sekar K., Joshi Bhaskar, Rawal R. S. (2018). Threat assessment of high-value medicinal plants of cold desert areas in Johar valley, Kailash Sacred Landscape, India. Plant Biosystems - An International Journal Dealing with all Aspects of Plant Biology.

[B13416143] Pironon S, Ondo I, Diazgranados M, Allkin R, Baquero AC, Cámara-Leret R, Canteiro C, Dennehy-Carr Z, Govaerts R, Hargreaves S, Hudson AJ (2024). The global distribution of plants used by humans.. Science.

[B13416119] POWO (2025). Plants of the World Online | Kew Science. https://powo.science.kew.org/.

[B13416270] QGIS (2025). Quantum GIS development team. https://qgis.org/community/organisation/.

[B13416411] Reyes Hernández Humberto, Aguilar Robledo Miguel, Aguirre Rivera Juan Rogelio, Trejo Vázquez Irma (2006). Cambios en la cubierta vegetal y uso del suelo en el área del proyecto Pujal-Coy, San Luis Potosí, México, 1973-2000. Investigaciones Geográficas.

[B13415926] Ríos-Oviedo Ángel, Cetzal-Ix William, López-Castilla Héctor M. J., Basu Saikat Kumar, Tamayo-Cen Iván, De la Torre Espinosa Zamaria (2025). Ethnomedicinal uses of the flora as an alternative during the treatment of SARS-CoV-2 in the Mayan communities of the Yucatan Peninsula, Mexico. Journal of Herbs, Spices & Medicinal Plants.

[B13416194] Rolnik A, Olas B (2021). The plants of the Asteraceae family as agents in the protection of human health. International Journal of Molecular Sciences.

[B13415952] Roopashree S., Anitha J., Challa Suryateja, Mahesh T. R., Venkatesan Vinoth Kumar, Guluwadi Suresh (2024). Mapping of soil suitability for medicinal plants using machine learning methods. Scientific Reports.

[B13606720] Samy RP,, Thwin MM, Gopalakrishnakone P, Ignacimuthu S (2008). Ethnobotanical survey of folk plants for the treatment of snakebites in Southern part of Tamilnadu, India. Journal of Ethnopharmacology.

[B13606671] Sanz-Biset J,, Campos-de-la-Cruz J, Epiquién-Rivera MA, Cañigueral S (2009). A first survey on the medicinal plants of the Chazuta valley (Peruvian Amazon). Journal of Ethnopharmacology.

[B13416246] SEMARNAT (2010). Norma Oficial Mexicana NOM-059-SEMARNAT-2010. https://www.dof.gob.mx/normasOficiales/4254/semarnat/semarnat.htm.

[B13416262] SLPM (2025). Herbario Isidro Palacios - SLPM - UASLP. https://slpm.uaslp.mx/.

[B13415970] Tangjitman Kornkanok, Wongsawad Chalobol, Kamwong Kaweesin, Sukkho Treetip, Trisonthi Chusie (2015). Ethnomedicinal plants used for digestive system disorders by the Karen of northern Thailand. Journal of Ethnobiology and Ethnomedicine.

[B13603961] Tay ZJ,, Sánchez JGD, Vega JTS, Sánchez DR (2002). Serpientes y reptiles de importancia médica en México. Revista de la Facultad de Medicina UNAM.

[B13630913] Todd WF,, Towne CE, Clarke JB (2023). Importance of centering traditional knowledge and Indigenous culture in geoscience education. Journal of Geoscience Education.

[B13416135] Toledo Víctor M., Barrera-Bassols N, (2008). La memoria biocultural: la importancia ecológica de la sabidurías tradicionales.

[B13415986] Tsioutsiou Efthymia Eleni, Amountzias Vaios, Vontzalidou Argyro, Dina Evanthia, Stevanović Zora Dajić, Cheilari Antigoni, Aligiannis Nektarios (2022). Medicinal plants used traditionally for skin related problems in the South Balkan and East Mediterranean region—A review. Frontiers in Pharmacology.

[B13606738] Vásquez J,, Jiménez SL, Gómez IC, Rey JP (2013). Snakebites and ethnobotany in the eastern region of Antioquia, Colombia—the traditional use of plants. Journal of Ethnopharmacology.

[B13416005] Wang Yaping, Fahad Shah, Wei Liqian, Luo Bowen, Luo Jianchao (2022). Assessing the role of financial development and financial inclusion to enhance environmental sustainability: Do financial inclusion and eco-innovation promote sustainable development?. Frontiers in Environmental Science.

[B13416230] WHO (2013). Traditional medicine. https://apps.who.int/gb/ebwha/pdf_files/EB134/B134_24-en.pdf.

[B13416017] Zhu Zhan-Zhou, Ma Ke-Jia, Ran Xia, Zhang Hong, Zheng Cheng-Jian, Han Ting, Zhang Qiao-Yan, Qin Lu-Ping (2011). Analgesic, anti-inflammatory and antipyretic activities of the petroleum ether fraction from the ethanol extract of *Desmodium
podocarpum*. Journal of Ethnopharmacology.

