# Supplementary material for: Diversity and distribution of the Huastec Mayan medicinal plants: hotspots for bioprospecting and conservation
Source: Biodivers Data J. 2025 Dec 8;13:e170091. doi: 10.3897/BDJ.13.e170091 (PMC12706493; doi:10.3897/BDJ.13.e170091)
Supplement: Supplementary material 1 — Table S1 [file bdj-13-e170091-s001.pdf]

Table S1. Plant species used in the Huastec Mayan traditional medicine, the classes of disease they treat according to ICD-11 of WHO (2024) and the CBS category, and their risk status according to NOM-059 and IUCN. Certain infectious or parasitic diseases (CIPD), diseases of the blood or blood-forming organs (DBBO), diseases of the circulatory system (DCS), diseases of the digestive system (DGS), diseases of the ear or mastoid process (DEMP), diseases of the visual system (DVS), diseases of the genitourinary system (DGUS), diseases of the musculoskeletal system or connective tissue (DMSC), diseases of the nervous system (DNS), diseases of the respiratory system (DRS), diseases of the skin (DS), endocrine, nutritional or metabolic diseases (ENMD), factors influencing health status or contact with health services (FIHS), injury, poisoning or certain other consequences of external causes (IPEC), mental, behavioral or neurodevelopmental disorders (MBND), neoplasms (NE), pregnancy, childbirth or the puerperium (PCP), and symptoms, signs or clinical findings not elsewhere classified (NEC). Additionally, we included culture-bound syndromes (CBS).

**Family**	**Species**	**Author**	**Classes of disease**	**Symptoms and diseases**	**Risk category (NOM-059 / IUCN)**
Acanthaceae	*Aphelandra* sp.		CBS	1	
Acanthaceae	*Dianthera pectoralis*	(Jacq.) J.F.Gmel.	CBS, NEC, DGS	5	
Acanthaceae	*Dicliptera sexangularis*	(L.) Juss.	CBS, NEC, PCP	3	
Acanthaceae	*Elytraria bromoides*	Oerst.	DEMP	1	
Acanthaceae	*Justicia brandegeeana*	Wassh. & L.B.Sm.	CBS, NEC, DGS, PCP, CIPD, NE	11	
Acanthaceae	*Justicia fulvicoma*	Schltdl. & Cham.	NEC, DGUS, CIPD	4	
Acanthaceae	*Justicia spicigera*	Schltdl.	CBS, DBBO, NEC, PCP, DGUS, DRS, CIPD	20	
Acanthaceae	*Odontonema callistachyum*	(Schltdl. & Cham.) Kuntze	NEC, PCP	2	
Acanthaceae	*Odontonema tubaeforme*	(Bertol.) Kuntze	PCP	2	
Acanthaceae	*Ruellia ciliatiflora*	Hook.	NEC, IPEC	2	
Acanthaceae	*Ruellia simplex*	C.Wright	CBS, NEC, CIPD	9	
Acanthaceae	*Thunbergia alata*	Bojer ex Sims	NEC	1	
Acanthaceae	*Thunbergia fragrans*	Roxb.	NEC	1	
Agdestidaceae	*Agdestis clematidea*	Moc. & Sessé ex DC.	CBS, DS, IPEC, DMSC	7	
Alstroemeriaceae	*Bomarea edulis*	(Tussac) Herb.	DS, DGS, MBND, ENMD	5	
Amaranthaceae	*Achyranthes aspera*	L.	CIPD	1	

Amaranthaceae	*Alternanthera philoxeroides*	(Mart.) Griseb.	DS, NEC, CIPD	6	
Amaranthaceae	*Alternanthera sessilis*	(L.) R.Br. ex DC.	NEC, CIPD	4	LC
Amaranthaceae	*Amaranthus caudatus*	L.	NEC, DGS	3	
Amaranthaceae	*Amaranthus hybridus*	L.	DS	2	
Amaranthaceae	*Dysphania ambrosioides*	(L.) Mosyakin & Clemants	NEC, DGS, PCP, CIPD, IPEC	7	
Amaranthaceae	*Gomphrena globosa*	L.	CIPD	1	
Amaryllidaceae	*Allium longifolium*	(Kunth) Spreng.	DS, NEC, IPEC, DMSC	5	
Amaryllidaceae	*Allium sativum*	L.	CBS, NEC, DGS, DS, CIPD, DMSC	6	
Amaryllidaceae	*Hymenocallis sp.*		CIPD	1	
Anacardiaceae	*Spondias purpurea*	L.	DS, DGS, CIPD	7	LC
Anemiaceae	*Anemia adiantifolia*	(L.) Sw.	NEC	1	
Anemiaceae	*Anemia mexicana*	Klotzsch	NEC	2	
Annonaceae	*Annona globiflora*	Schltdl.	CBS, DS, NEC, IPEC	9	
Annonaceae	*Annona reticulata*	L.	CBS, NEC, DGUS, CIPD, NE	8	LC
Apiaceae	*Coriandrum sativum*	L.	DGS	1	
Apiaceae	*Cyclospermum leptophyllum*	(Pers.) Sprague	CIPD	2	
Apiaceae	*Eryngium sp.*		CIPD	1	
Apocynaceae	*Asclepias curassavica*	L.	CBS, DS, DGS, CIPD	5	
Apocynaceae	*Cascabela thevetia*	(L.) Lippold	DCS, DS, DGS	4	LC
Apocynaceae	*Echites panduratus*	A.DC.	NEC, DGS, ENMD	5	
Apocynaceae	*Echites tuxtlensis*	Standl.	NEC, DGS, DRS, IPEC	7	
Apocynaceae	*Gonolobus niger*	(Cav.) Schult.	IPEC	1	
Apocynaceae	*Pentalinon andrieuxii*	(Müll.Arg.) B.F.Hansen & Wunderlin	DS, CIPD, IPEC	5	
Apocynaceae	*Plumeria rubra*	L.	CBS, DS	2	LC
Apocynaceae	*Rauvolfia tetraphylla*	L.	NEC	1	LC
Apocynaceae	*Ruehssia macrophylla*	(Humb. & Bonpl. ex Schult.) H.Karst.	IPEC	1	
Apocynaceae	*Tabernaemontana alba*	Mill.	DS, DGS, IPEC	4	LC
Araceae	*Syngonium podophyllum*	Schott	CIPD	1	
Araceae	*Xanthosoma robustum*	Schott	DS	1	
Araceae	*Xanthosoma sagittifolium*	(L.) Schott	DS, NEC	2	

Araliaceae	*Dendropanax arboreus*	(L.) Decne. & Planch.	CBS, DS, NEC, DRS, DMSC	8	
Arecaceae	*Acrocomia aculeata*	(Jacq.) Lodd. ex Mart.	DRS, CIPD	2	LC
Arecaceae	*Chamaedorea elegans*	Mart.	MBND, CIPD	2	
Arecaceae	*Sabal mexicana*	Mart.	CBS, DS, NEC, DGS, CIPD	6	LC
Aristolochiaceae	*Aristolochia littoralis*	Parodi	DGS, DRS	2	
Aristolochiaceae	*Aristolochia orbicularis*	Duch.	DS, DRS, DMSC	5	
Aristolochiaceae	*Aristolochia sp.*		DS, NEC	2	
Asparagaceae	*Agave sp.*		CBS, NEC, PCP, DGUS, DRS, CIPD, IPEC	9	
Asparagaceae	*Agave variegata*	Jacobi	DS, NEC	3	
Asparagaceae	*Beschorneria sp.*		NEC, IPEC	2	
Asparagaceae	*Echeandia reflexa*	(Cav.) Rose	DS, NEC, DGS, DGUS	5	
Asparagaceae	*Yucca gigantea*	Lem.	CBS, DEMP, PCP	4	
Asphodelaceae	*Aloe vera*	(L.) Burm.f.	CBS, DS, CIPD, IPEC	6	
Aspleniaceae	*Asplenium pumilum*	Sw.	DCS, ENMD	3	
Asteraceae	*Aldama dentata*	La Llave ex La Llave	DS, DRS	2	
Asteraceae	*Anthemis sp.*		CBS, DGS	2	
Asteraceae	*Artemisia ludoviciana*	Nutt.	DS, DEMP, NEC, CIPD, IPEC	8	
Asteraceae	*Bidens pilosa*	L.	DS, NEC, DGS, DRS	5	
Asteraceae	*Bidens reptans*	G.Don	DS, NEC	3	
Asteraceae	*Brickellia diffusa*	(Vahl) A.Gray	DVS	1	
Asteraceae	*Calea ternifolia*	Kunth	DS, NEC, DRS, CIPD	9	
Asteraceae	*Carduus sp.*		NEC, DS, DRS, DMSC	5	
Asteraceae	*Chaptalia nutans*	(L.) Polák	NEC, CIPD	2	
Asteraceae	*Chromolaena collina*	(DC.) R.M.King & H.Rob.	CBS, DBBO, DS, NEC, DGS, PCP	8	LC
Asteraceae	*Chromolaena odorata*	(L.) R.M.King & H.Rob.	CBS, NEC, DRS	6	
Asteraceae	*Cirsium sp.*		NEC, DRS, CIPD	5	
Asteraceae	*Critonia morifolia*	(Mill.) R.M.King & H.Rob.	NEC, DGS, DGUS	4	LC
Asteraceae	*Critonia quadrangularis*	(DC.) R.M.King & H.Rob.	NEC, DS, DGUS, CIPD, DMSC	7	LC
Asteraceae	*Erechtites hieracifolia*	(L.) Raf.	CBS, DBBO, NEC	3	

Asteraceae	*Hymenostephium cordatum*	S.F.Blake	IPEC	2	
Asteraceae	*Koanophyllon albicaulis*	(Sch.Bip. ex Klatt) R.M.King & H.Rob.	CBS, DBBO, NEC, DGS, DRS	21	
Asteraceae	*Lagascea helianthifolia*	Kunth	FIHS	1	
Asteraceae	*Loxothysanus pedunculatus*	Rydb.	CBS, NEC	3	
Asteraceae	*Mikania cordifolia*	(L.f.) Willd.	CIPD, IPEC	2	
Asteraceae	*Neurolaena lobata*	(L.) R.Br. ex Cass.	PCP	1	
Asteraceae	*Parthenium hysterophorus*	L.	DS, NEC, DGS, DNS, PCP, CIPD	10	
Asteraceae	*Pluchea odorata*	(L.) Cass.	CBS, NEC, DGS, DS, PCP, DRS, DMSC	14	
Asteraceae	*Porophyllum ruderale subsp. macrocephalum*	(DC.) R.R.Johnson	DBBO, NEC, DGS	6	
Asteraceae	*Pseudogynoxys chenopodioides*	(Kunth) Cabrera	NEC	1	
Asteraceae	*Salmea scandens*	(L.) DC.	DS, DGS, DRS	4	
Asteraceae	*Tagetes erecta*	L.	CBS, DS, NEC, PCP, DRS, ENMD, CIPD, DMSC	22	
Asteraceae	*Tagetes lucida*	Cav.	CBS	2	
Asteraceae	*Trixis inula*	Crantz	CBS, DS, NEC, PCP, DRS, IPEC	10	LC
Asteraceae	*Verbesina persicifolia*	DC.	CBS, DS, DEMP, NEC, DGS, IPEC	18	
Balsaminaceae	*Impatiens walleriana*	Hook.f.	PCP	1	
Basellaceae	*Anredera vesicaria*	(Lam.) C.F.Gaertn.	CBS, NEC, DS, IPEC, DMSC	7	
Begoniaceae	*Begonia barkeri*	Knowles & Westc.	CIPD	1	
Begoniaceae	*Begonia incarnata*	Link & Otto	NEC, DGUS	2	
Begoniaceae	*Begonia wallichiana*	Lehm.	NEC, DGS, ENMD	3	
Bignoniaceae	*Amphilophium crucigerum*	(L.) L.G.Lohmann	DS	1	
Bignoniaceae	*Bignonia potosina*	(K.Schum. & Loes.) L.G.Lohmann	NEC, CIPD	2	
Bignoniaceae	*Crescentia alata*	Kunth	CBS, IPEC	3	LC
Bignoniaceae	*Dolichandra quadrivalvis*	(Jacq.) L.G.Lohmann	MBND	1	
Bignoniaceae	*Parmentiera aculeata*	(Kunth) Seem.	DCS, DEMP, NEC, DGS, DS, DGUS, DRS, CIPD, IPEC, DMSC	24	LC

Bignoniaceae	*Tabebuia rosea*	(Bertol.) Bertero ex A.DC.	CBS, DS, NEC, DGS, NE, IPEC	7	LC
Bignoniaceae	*Tecoma stans*	(L.) Juss. ex Kunth	ENMD	1	LC
Boraginaceae	*Cordia alliodora*	(Ruiz & Pav.) Oken	DVS	1	LC
Boraginaceae	*Ehretia anacua*	(Terán & Berland.) I.M.Johnst.	CBS, DS, NEC, DGS, DGUS, FIHS, CIPD, IPEC	16	LC
Boraginaceae	*Ehretia tinifolia*	L.	DGS, CIPD	2	
Boraginaceae	*Heliotropium angiospermum*	Murray	DGS, PCP, IPEC	4	
Boraginaceae	*Heliotropium verdcourtii*	Craven	DS, NEC	2	
Boraginaceae	*Tournefortia glabra*	L.	CIPD	1	LC
Brassicaceae	*Cardamine flaccida*	Cham. & Schltdl.	CBS	1	
Brassicaceae	*Lepidium virginicum*	L.	NEC	1	
Bromeliaceae	*Aechmea bracteata*	(Sw.) Griseb.	DCS, DVS, NEC, DGS, CIPD	10	
Bromeliaceae	*Ananas comosus*	(L.) Merr.	DGS	1	
Bromeliaceae	*Tillandsia schiedeana*	Steud.	NEC	2	
Bromeliaceae	*Tillandsia usneoides*	(L.) L.	CBS, DS, DGS, FIHS, DRS, CIPD	7	LC
Burseraceae	*Bursera graveolens*	(Kunth) Triana & Planch.	CBS, NEC, CIPD	8	LC
Burseraceae	*Bursera simaruba*	(L.) Sarg.	DS, NEC, DNS, PCP, CIPD, DMSC	10	LC
Burseraceae	*Protium copal*	(Schltdl. & Cham.) Engl.	CBS, DEMP, NEC, DGS	10	LC
Cabombaceae	*Cabomba haynesii*	Wiersema	NEC	2	
Cactaceae	*Acanthocereus tetragonus*	(L.) Hummelinck	NEC, DRS	2	LC
Cactaceae	*Opuntia engelmannii subsp. lindheimeri*	(Engelm.) U. Guzmán & Mandujano	CBS, DS, NEC, DGS, DRS	9	LC
Cactaceae	*Opuntia pumila*	Rose	NEC, PCP, DRS	5	
Cactaceae	*Pereskia grandiflora*	Pfeiff.	NEC, DGS, DS, DMSC	4	
Cactaceae	*Pereskiopsis aquosa*	(F.A.C.Weber) Britton & Rose	CBS, NEC, DGS, DRS	6	LC
Cactaceae	*Rhipsalis baccifera*	(J.S.Muell.) Stearn	CBS, DCS, DS, NEC, DGS, DGUS, MBND, DMSC	13	LC
Cactaceae	*Selenicereus spinulosus*	(DC.) Britton & Rose	CBS, NEC, DGS, PCP	6	LC
Cactaceae	*Selenicereus undatus*	(Haw.) D.R.Hunt	DGS, PCP, CIPD	3	DD
Cannabaceae	*Celtis iguanaea*	(Jacq.) Sarg.	CBS, DS	3	LC
Cannabaceae	*Trema micrantha*	(L.) Blume	CIPD	1	LC

Cannaceae	*Canna indica*	L.	DGS, CIPD	2	
Capparaceae	*Cleoserrata serrata*	(Jacq.) Iltis	DVS	1	
Capparaceae	*Crateva tapia*	L.	DS, DEMP	5	LC
Caricaceae	*Carica papaya*	L.	PCP, IPEC	2	DD
Caryophyllaceae	*Stellaria ovata*	Willd. ex Schltdl.	DCS, NEC	2	
Celastraceae	*Crossopetalum uragoga*	(Jacq.) Kuntze	DS, DVS, DGS, DGUS, CIPD	6	
Cleomaceae	*Cleome aculeata*	L.	DGS	1	
Commelinaceae	*Callisia fragrans*	(Lindl.) Woodson	DCS, DEMP, DNS	3	
Commelinaceae	*Callisia repens*	(Jacq.) L.	NEC, DGUS	3	
Commelinaceae	*Commelina erecta*	L.	DS, NEC, DGS, DGUS, CIPD	8	LC
Commelinaceae	*Tradescantia zanonia*	(L.) Sw.	DEMP	1	
Commelinaceae	*Tradescantia zebrina var. zebrina*	(L.) Sw.	CBS, NEC, CIPD	5	
Connaraceae	*Rourea glabra*	Kunth	DS, NEC, DMSC	7	
Convolvulaceae	*Cuscuta corymbosa*	Ruiz & Pav.	DS, NEC	4	
Convolvulaceae	*Ipomoea alba*	L.	DS, NEC, DGS, PCP	8	LC
Convolvulaceae	*Ipomoea batatas*	(L.) Lam.	CIPD	1	DD
Convolvulaceae	*Ipomoea carnea*	Jacq.	PCP	1	
Convolvulaceae	*Ipomoea dumosa*	(Benth.) L.O. Williams	DVS, PCP, MBND	3	
Convolvulaceae	*Merremia dissecta*	(Jacq.) Hallier f.	DBBO, DS	2	
Convolvulaceae	*Merremia umbellata*	(L.) Hallier f.	DEMP, NEC	4	
Convolvulaceae	*Operculina pinnatifida*	(Kunth) O'Donell	DS, NEC	4	
Costaceae	*Costus pulverulentus*	C.Presl	DS, NEC, DGS, DGUS	6	
Crassulaceae	*Kalanchoe pinnata*	(Lam.) Pers	CBS, DCS, DS, NEC, DGS, MBND, ENMD, CIPD	18	
Cucurbitaceae	*Cucurbita sp. 1*		CBS	1	
Cucurbitaceae	*Cucurbita sp. 2*		CBS	1	
Cucurbitaceae	*Ibervillea sp.*		DS	1	
Cucurbitaceae	*Lagenaria siceraria*	(Molina) Standl.	DGUS, DRS	2	
Cucurbitaceae	*Melothria pendula*	L.	NEC	2	
Cupressaceae	*Taxodium mucronatum*	Ten.	PCP, DGUS	2	
Cyperaceae	*Cyperus hermaphroditus*	(Jacq.) Standl.	CBS, DS, NEC, DGUS	5	
Cyperaceae	*Eleocharis elegans*	(Kunth) Roem. & Schult.	CBS	1	
Cyperaceae	*Rhynchospora radicans*	(Schltdl. & Cham.) H.Pfeiff.	NEC	1	

Cyperaceae	*Scleria gaertneri*	Raddi	DS, DGS, CIPD	3	LC
Cytinaceae	*Bdallophytum americanum*	(R.Br.) Eichler ex Solms	NEC	1	
Dioscoreaceae	*Dioscorea alata*	L.	DGUS	1	
Dioscoreaceae	*Dioscorea mexicana*	Scheidw.	PCP, MBND	2	
Dryopteridaceae	*Thelypteris puberula*	(Baker) C.V.Morton	NEC	1	
Ebenaceae	*Diospyros nigra*	(J.F.Gmel.) Perrier	CBS	1	
Euphorbiaceae	*Acalypha phleoides*	Cav.	DGS	2	
Euphorbiaceae	*Astraea lobata*	(L.) Klotzsch	DS	2	
Euphorbiaceae	*Bernardia dodecandra*	(Sessé ex Cav.) Govaerts	CBS, DGUS	2	
Euphorbiaceae	*Cnidoscolus multilobus*	(Pax) I.M.Johnst.	DS, DGS, DGUS, FIHS, IPEC	7	LC
Euphorbiaceae	*Croton ciliatoglandulifer*	Ortega	CBS, DS, DMSC	4	
Euphorbiaceae	*Croton cortesianus*	Kunth	DBBO, DS, DVS, NEC, DGS, DGUS, CIPD, IPEC	12	LC
Euphorbiaceae	*Croton draco*	Schltdl.	DS, DGS	3	LC
Euphorbiaceae	*Croton niveus*	Jacq.	DS, NEC, DGS	3	LC
Euphorbiaceae	*Croton reflexifolius*	Kunth	DBBO, DS, DGS	6	LC
Euphorbiaceae	*Croton soliman*	Cham. & Schltdl.	CBS, DS, DGS, DMSC	8	
Euphorbiaceae	*Dalechampia scandens*	L.	NEC	1	LC
Euphorbiaceae	*Euphorbia colletioides*	Benth.	DS, NEC	3	
Euphorbiaceae	*Euphorbia dioscoreoides*	Boiss.	DS, NEC	2	
Euphorbiaceae	*Euphorbia heterophylla*	L.	CBS, DCS, NEC, DGS, PCP, DRS	8	LC
Euphorbiaceae	*Euphorbia hirta*	L.	DS	1	
Euphorbiaceae	*Euphorbia hypericifolia*	L.	DS	3	
Euphorbiaceae	*Euphorbia lancifolia*	Schltdl.	NEC, DGS, PCP	6	
Euphorbiaceae	*Euphorbia pulcherrima*	Willd. ex Klotzsch	CBS, DGUS, IPEC	3	LC
Euphorbiaceae	*Euphorbia serpens*	Kunth	DS, DEMP, DGS, PCP, DRS, CIPD	7	
Euphorbiaceae	*Euphorbia tithymaloides*	L.	DS, PCP, IPEC	5	LC
Euphorbiaceae	*Garcia nutans*	Vahl ex Rohr	MBND	1	LC
Euphorbiaceae	*Jatropha curcas*	L.	DBBO, DS, NEC, DGS	4	LC
Euphorbiaceae	*Manihot esculenta*	Crantz	NEC, CIPD	2	
Euphorbiaceae	*Ricinus communis*	L.	DS, NEC, DGS, PCP, DRS	10	

Euphorbiaceae	*Tragia mexicana*	Müll.Arg.	CBS, DS, CIPD, DMSC	5	
Fabaceae	*Acaciella angustissima*	(Mill.) Britton & Rose	DS, NEC, DGS, CIPD, DMSC	8	
Fabaceae	*Ateleia gummifera*	(DC.) D.Dietr.	DS	1	EN
Fabaceae	*Bauhinia divaricata*	L.	CBS, DBBO, DS, NEC, DGUS	7	LC
Fabaceae	*Caesalpinia pulcherrima*	(L.) Sw.	MBND	1	LC
Fabaceae	*Calliandra houstoniana*	(Mill.) Standl.	CBS, NEC, DGS, DRS, CIPD, IPEC	8	LC
Fabaceae	*Canavalia villosa*	Benth.	DS	4	
Fabaceae	*Centrosema sagittatum*	(Willd.) L.Riley	CBS	2	
Fabaceae	*Centrosema virginianum*	(L.) Benth.	DS	1	
Fabaceae	*Crotalaria vitellina*	Ker. Gawl.	CBS	3	
Fabaceae	*Dalea scandens var. paucifolia*	(J.M.Coult.) Barneby	DBBO, DEMP, NEC, DGS, IPEC	7	
Fabaceae	*Desmodium affine*	Schltdl.	NEC	1	
Fabaceae	*Desmodium axillare*	(Sw.) DC.	DS	1	
Fabaceae	*Desmodium incanum*	DC.	DS, NEC, IPEC	4	
Fabaceae	*Desmodium sp.*		IPEC	1	
Fabaceae	*Diphysa americana*	(Mill.) M.Sousa	CBS, DBBO, DS, NEC, PCP, DGUS, CIPD	11	
Fabaceae	*Enterolobium cyclocarpum*	(Jacq.) Griseb.	DRS	2	
Fabaceae	*Erythrina americana*	Mill.	NEC, DNS, PCP, FIHS	4	
Fabaceae	*Erythrina standleyana*	Krukoff	CBS, NEC, DGS, PCP, FIHS	6	LC
Fabaceae	*Eysenhardtia polystachya*	(Ortega) Sarg.	CBS	1	LC
Fabaceae	*Guilandina bonduc*	L.	NEC, DGS	2	
Fabaceae	*Harpalyce arborescens*	A.Gray	DGS	1	LC
Fabaceae	*Indigofera suffruticosa*	Mill.	DBBO, DGS, DNS, MBND	6	
Fabaceae	*Leucaena pulverulenta*	(Schltdl.) Benth.	CBS, NEC	2	LC
Fabaceae	*Lysiloma acapulcense*	(Kunth) Benth.	CBS, DS, NEC, DGS, DNS, DGUS, MBND, ENMD	16	LC
Fabaceae	*Marina scopa*	Barneby	NEC	1	
Fabaceae	*Mimosa albida*	Willd.	IPEC	1	LC
Fabaceae	*Mimosa pigra*	L.	PCP, MBND	3	LC
Fabaceae	*Mucuna argyrophylla*	Standl.	CBS, DS, NEC, CIPD	6	
Fabaceae	*Nissolia fruticosa*	Jacq.	CBS, DS, NEC, IPEC	5	

Fabaceae	*Oxyrhynchus volubilis*	Brandegee	DVS	1	LC
Fabaceae	*Pachyrhizus erosus*	(L.) Urb.	DS	3	
Fabaceae	*Phaseolus vulgaris*	L.	DGS	1	LC
Fabaceae	*Piscidia piscipula*	(L.) Sarg.	CBS, DBBO, DS, NEC, FIHS	10	
Fabaceae	*Pithecellobium dulce*	(Roxb.) Benth.	NEC, DGS	5	LC
Fabaceae	*Prosopis laevigata*	(Willd.) M.C.Johnst.	CIPD	1	LC
Fabaceae	*Pseudalbizzia tomentosa*	(Micheli) E.J.M.Koenen & Duno	CBS, NEC	2	LC
Fabaceae	*Rhynchosia longeracemosa*	M.Martens & Galeotti	DS	1	
Fabaceae	*Schrankia sp.*		NEC	2	
Fabaceae	*Senna candolleana*	(Vogel) H.S.Irwin & Barneby	CBS, NEC	8	
Fabaceae	*Senna hirsuta*	(L.) H.S.Irwin & Barneby	NEC, DS, DMSC	3	
Fabaceae	*Senna occidentalis*	(L.) Link	DCS, NEC, PCP, CIPD	7	LC
Fabaceae	*Senna pendula*	(Willd.) H.S.Irwin & Barneby	DS, NEC, DGS, DRS	9	LC
Fabaceae	*Tamarindus indica*	L.	CBS, NEC, DNS, CIPD	6	LC
Fabaceae	*Vachelia cornigera*	(L.) Seigler & Ebinger	DCS, DEMP, DGS, PCP, CIPD, IPEC	9	
Fabaceae	*Vachelia farnesiana*	(L.) Wight & Arn.	NEC, DGS, DS, DRS, DMSC	7	
Gentianaceae	*Eustoma exaltatum*	(L.) Salisb.	NEC	1	
Heliconiaceae	*Heliconia schiedeana*	Klotzsch	DBBO	1	
Iridaceae	*Alophia drummondii*	(Graham) R.C.Foster	CIPD	1	
Iridaceae	*Eleutherine bulbosa*	(Mill.) Urb.	NEC, CIPD	2	
Lamiaceae	*Callicarpa acuminata*	Kunth	NEC, DGS, PCP, DGUS	4	LC
Lamiaceae	*Clerodendrum chinense*	(Osbeck) Mabb.	DS	1	LC
Lamiaceae	*Clinopodium brownei*	(Sw.) Kuntze	CBS, NEC, DGS, DS, DMSC	8	
Lamiaceae	*Hedeoma drummondii*	Benth.	CBS, NEC, DGS, PCP, DRS	13	
Lamiaceae	*Mentha sp.*		NEC, DGS, PCP	3	
Lamiaceae	*Mesosphaerum pectinatum*	(L.) Kuntze	DS	1	
Lamiaceae	*Ocimum basilicum*	L.	NEC, DNS	2	

Lamiaceae	*Ocimum campechianum*	Mill.	CBS, NEC, DGS, DNS	12	
Lamiaceae	*Ocimum carnosum*	(Spreng.) Link & Otto ex Benth.	NEC	1	
Lamiaceae	*Origanum sp.*		CBS	2	
Lamiaceae	*Salvia coccinea*	Buc'hoz ex Etl.	CBS, DVS, NEC, DNS, DGUS, CIPD	9	
Lamiaceae	*Scutellaria seleriana*	Loes.	CBS, NEC, DGS, DS, DMSC	6	
Lamiaceae	*Teucrium cubense*	Jacq.	DCS, DBBO, DS, NEC, DGS	8	
Lauraceae	*Persea americana*	Mill.	DS, DRS	3	LC
Loasaceae	*Mentzelia hispida*	Willd.	DS, MBND	2	
Loranthaceae	*Psittacanthus schiedeanus*	(Schltdl. & Cham.) G.Don	DS, NEC, DGS, PCP, IPEC	6	
Loranthaceae	*Struthanthus crassipes*	(Oliv.) Eichler	DGS	1	
Loranthaceae	*Struthanthus quercicola*	(Schltdl. & Cham.) D.Don	DCS, NEC	4	
Lygodiaceae	*Lygodium venustum*	Sw.	NEC, DGS, PCP, MBND, ENMD, CIPD	13	
Lythraceae	*Cuphea decandra*	Dryand.	DS	1	
Lythraceae	*Cuphea salicifolia*	Schltdl. & Cham.	NEC	1	
Lythraceae	*Heimia salicifolia*	(Kunth) Link	CBS, NEC	3	
Lythraceae	*Lythrum gracile*	Benth.	CIPD	1	
Lythraceae	*Punica granatum*	L.	NEC	1	LC
Malpighiaceae	*Malpighia glabra*	L.	NEC, DRS	5	LC
Malvaceae	*Abelmoschus moschatus*	Medik.	DCS, NEC, DGS, DS, DRS, DMSC	12	
Malvaceae	*Abutilon hypoleucum*	A.Gray	NEC, DGS	3	
Malvaceae	*Allosidastrum pyramidatum*	(Desp. ex Cav.) Krapov., Fryxell & Bates	PCP	1	
Malvaceae	*Anoda cristata*	(L.) Schltdl.	DS, NEC, ENMD, CIPD	6	
Malvaceae	*Ceiba pentandra*	(L.) Gaertn.	DS, NEC	2	LC
Malvaceae	*Corchorus siliquosus*	L.	CBS, DS, DVS, NEC, DGS, PCP, DGUS, IPEC	9	
Malvaceae	*Gossypium hirsutum*	L.	DCS, NEC, DGS, DS, PCP, DGUS, DRS, CIPD, DMSC	12	VU
Malvaceae	*Guazuma ulmifolia*	Lam.	DS, NEC, DGS, PCP, DRS, CIPD, IPEC	9	LC

Malvaceae	*Heliocarpus donnellsmithii*	Rose	CBS, DCS, NEC, DGS, PCP, DRS, CIPD, IPEC	9	
Malvaceae	*Herissantia crispa*	(L.) Brizicky	DGUS	1	
Malvaceae	*Hibiscus lavateroides*	Moric.	PCP, DGUS	2	
Malvaceae	*Hibiscus rosa-sinensis*	L.	PCP	1	
Malvaceae	*Malachra capitata*	(L.) L.	NEC	1	
Malvaceae	*Malvastrum americanum*	(L.) Torr.	DS, NEC, DGS, IPEC	5	
Malvaceae	*Malvastrum coromandelianum*	(L.) Garcke	DS, CIPD	2	
Malvaceae	*Malvaviscus arboreus*	Dill. ex Cav.	DCS, NEC, DGS, DS, PCP, DGUS, DRS, DMSC	14	LC
Malvaceae	*Melochia nodiflora*	Sw.	NEC, PCP, DGUS, CIPD	7	
Malvaceae	*Melochia pyramidata*	L.	NEC	1	LC
Malvaceae	*Pavonia schiedeana*	Steud.	CBS, DS, NEC, DGS, PCP, DGUS, DRS, CIPD, DMSC	17	
Malvaceae	*Phymosia umbellata*	(Cav.) Kearney	CIPD	1	LC
Malvaceae	*Pseudobombax ellipticum*	(Kunth) Dugand	CBS, NEC, DGS, DNS, DGUS, MBND, CIPD	10	LC
Malvaceae	*Sida acuta*	Burm.f.	CBS, DS, DGS, IPEC	8	
Malvaceae	*Triumfetta semitriloba*	Jacq.	NEC, DGS, PCP, DGUS, DRS, CIPD	10	LC
Marantaceae	*Maranta arundinacea*	L.	CBS, NEC, DGS, DGUS, CIPD	8	
Marantaceae	*Maranta gibba*	Sm.	NEC, CIPD	2	
Martyniaceae	*Martynia annua*	L.	DRS, CIPD	2	
Melanthiaceae	*Schoenocaulon officinale*	(Schltdl. & Cham.) A.Gray	DS	2	
Melastomataceae	*Miconia petiolaris*	(Schltdl.) Michelang.	DCS, NEC	2	
Meliaceae	*Cedrela odorata*	L.	CBS, DBBO, NEC, DRS, CIPD, IPEC	12	PR/VU
Meliaceae	*Trichilia havanensis*	Jacq.	CBS, DBBO, NEC, DRS	8	LC
Menispermaceae	*Cissampelos pareira*	L.	CBS, NEC	4	
Menispermaceae	*Cocculus diversifolius*	DC.	CBS, DCS, NEC, DGS	4	
Montiaceae	*Talinum fruticosum*	(L.) Juss.	DGS, DGUS, CIPD	3	
Montiaceae	*Talinum paniculatum*	(Jacq.) Gaertn.	CBS, DGS	2	
Moraceae	*Brosimum alicastrum*	Sw.	DGS	1	LC

Moraceae	*Castilla elastica*	Cerv.	NEC, DGS, DS, DRS, IPEC, DMSC	11	LC
Moraceae	*Dorstenia contrajerva*	L.	CBS, NEC, DGS, DS, DGUS, DMSC	12	
Moraceae	*Ficus americana*	Aubl.	DBBO, NEC, DS, IPEC, DMSC	9	LC
Moraceae	*Ficus maxima*	Mill.	NEC, DGS, IPEC	3	LC
Moraceae	*Ficus obtusifolia*	Kunth	NEC, DGS, DS, IPEC, DMSC	5	LC
Moraceae	*Ficus pertusa*	L.f.	DS, NEC, DRS, CIPD, IPEC, DMSC	10	LC
Moraceae	*Maclura tinctoria*	(L.) D.Don ex Steud.	CBS, DS, NEC, DGS, DGUS, FIHS, MBND, DRS, DMSC	15	LC
Moraceae	*Trophis racemosa*	(L.) Urb.	CBS, DGS	2	LC
Muntingiaceae	*Muntingia calabura*	L.	DGUS	1	
Musaceae	*Musa* x *paradisiaca*	L.	DS, CIPD	2	
Myrtaceae	*Eugenia capuli*	(Schltdl. & Cham.) Hook. & Arn.	NEC, DGS, DRS	5	LC
Myrtaceae	*Eugenia karwinskyana*	O.Berg	CBS	1	LC
Myrtaceae	*Eugenia oerstediana*	O.Berg	DGS	1	LC
Myrtaceae	*Psidium guajava*	L.	NEC, DGS, DGUS, DRS, CIPD	10	LC
Myrtaceae	*Psidium guineense*	Sw.	DGS	2	LC
Nyctaginaceae	*Bougainvillea spectabilis*	Willd.	DBBO	1	
Nyctaginaceae	*Mirabilis jalapa*	L.	CBS, NEC, PCP, DRS, CIPD	6	
Nyctaginaceae	*Neea psychotrioides*	Donn.Sm.	NEC, CIPD	3	LC
Nyctaginaceae	*Pisonia aculeata*	L.	DS, NEC	3	LC
Nymphaeaceae	*Nymphaea ampla*	(Salisb.) DC.	NEC, PCP	2	
Oleaceae	*Jasminum sp.*		NEC, DRS	3	
Onagraceae	*Oenothera kunthiana*	(Spach) Munz	NEC, DGS	2	
Onagraceae	*Oenothera rosea*	L'Hér. ex Aiton	IPEC	1	
Onagraceae	*Oenothera tetraptera*	Cav.	DCS, NEC, DGS, DNS	7	
Orchidaceae	*Epidendrum difforme*	Jacq.	DCS, NEC	4	
Orchidaceae	*Isochilus linearis*	(Jacq.) R.Br.	CIPD	1	
Orchidaceae	*Kionophyton seminuda*	(Schltr.) Garay	PCP	2	
Orchidaceae	*Trichocentrum carthagenense*	(Jacq.) M.W.Chase & N.H.Williams	NEC	2	
Oxalidaceae	*Oxalis dillenii*	Jacq.	CBS	1	
Oxalidaceae	*Oxalis latifolia*	Kunth	DS, DGS	2	

Pandanaceae	*Pandanus odorifer*	(Forssk.) Kuntze	DS, IPEC, DMSC	3	LC
Papaveraceae	*Argemone mexicana*	L.	DVS	1	
Papaveraceae	*Bocconia frutescens*	L.	DGS	1	LC
Passifloraceae	*Passiflora coriacea*	Juss.	DS, DEMP, NEC, CIPD	7	
Passifloraceae	*Passiflora foetida*	L.	NEC, DGS, PCP, DGUS, CIPD	7	
Petiveriaceae	*Petiveria alliacea*	L.	CBS, NEC, DGS, IPEC	10	
Petiveriaceae	*Rivina humilis*	L.	CBS, DS, NEC, CIPD, IPEC	10	
Phyllanthaceae	*Phyllanthus adenodiscus*	Müll.Arg.	DS, NEC, CIPD	6	
Phyllanthaceae	*Phyllanthus niruri*	L.	CBS, DS, NEC, MBND	9	
Picramniaceae	*Picramnia antidesma*	Sw.	DS	2	
Pinaceae	*Pinus teocote*	Schied. ex Schltdl. & Cham.	CBS, NEC, DGS	7	LC
Piperaceae	*Peperomia glabella*	(Sw.) A.Dietr.	IPEC	2	
Piperaceae	*Peperomia liebmannii*	C. DC.	CBS	1	
Piperaceae	*Peperomia rotundifolia*	(L.) Kunth	NEC	1	
Piperaceae	*Peperomia sp.*		CBS, NEC	2	
Piperaceae	*Piper amalago*	L.	CBS, DS, NEC, PCP, DRS, DMSC	16	LC
Piperaceae	*Piper sanctum*	(Miq.) Schltdl. ex C.DC.	NEC, DGS	4	LC
Piperaceae	*Piper schiedeanum*	Steud.	CIPD	1	LC
Piperaceae	*Piper umbellatum*	L.	DS, NEC, DGS, PCP, DRS, CIPD, IPEC, DMSC	17	
Plantaginaceae	*Mecardonia procumbens*	(Mill.) Small	CBS, NEC, MBND, ENMD, CIPD	7	
Plantaginaceae	*Russelia equisetiformis*	Schltdl. & Cham.	CBS, NEC	2	
Plantaginaceae	*Russelia sarmentosa*	Jacq.	CBS, DGUS	2	
Plumbaginaceae	*Plumbago zeylanica*	L.	DCS, IPEC	3	
Poaceae	*Arundinella berteroniana*	(Schult.) Hitchc. & Chase	CBS	1	
Poaceae	*Arundo donax*	L.	CBS, DS, CIPD, DMSC	5	LC
Poaceae	*Cenchrus echinatus*	L.	CBS, NEC, DGS, DGUS, CIPD	10	LC
Poaceae	*Cenchrus preslii*	(Kunth) ined.	CBS, NEC, CIPD	4	
Poaceae	*Cymbopogon citratus*	(DC.) Stapf	NEC, DGS, DGUS	3	
Poaceae	*Hilaria ciliata*	(Scribn.) Sohns	DGS	1	
Poaceae	*Imperata brasiliensis*	Trin.	DGS, DGUS, CIPD	4	

Poaceae	*Lasiacis ruscifolia*	(Kunth) Hitchc. ex Chase	NEC, DGS, DS, DGUS, DMSC	5	
Poaceae	*Lithachne pauciflora*	(Sw.) P.Beauv.	NEC, PCP	2	
Poaceae	*Panicum virgatum*	L.	DRS	1	
Poaceae	*Paspalum paniculatum*	L.	NEC	1	LC
Poaceae	*Paspalum sp.*		DS	1	
Poaceae	*Saccharum officinarum*	L.	CIPD	1	
Poaceae	*Setaria liebmannii*	E.Fourn.	DGS	1	
Poaceae	*Zea mays*	L.	CBS, DS, NEC, DGS, DGUS, DRS, ENMD	10	
Polygalaceae	*Polygala nitida var. lithophila*	(S.F. Blake) T. Wendt	DS, DGUS, FIHS	4	
Polygalaceae	*Securidaca diversifolia*	(L.) S.F.Blake	DS, DGS, DMSC	4	LC
Polygonaceae	*Polygonum mexicanum*	Small	NEC	1	
Polypodiaceae	*Campyloneurum phyllitidis*	(L.) C. Presl	CBS, DCS, DEMP, NEC	6	A
Polypodiaceae	*Microgramma nitida*	(J.Sm.) A.R.Sm.	DCS, DS, IPEC, DMSC	4	
Polypodiaceae	*Pecluma plumula*	(Humb. & Bonpl. ex Willd.) M.G. Price	NEC	1	
Polypodiaceae	*Phlebodium aureum*	(L.) J. Sm.	NEC, PCP, DRS	4	
Polypodiaceae	*Phlebodium decumanum*	(Willd.) J. Sm.	CBS, NEC, DGS, PCP, DRS, CIPD, IPEC	8	
Polypodiaceae	*Pleopeltis polypodioides*	(L.) E.G.Andrews & Windham	CBS, NEC	3	
Portulacaceae	*Portulaca oleracea*	L.	NEC, DGS	2	LC
Portulacaceae	*Portulaca pilosa*	L.	DS, NEC, DRS, CIPD	4	
Primulaceae	*Ardisia escallonioides*	Schltdl. & Cham.	NEC, DGS, CIPD	5	LC
Primulaceae	*Ardisia nigrescens*	Oerst.	NEC	1	LC
Primulaceae	*Parathesis serrulata*	(Sw.) Mez	NEC, DGS	2	
Pteridaceae	*Adiantum poiretii*	Wikstr.	CBS, DCS, NEC	6	
Pteridaceae	*Adiantum tenerum*	Sw.	NEC	1	
Pteridaceae	*Adiantum tricholepis*	Fée	NEC	1	
Pteridaceae	*Hemionitis radiata*	(L.) Christenh.	CBS	1	
Ranunculaceae	*Clematis grossa*	Benth.	DS, DGS, DRS	4	
Rhamnaceae	*Colubrina greggii*	S.Watson	DS, DRS, DMSC	5	LC
Rhamnaceae	*Gouania polygama*	(Jacq.) Urb.	CBS, DCS, DS	7	
Rhamnaceae	*Karwinskia humboldtiana*	(Schult.) Zucc.	CBS	1	
Rosaceae	*Prunus samydoides*	Schltdl.	NEC, DGS, CIPD	3	LC

Rosaceae	*Rosa sp.*		NEC, CIPD	4	
Rubiaceae	*Chiococca alba*	(L.) Hitchc.	DBBO, NEC, DS, DMSC	6	LC
Rubiaceae	*Coffea arabica*	L.	IPEC	1	EN
Rubiaceae	*Hamelia patens*	Jacq.	DBBO, DS, NEC, DGS, DGUS, CIPD, IPEC	16	LC
Rubiaceae	*Psychotria erythrocarpa*	Schltdl.	DS, NEC, DGS, CIPD	8	
Rubiaceae	*Psychotria nervosa*	Sw.	CBS, DS, NEC, DRS, CIPD	10	LC
Rubiaceae	*Psychotria sp.*		DS	1	
Rubiaceae	*Randia laetevirens*	Standl.	NEC, DGS, IPEC	3	LC
Rutaceae	*Citrus aurantiifolia*	(Christm.) Swingle	CBS, DS, NEC, DGS, PCP, DRS, CIPD	13	
Rutaceae	*Citrus* x *aurantium*	L.	CBS, DCS, DS, NEC, DGS, DNS, MBND, DRS, CIPD	16	
Rutaceae	*Citrus limon*	(L.) Osbeck	DRS	1	
Rutaceae	*Decatropis bicolor*	(Zucc.) Radlk.	CBS, NEC, DGS	5	
Rutaceae	*Esenbeckia berlandieri*	Baill.	NEC	2	EN
Rutaceae	*Zanthoxylum fagara*	(L.) Sarg.	NEC, DGS, DS, PCP, CIPD, DMSC	13	LC
Salicaceae	*Casearia aculeata*	Jacq.	DS, DRS	2	
Salicaceae	*Populus mexicana*	Sarg.	NEC, MBND	4	NT
Salicaceae	*Salix humboldtiana*	Willd.	NEC	2	LC
Salicaceae	*Xylosma flexuosa*	(Kunth) Hemsl.	DGS, DS, DRS, CIPD, IPEC, DMSC	11	LC
Salicaceae	*Casearia laetioides*	(A.Rich.) Warb.	DVS, NEC	2	
Santalaceae	*Phoradendron quadrangulare*	(Kunth) Griseb.	CBS, NEC	3	
Sapindaceae	*Exothea copalillo*	(Schltdl.) Radlk.	NEC, DNS, MBND	3	EN
Sapindaceae	*Paullinia tomentosa*	Jacq.	DS, NEC, DGS, DGUS, FIHS, CIPD	9	
Sapindaceae	*Sapindus saponaria*	L.	CBS, NEC	3	LC
Sapindaceae	*Serjania rachiptera*	Radlk.	DGS, CIPD	4	
Sapindaceae	*Urvillea ulmacea*	Kunth	NEC	1	
Sapotaceae	*Manilkara zapota*	(L.) P.Royen	CIPD	1	LC
Scrophulariaceae	*Buddleja americana*	L.	NEC, CIPD, IPEC	4	LC
Scrophulariaceae	*Capraria mexicana*	Moric. ex Benth.	DGS, DNS	2	
Scrophulariaceae	*Capraria saxifragifolia*	Schltdl. & Cham.	DCS, DBBO	2	
Selaginellaceae	*Selaginella martensii*	Spring	CBS, NEC, MBND, IPEC	5	

Selaginellaceae	*Selaginella sp.*		NEC	1	
Smilacaceae	*Smilax domingensis*	Willd.	NEC, DGS, DGUS, CIPD, IPEC	5	
Smilacaceae	*Smilax sp.*		DGUS	1	
Solanaceae	*Brugmansia x candida*	Pers.	CBS, DS, MBND, IPEC	5	
Solanaceae	*Capsicum annuum*	L.	CIPD	1	LC
Solanaceae	*Capsicum annuum var. glabriusculum*	(Dunal) Heiser & Pickersgill	CBS, DS, IPEC	8	
Solanaceae	*Cestrum dumetorum*	Schltdl.	CBS, DS, NEC, DGS, DNS, PCP, DGUS, DRS, CIPD, IPEC, DMSC	21	
Solanaceae	*Cestrum nocturnum*	L.	CBS, CIPD	3	LC
Solanaceae	*Datura stramonium*	L.	CBS, DS, NEC, DGUS, MBND, CIPD	7	
Solanaceae	*Nicotiana tabacum*	L.	DCS, NEC, DGS, IPEC	4	
Solanaceae	*Physalis gracilis*	Miers	CIPD	1	LC
Solanaceae	*Physalis melanocystis*	(B.L.Rob.) Bitter	DEMP, NEC	3	LC
Solanaceae	*Physalis solanaceus*	(Schltdl.) Axelius	NEC, CIPD	2	
Solanaceae	*Physalis virginiana*	Mill.	DS	2	LC
Solanaceae	*Physalis viscosa*	L.	NEC, DGS, CIPD	3	
Solanaceae	*Solandra maxima*	(Moc. & Sessé ex Dunal) P.S.Green	DVS, NEC	2	
Solanaceae	*Solanum americanum*	Mill.	DS, DEMP, CIPD, IPEC	9	
Solanaceae	*Solanum aphyodendron*	S.Knapp	DS, NEC	3	
Solanaceae	*Solanum diphyllum*	L.	CBS, DS, DEMP, NEC, DRS	22	
Solanaceae	*Solanum lanceifolium*	Jacq.	PCP	1	
Solanaceae	*Solanum torvum*	Sw.	DS, NEC, DGS, MBND, IPEC	9	
Solanaceae	*Solanum umbellatum*	Mill.	CBS, NEC, DS, DMSC	5	
Tectariaceae	*Tectaria heracleifolia*	(Willd.) Underw.	CBS, DCS, DBBO, NEC, DGS, PCP, CIPD	12	
Urticaceae	*Cecropia obtusifolia*	Bertol.	DS	1	LC
Urticaceae	*Pilea imparifolia*	Wedd.	NEC	1	
Urticaceae	*Pilea microphylla*	(L.) Liebm.	CBS, DS, NEC, DGS, CIPD	11	

Urticaceae	*Pilea pubescens*	Liebm.	CBS, DVS, NEC, PCP, DRS, CIPD	8	
Urticaceae	*Pouzolzia occidentalis*	(Liebm.) Wedd.	CBS, DCS, NEC, DS, PCP, DGUS, DRS, IPEC, DMSC	17	
Urticaceae	*Urera caracasana*	(Jacq.) Gaudich. ex Griseb.	DVS, CIPD	2	LC
Urticaceae	*Urera sp.*		DCS, NEC, DS, DRS, CIPD, DMSC	16	
Urticaceae	*Urtica chamaedryoides*	Pursh	DS, DMSC	3	
Verbenaceae	*Citharexylum berlandieri*	S.Watson	DS, DMSC	4	LC
Verbenaceae	*Lantana achyranthifolia*	Desf.	NEC, DGS	3	
Verbenaceae	*Lantana camara*	L.	CBS, DS, DEMP, NEC, DGS, DGUS, CIPD, IPEC	11	
Verbenaceae	*Lantana involucrata*	L.	NEC	1	LC
Verbenaceae	*Lippia myriocephala*	Schltdl. & Cham.	DS, NEC, DGS, DGUS, CIPD	8	LC
Verbenaceae	*Lippia origanoides*	Kunth	DGS	1	
Verbenaceae	*Petrea volubilis*	L.	NEC, DGS, PCP, DGUS, MBND, DRS, CIPD	9	
Verbenaceae	*Priva lappulacea*	(L.) Pers.	DGS	2	
Verbenaceae	*Tamonea curassavica*	(L.) Pers.	DGS, DGUS	3	
Verbenaceae	*Verbena delticola*	Small ex Perry	CBS, DS, NEC, PCP	6	
Vitaceae	*Cissus microcarpa*	Vahl	CIPD	1	
Vitaceae	*Cissus verticillata*	(L.) Nicolson & C.E.Jarvis	DS, IPEC	5	LC
Zamiaceae	*Ceratozamia mexicana*	Brongn.	DGUS	1	A/CR
Zamiaceae	*Zamia sp.*		CBS, NEC, DGUS, ENMD, CIPD, IPEC	7	
Zingiberaceae	*Curcuma longa*	L.	DS, DRS, DMSC	3	DD
Zingiberaceae	*Zingiber officinale*	Roscoe	NEC, DGS, DS, DRS, DMSC	5	DD
