# Supplementary material for: Diversity and distribution of the Huastec Mayan medicinal plants: hotspots for bioprospecting and conservation
Source: Biodivers Data J. 2025 Dec 8;13:e170091. doi: 10.3897/BDJ.13.e170091 (PMC12706493; doi:10.3897/BDJ.13.e170091)
Supplement: Supplementary material 2 — Table S2 [file bdj-13-e170091-s002.pdf]

Table S2. Data base of the plant species used in the Huastec Mayan traditional medicine, taxonomy, common names, origin, growth forms, habitat, risk status according to NOM-059 and IUCN, classes of disease they treat according to ICD-11 of WHO (2024) and the CBS category.

Family	Scientific name	Tenek name	Other common names	Origin	Growth form	Habitat	UICN category	NOM-059 category	ICD-11 Classes
Acanthaceae	*Aphelandra* sp.	muuw te'	not registered	exotic	shrub	homegarden	no risk	no risk	CBS
Acanthaceae	*Dianthera pectoralis* (Jacq.) J.F.Gmel.	pithomlaab ts' ohool	not registered	native	herb	homegarden	no risk	no risk	CBS, NEC, DGS
Acanthaceae	*Dicliptera sexangularis* (L.) Juss.	wayelom ts' ohool	not registered	native	herb	wild	no risk	no risk	CBS, NEC, PCP
Acanthaceae	*Elytraria bromoides* Oerst.	xutsun bat' aw	not registered	native	herb	wild	no risk	no risk	DEMP
Acanthaceae	*Justicia brandegeeana* Wassh. & L.B.Sm.	tsaakuy elul	not registered	native	herb	homegarden, wild	no risk	no risk	CBS, NEC, DGS, PCP, CIPD, NE
Acanthaceae	*Justicia fulvicoma* Schltdl. & Cham.	tsaakuy elul	not registered	native	herb	wild	no risk	no risk	NEC, DGUS, CIPD
Acanthaceae	*Justicia spicigera* Schltdl.	muh, muu	mojuite, muicle	native	herb	homegarden, wild	no risk	no risk	CBS, DBBO, NEC, PCP, DGUS, DRS, CIPD
Acanthaceae	*Odontonema callistachyum* (Schltdl. & Cham.) Kuntze	t'a'lom ts'ohool	not registered	native	herb	wild	no risk	no risk	NEC, PCP
Acanthaceae	*Odontonema tubaeforme* (Bertol.) Kuntze	k'alul ts'ohool	not registered	native	herb	wild	no risk	no risk	PCP
Acanthaceae	*Ruellia ciliatiflora* Hook.	eem muuw	not registered	native	herb	homegarden, milpa, wild	no risk	no risk	NEC, IPEC
Acanthaceae	*Ruellia simplex* C.Wright	tsab k'a'um	not registered	native	herb	wild	no risk	no risk	CBS, NEC, CIPD
Acanthaceae	*Thunbergia alata* Bojer ex Sims	tsaayleel ts'ohool, tsaayleel ts'ojol	hierba del susto	exotic	climbing	wild	no risk	no risk	NEC
Acanthaceae	*Thunbergia fragrans* Roxb.	ik ts'ohool	not registered	exotic	climbing	disturbed	no risk	no risk	NEC
Agdestidaceae	*Agdestis clematidea* Moc. & Sessé ex DC.	tuuwi'	sueldo consueldo	native	climbing	homegarden, milpa, wild	no risk	no risk	CBS, DS, IPEC. DMSC

Alstroemeriaceae	*Bomarea edulis* (Tussac) Herb.	San Migeel wits	flor de San Miguel	native	climbing	homegarden, milpa, sugarcane cultivation	no risk	no risk	DS, DGS, MBND, ENMD
Amaranthaceae	*Achyranthes aspera* L.	ts'aah pathaam	not registered	exotic	herb	wild	no risk	no risk	CIPD
Amaranthaceae	*Alternanthera philoxeroides* (Mart.) Griseb.	wiichab ts'ohool	not registered	exotic	herb	wild	no risk	no risk	DS, NEC, CIPD
Amaranthaceae	*Alternanthera sessilis* (L.) R.Br. ex DC.	tiyankix pepetal	not registered	native	herb	not registered	least concern (LC)	no risk	NEC, CIPD
Amaranthaceae	*Amaranthus caudatus* L.	chith	alegría, chía	exotic	herb	homegarden, milpa	no risk	no risk	NEC, DGS
Amaranthaceae	*Amaranthus hybridus* L.	chithal tooro	quelite	native	herb	homegarden, milpa, wild	no risk	no risk	DS
Amaranthaceae	*Dysphania ambrosioides* (L.) Mosyakin & Clemants	tihtsan, tij-tzan, titchan	apazote, epazote	native	herb	homegarden	no risk	no risk	NEC, DGS, PCP, CIPD, IPEC
Amaranthaceae	*Gomphrena globosa* L.	t' oyol	not registered	exotic	herb	homegarden	no risk	no risk	CIPD
Amaryllidaceae	*Allium longifolium* (Kunth) Spreng.	huum nakat, huun nakat	cebollín	native	herb	homegarden, wild	no risk	no risk	DS, NEC, IPEC, DMSC
Amaryllidaceae	*Allium sativum* L.	aaxux	ajo	exotic	herb	homegarden	no risk	no risk	CBS, NEC, DGS, DS, CIPD, DMSC
Amaryllidaceae	*Hymenocallis* sp.	lakuum	lirio	native	herb	homegarden, wild	no risk	no risk	CIPD
Anacardiaceae	*Spondias purpurea* L.	ten, teen	ciruelo	native	tree	homegarden	least concern (LC)	no risk	DS, DGS, CIPD
Anemiaceae	*Anemia adiantifolia* (L.) Sw.	huniil akam pich	not registered	native	herb	wild	no risk	no risk	NEC
Anemiaceae	*Anemia mexicana* Klotzsch	boo'waat ts'ohool	not registered	native	herb	Wild	no risk	no risk	NEC
Annonaceae	*Annona globiflora* Schltdl.	anchich, anchuch, aanchuuch	anonilla, chirimoya	endemic	tree	homegarden, milpa, wild	no risk	no risk	CBS, DS, NEC, IPEC

Annonaceae	*Annona reticulata* L.	cúcay, cúquey, kukay	anona	native	tree	homegarden, wild	least concern (LC)	no risk	CBS, NEC, DGUS, CIPD, NE
Apiaceae	*Coriandrum sativum* L.	kulaantun	cilantro, cilantro hoja pequeña	exotic	herb	homegarden	no risk	no risk	DGS
Apiaceae	*Cyclospermum leptophyllum* (Pers.) Sprague	kulantoil an t'ot	not registered	native	herb	homegarden	no risk	no risk	CIPD
Apiaceae	*Eryngium* sp.	tsakam bathuch	not registered	native	herb	wild	no risk	no risk	CIPD
Apocynaceae	*Asclepias curassavica* L.	ponchihuitz, punchihuitz, puunchiix wits	not registered	native	herb	homegarden, milpa, wild	no risk	no risk	CBS, DS, DGS, CIPD
Apocynaceae	*Cascabela thevetia* (L.) Lippold	ts' een aanchuuch, tzenantzuch	guayapol, hierba de San Pedro, narciso, palo de San Antonio, San Diego, San Nicolás	native	shrub	homegarden, wild	least concern (LC)	no risk	DCS, DS, DGS
Apocynaceae	*Echites panduratus* A.DC.	t' obts' i'	bajo de wey, lengua de vaca	native	climbing	homegarden, wild	no risk	no risk	NEC, DGS, ENMD
Apocynaceae	*Echites tuxtlensis* Standl.	tsank' ub ts' aah	not registered	native	climbing	homegarden, milpa, wild	no risk	no risk	NEC, DGS, DRS, IPEC
Apocynaceae	*Gonolobus niger* (Cav.) Schult.	oy, ooy	cahuayote	native	shrub	homegarden, wild	no risk	no risk	IPEC
Apocynaceae	*Pentalinon andrieuxii* (Müll.Arg.) B.F.Hansen & Wunderlin	look' ts'aah	contrayerba	native	shrub	homegarden, wild	no risk	no risk	DS, CIPD, IPEC
Apocynaceae	*Plumeria rubra* L.	uculhitz, ukul wits, mamaal peet	flor de mayo	native	shrub	homegarden	least concern (LC)	no risk	CBS, DS
Apocynaceae	*Rauvolfia tetraphylla* L.	itsaan an k' ak' al ilaal	ajillo	native	herb	homegarden, wild	least concern (LC)	no risk	NEC

Apocynaceae	*Tabernaemontana alba* Mill.	ábat, tábat, t' abat', t'abat'te'	cajón de gato, cojón de gato, huevo de gato	native	tree	homegarden, milpa, wild	least concern (LC)	no risk	DS, DGS, IPEC
Apocynaceae	*Ruehssia macrophylla* (Humb. & Bonpl. ex Schult.) H.Karst.	tan ooy	not registered	exotic	climbing	wild	no risk	no risk	IPEC
Araceae	*Syngonium podophyllum* Schott	cuath, kwaath	not registered	native	herb	wild	no risk	no risk	CIPD
Araceae	*Xanthosoma robustum* Schott	lu, lun, ts'ikiy luum, tzailu	chamol, malanga, ñampí, rejalgar, rejalgar	native	herb	wild	no risk	no risk	DS
Araceae	*Xanthosoma sagittifolium* (L.) Schott	kwithom luum, luum	not registered	native	herb	homegarden	no risk	no risk	DS, NEC
Araliaceae	*Dendropanax arboreus* (L.) Decne. & Planch.	multe, multe'	mulumento, nixtamalillo, palo santo, vidrioso, zapotillo	native	tree	wild	no risk	no risk	CBS, NEC, DS, DRS, DMSC
Arecaceae	*Acrocomia aculeata* (Jacq.) Lodd. ex Mart.	map, maap	corozo, coyol, palma de coyol	native	tree	homegarden, milpa, wild	least concern (LC)	no risk	DRS, CIPD
Arecaceae	*Chamaedorea elegans* Mart.	weew bat' aw	corozillo, palmilla	native	herb	wild	no risk	no risk	MBND, CIPD
Arecaceae	*Sabal mexicana* Mart.	ápatz, otoomal	palma, palma de mícheros, palmito, palma real	native	tree	homegarden, milpa, sugarcane cultivation, wild	least concern (LC)	no risk	CBS, DS, NEC, DGS, CIPD
Aristolochiaceae	*Aristolochia littoralis* Parodi	itsaan an ohob ilaal	not registered	exotic	climbing	wild	no risk	no risk	DGS, DRS
Aristolochiaceae	*Aristolochia orbicularis* Duch.	ohob ilaal	not registered	endemi c	herb	homegarden, milpa, wild	no risk	no risk	DS, DRS, DMSC
Aristolochiaceae	*Aristolochia* sp.	ehtiil ohob ilaal	huaco	native	herb	not registered	no risk	no risk	DS, NEC
Asparagaceae	*Agave* sp.	huey, tsi'iim, tzihim	maguey, maguey pulquero	native	rosetophil a	homegarden, wild	no risk	no risk	CBS, NEC, PCP, DGUS,

									DRS, CIPD, IPEC
Asparagaceae	*Agave variegata* Jacobi	pulik k' oyol	sebadiya	native	herb	wild	no risk	no risk	DS, NEC
Asparagaceae	*Beschorneria* sp.	tsakam tsi'iim	not registered	native	rosetophil a	homegarden	no risk	no risk	NEC, IPEC
Asparagaceae	*Echeandia reflexa* (Cav.) Rose	eem ts' ohool	not registered	native	herb	homegarden, milpa, wild	no risk	no risk	DS, NEC, DGS, DGUS
Asparagaceae	*Yucca gigantea* Lem.	cóyol, tsamnek k'oyol, tzamuc-cóyol	coyol de chaneque, izote, telicso	native	tree	homegarden, wild	no risk	no risk	CBS, DEMP, PCP
Asphodelaceae	*Aloe vera* (L.) Burm.f.	xabila	sábila	exotic	herb	homegarden	no risk	no risk	CBS, DS, CIPD, IPEC
Aspleniaceae	*Asplenium pumilum* Sw.	xapachik ts'ohool	not registered	native	herb	wild	no risk	no risk	DCS, ENMD
Asteraceae	Aldama dentata La Llave ex La Llave	k'an wits	not registered	native	herb	wild	no risk	no risk	DS, DRS
Asteraceae	*Anthemis* sp.	manzaniya	manzanilla	exotic	herb	not registered	no risk	no risk	CBS, DGS
Asteraceae	*Artemisia ludoviciana* Nutt.	teen wits	estafiate	native	herb	homegarden	no risk	no risk	DS, DEMP, NEC, CIPD, IPEC
Asteraceae	*Bidens pilosa* L.	kelem, quélem	not registered	native	herb	homegarden, milpa, wild	no risk	no risk	DS, NEC, DGS, DRS
Asteraceae	*Bidens reptans* G.Don	kelem ts'aah	not registered	native	herb	milpa, wild	no risk	no risk	DS, NEC
Asteraceae	*Brickellia diffusa* (Vahl) A.Gray	komino ts'ohool	not registered	native	herb	wild	no risk	no risk	DVS
Asteraceae	*Calea ternifolia* Kunth	tsuleek' ethem	hierba de piojo	native	herb	homegarden, wild	no risk	no risk	DS, NEC, DRS, CIPD
Asteraceae	*Carduus* sp.	ohob ilaal	not registered	exotic	herb	homegarden	no risk	no risk	NEC, DS, DRS, DMSC
Asteraceae	*Chaptalia nutans* (L.) Polák	tsakam yehtsel	not registered	native	herb	not registered	no risk	no risk	NEC, CIPD
Asteraceae	*Chromolaena collina* (DC.) R.M.King & H.Rob.	ehek witsiim	not registered	native	shrub	homegarden, milpa, wild	least concern (LC)	no risk	CBS, DBBO, DS, NEC, DGS, PCP

Asteraceae	*Chromolaena odorata* (L.) R.M.King & H.Rob.	krus tok' te'	huele de noche	native	shrub	homegarden, wild	no risk	no risk	CBS, NEC, DRS
Asteraceae	*Cirsium* sp.	cholich, tzólich	cardo, cholich	exotic	herb	homegarden, milpa, wild	no risk	no risk	NEC, DRS, CIPD
Asteraceae	*Critonia morifolia* (Mill.) R.M.King & H.Rob.	jólol, pijpejpech, t' unu' holol	cocolmeca, San Isidro	native	shrub	wild	least concern (LC)	no risk	NEC, DGS, DGUS
Asteraceae	*Critonia quadrangularis* (DC.) R.M.King & H.Rob.	holol, jolol	not registered	native	shrub	wild	least concern (LC)	no risk	NEC, DS, DGUS, CIPD, DMSC
Asteraceae	*Erechtites hieracifolia* (L.) Raf.	malil koy	not registered	native	herb	homegarden, milpa, wild	no risk	no risk	CBS, DBBO, NEC
Asteraceae	*Hymenostephium cordatum* S.F.Blake	thapil bichim	not registered	native	herb	homegarden, milpa, wild	no risk	no risk	IPEC
Asteraceae	*Koanophyllon albicaulis* (Sch.Bip. ex Klatt) R.M.King & H.Rob.	tok' te', yaxal	yolochichitl	native	herb	homegarden, wild	no risk	no risk	CBS, DBBO, NEC, DGS, DRS
Asteraceae	*Lagascea helianthifolia* Kunth	polootin	not registered	native	herb	disturbed	no risk	no risk	FIHS
Asteraceae	*Loxothysanus pedunculatus* Rydb.	thak pux	not registered	endemi c	herb	wild	no risk	no risk	CBS, NEC
Asteraceae	*Mikania cordifolia* (L.f.) Willd.	wako	guaco	native	herb	wild	no risk	no risk	CIPD, IPEC
Asteraceae	*Neurolaena lobata* (L.) R.Br. ex Cass.	t' unu' ix bek' em	not registered	native	herb	disturbed	no risk	no risk	PCP
Asteraceae	*Parthenium hysterophorus* L.	ts'a'il kw'eet, tzaile, tzail-cuet	amargoso, chaile	native	herb	wild	no risk	no risk	DS, NEC, DGS, DNS, PCP, CIPD
Asteraceae	*Pluchea odorata* (L.) Cass.	canimim-tzójol, k' animiim ts' ohool	flor de Guadalupe, Santa María	native	herb	homegarden, wild	no risk	no risk	CBS, NEC, DGS, DS, PCP, DRS, DMSC
Asteraceae	*Porophyllum ruderale* subsp. *macrocephalum* (DC.) R.R.Johnson	mithith	hierba del venado	native	herb	homegarden	no risk	no risk	DBBO, NEC, DGS
Asteraceae	*Pseudogynoxys chenopodioides* (Kunth) Cabrera	te' te' wits	not registered	native	herb	disturbed	no risk	no risk	NEC

Asteraceae	*Salmea scandens* (L.) DC.	ix ts'aah, ix-tzaj	chilemecate, hierba del pescado	native	herb	wild	no risk	no risk	DS, DGS, DRS
Asteraceae	*Tagetes erecta* L.	caxiyhuitz, santoorom wits	cempasúchil, flor de muerto	native	herb	homegarden	no risk	no risk	CBS, DS, NEC, PCP, DRS, ENMD, CIPD, DMSC
Asteraceae	*Tagetes lucida* Cav.	ohoom	not registered	native	herb	homegarden	no risk	no risk	CBS
Asteraceae	*Trixis inula* Crantz	pub kw'ahiil	árnica, jarilla	native	shrub	homegarden, milpa, wild	least concern (LC)	no risk	CBS, DS, NEC, PCP, DRS, IPEC
Asteraceae	*Verbesina persicifolia* DC.	huitzín, witsiim	hierba del toro	native	herb	homegarden, milpa, wild	no risk	no risk	CBS, DS, DEMP, NEC, DGS, IPEC
Balsaminaceae	*Impatiens walleriana* Hook.f.	china wits	not registered	exotic	herb	homegarden	no risk	no risk	PCP
Basellaceae	*Anredera vesicaria* (Lam.) C.F.Gaertn.	ix tuyuum	sacacil	native	herb	homegarden, wild	no risk	no risk	CBS, NEC, DS, IPEC, DMSC
Begoniaceae	*Begonia barkeri* Knowles & Westc.	bok'ool uxkwe'	not registered	endemic	herb	wild	no risk	no risk	CIPD
Begoniaceae	*Begonia incarnata* Link & Otto	No registrado	not registered	endemic	herb	wild	no risk	no risk	NEC, DGUS
Begoniaceae	*Begonia wallichiana* Lehm.	hiliy ts'ohool	not registered	endemic	herb	wild	no risk	no risk	NEC, DGS, ENMD
Bignoniaceae	*Amphilophium crucigerum* (L.) L.G.Lohmann	lalab uthu'	lengua de vaca	native	herb	homegarden, wild	no risk	no risk	DS
Bignoniaceae	*Bignonia potosina* (K.Schum. & Loes.) L.G.Lohmann	punath	not registered	native	herb	wild	no risk	no risk	NEC, CIPD
Bignoniaceae	*Crescentia alata* Kunth	thoot tima', tima	coatecomate, tima	native	tree	homegarden, wild	least concern (LC)	no risk	CBS, IPEC
Bignoniaceae	*Dolichandra quadrivalvis* (Jacq.) L.G.Lohmann	oohoox ts' aah, ójox-tzaj	not registered	native	climbing	wild	no risk	no risk	MBND

Bignoniaceae	*Parmentiera aculeata (*Kunth) Seem.	tsoote', tzote	chote	native	tree	homegarden, milpa, wild	least concern (LC)	no risk	DCS, DEMP, NEC, DGS, DS, DGUS, DRS, CIPD, IPEC, DMSC
Bignoniaceae	*Tabebuia rosea* (Bertol.) Bertero ex A.DC.	k'uul	palo de rosa	native	tree	homegarden, wild	least concern (LC)	no risk	CBS, DS, NEC, DGS, NE, IPEC
Bignoniaceae	*Tecoma stans* (L.) Juss. ex Kunth	San Pedro Wits	flor de San Pedro, tronadora	native	shrub	homegarden	least concern (LC)	no risk	ENMD
Boraginaceae	*Cordia alliodora* (Ruiz & Pav.) Oken	álub, huixte, wiixte'	palo de tobacco, palo de viga	native	tree	milpa, sugarcane cultivation, wild	least concern (LC)	no risk	DVS
Boraginaceae	*Ehretia anacua* (Terán & Berland.) I.M.Johnst.	thathup, tihute, tíhute	cacala, coposo, manzanilla, manzanita del monte, raspasombrer o	native	tree	homegarden, milpa, wild	least concern (LC)	no risk	CBS, DS, NEC, DGS, DGUS, FIHS, CIPD, IPEC
Boraginaceae	*Ehretia tinifolia* L.	t' iiw te'	not registered	native	tree	homegarden, wild	no risk	no risk	DGS, CIPD
Boraginaceae	*Heliotropium angiospermum* Murray	thiniy ts' ohool	not registered	native	herb	homegarden, milpa, wild	no risk	no risk	DGS, PCP, IPEC
Boraginaceae	*Heliotropium verdcourtii* Craven	mach' much'	nigua	native	shrub	wild	no risk	no risk	DS, NEC
Boraginaceae	*Tournefortia glabra* L.	waylom te'	not registered	native	tree	wild	least concern (LC)	no risk	CIPD
Brassicaceae	*Cardamine flaccida* Cham. & Schltdl.	t' ik' om ichiich	not registered	native	herb	wild	no risk	no risk	CBS
Brassicaceae	*Lepidium virginicum* L.	tsakam utsun	lentejilla, papayita	native	herb	wild	no risk	no risk	NEC
Bromeliaceae	*Aechmea bracteata* (Sw.) Griseb.	cocomte, cócom-huev, k'ok'om	not registered	native	epiphyte	wild	no risk	no risk	DCS, DVS, NEC, DGS, CIPD

Bromeliaceae	*Ananas comosus* (L.) Merr.	chabcham wits, chabchamhuitz	piña	exotic	rosetophila	homegarden, wild	no risk	no risk	DGS
Bromeliaceae	*Tillandsia schiedeana* Steud.	tsakam k'ok'om	not registered	native	herb	wild	no risk	no risk	NEC
Bromeliaceae	*Tillandsia usneoides* (L.) L.	cúthey, k'uthay	heno, paxtle	native	herb	homegarden, wild	least concern (LC)	no risk	CBS, DS, DGS, FIHS, DRS, CIPD
Burseraceae	*Bursera graveolens* (Kunth) Triana & Planch.	kaxiy tsakah (chaka apestosa)	chaka bruja, chijol prieto, sasafrás	native	tree	homegarden, wild	least concern (LC)	no risk	CBS, NEC, CIPD
Burseraceae	*Bursera simaruba* (L.) Sarg.	tsaka, tzaca	chaca, chaca (á), chaka, palo mulato	native	tree	homegarden, milpa, wild	least concern (LC)	no risk	DS, NEC, DNS, PCP, CIPD, DMSC
Burseraceae	*Protium copal* (Schltdl. & Cham.) Engl.	homte', jom, jomte	copal, copalillo, palo copal	native	tree	wild	least concern (LC)	no risk	CBS, DEMP, NEC, DGS
Cabombaceae	*Cabomba haynesii* Wiersema	weew bexe'	not registered	exotic	herb	wild	no risk	no risk	NEC
Cactaceae	*Acanthocereus tetragonus* (L.) Hummelinck	ocomtzatza, tzatza, xak'ub	jacube, pitaya, pitajaya	native	succulent	homegarden	least concern (LC)	no risk	NEC, DRS
Cactaceae	*Opuntia engelmannii* subsp. *lindheimeri* (Engelm.) U. Guzmán & Mandujano	k'aan k'iith	not registered	native	succulent	homegarden	least concern (LC)	no risk	CBS, DS, NEC, DGS, DRS
Cactaceae	*Opuntia pumila* Rose	bohol pak'ak'	not registered	native	cactus	homegarden	no risk	no risk	NEC, PCP, DRS
Cactaceae	*Pereskia grandiflora* Pfeiff.	pulik kwi'inal	not registered	exotic	cactus	homegarden, wild	no risk	no risk	NEC, DGS, DS, DMSC
Cactaceae	*Pereskiopsis aquosa* (F.A.C.Weber) Britton & Rose	kweteem kwi'inal	not registered	endemic	succulent	homegarden	least concern (LC)	no risk	CBS, NEC, DGS, DRS
Cactaceae	*Rhipsalis baccifera* (J.S.Muell.) Stearn	tzalelte, xi'il Boo'waat	azote	native	succulent	wild	least concern (LC)	no risk	CBS, DCS, DS, NEC, DGS, DGUS,

									MBND, DMSC
Cactaceae	*Selenicereus spinulosus* (DC.) Britton & Rose	bohol tsatsa'	not registered	native	succulent	homegarden	least concern (LC)	no risk	CBS, NEC, DGS, PCP
Cactaceae	*Selenicereus undatus* (Haw.) D.R.Hunt	penxácub, tsatsa', xacub, xácub	jacube	native	climbing	homegarden, wild	data deficient (DD)	no risk	DGS, PCP, CIPD
Cannabaceae	*Celtis iguanaea* (Jacq.) Sarg.	huipuy, thak loh	granjeno	native	climbing	wild	least concern (LC)	no risk	CBS, DS
Cannabaceae	*Trema micrantha* (L.) Blume	puam, puwaamte'	guinda, puan, palo borracho	native	tree	wild	least concern (LC)	no risk	CIPD
Cannaceae	*Canna indica* L.	cuhuap, k' uuwaap, tsak pik' o'	platanillo	exotic	herb	homegarden, milpa, wild	no risk	no risk	DGS, CIPD
Capparaceae	*Cleoserrata serrata* (Jacq.) Iltis	huntal a puunchiix wits	not registered	native	tree	not registered	no risk	no risk	DVS
Capparaceae	*Crateva tapia* L.	tsine te'	palo sordo	native	shrub	wild	least concern (LC)	no risk	DS, DEMP
Caricaceae	*Carica papaya* L.	utsun, útzun	papaya, papaya chiquita, papayo macho (silvestre)	native	tree	homegarden, milpa, wild	data deficient (DD)	no risk	PCP, IPEC
Caryophyllaceae	*Stellaria ovata* Willd. ex Schltdl.	thuyuu'il t'eel	not registered	native	herb	wild	no risk	no risk	DCS, NEC
Celastraceae	*Crossopetalum uragoga* (Jacq.) Kuntze	ts' amuts' uxkwe'	not registered	native	shrub	wild	no risk	no risk	DS, DVS, DGS, DGUS, CIPD
Cleomaceae	*Cleome aculeata* L.	utsun ts' ohool	not registered	native	herb	not registered	no risk	no risk	DGS
Commelinaceae	*Callisia fragrans* (Lindl.) Woodson	pulik utek'	hierba del burro, línea cordial	native	herb	homegarden, wild	no risk	no risk	DCS, DEMP, DNS
Commelinaceae	*Callisia repens* (Jacq.) L.	utek' de bega	not registered	native	herb	homegarden	no risk	no risk	NEC, DGUS

Commelinaceae	*Commelina erecta* L.	utek'	pajilla	native	herb	homegarden, milpa, sugarcane cultivation, wild	least concern (LC)	no risk	DS, NEC, DGS, DGUS, CIPD
Commelinaceae	*Tradescantia zanonia* (L.) Sw.	paktha' utek'	not registered	native	herb	wild	no risk	no risk	DEMP
Commelinaceae	*Tradescantia zebrina* var. *zebrina*	tsak utek'	plateada	exotic	herb	wild	no risk	no risk	CBS, NEC, CIPD
Connaraceae	*Rourea glabra* Kunth	it'iib chuch	chilillo	native	shrub	wild	no risk	no risk	DS, NEC, DMSC
Convolvulaceae	*Cuscuta corymbosa* Ruiz & Pav.	fideo ts' ohool	not registered	native	climbing	wild	no risk	no risk	DS, NEC
Convolvulaceae	*Ipomoea alba* L.	huchuk'	not registered	native	herb	milpa, wild	least concern (LC)	no risk	DS, NEC, DGS, PCP
Convolvulaceae	*Ipomoea batatas* (L.) Lam.	ith, ithi	camote, camote amarillo, camote morado	native	tree	homegarden	data deficient (DD)	no risk	CIPD
Convolvulaceae	*Ipomoea carnea* Jacq.	No registrado	not registered	native	herb	wild	no risk	no risk	PCP
Convolvulaceae	*Ipomoea dumosa* (Benth.) L.O. Williams	thuuyu'	suyo, suyu	native	herb	homegarden, milpa, sugarcane cultivation, wild	no risk	no risk	DVS, PCP, MBND
Convolvulaceae	*Merremia dissecta* (Jacq.) Hallier f.	piith ts'aah	not registered	native	climbing	milpa, wild	no risk	no risk	DBBO, DS
Convolvulaceae	*Merremia umbellata* (L.) Hallier f.	San Diego ts' ohool	not registered	native	climbing	milpa, wild	no risk	no risk	DEMP, NEC
Convolvulaceae	*Operculina pinnatifida* (Kunth) O'Donell	pok' laak	quebra trastes, yedra	native	climbing	wild	no risk	no risk	DS, NEC
Costaceae	*Costus pulverulentus* C.Presl	pakaab olom	caña de puerco	native	herb	homegarden, wild	no risk	no risk	DS, NEC, DGS, DGUS
Crassulaceae	*Kalanchoe pinnata* (Lam.) Pers	papac-tzójol, pocpoc, tolouxéquel, tolow xekel	siempre vida, tronadora	exotic	herb	wild	no risk	no risk	CBS, DCS, DS, NEC, DGS, MBND,

									ENMD, CIPD
Cucurbitaceae	*Cucurbita* sp. 1	ts' oopil uthu'	calabacillo	native	creeping	homegarden, milpa, wild	no risk	no risk	CBS
Cucurbitaceae	*Cucurbita* sp. 2	ts' oop	calabacillo (pared de fruta más gruesa que ts' oopil uthu')	native	creeping	homegarden, milpa, wild	no risk	no risk	CBS
Cucurbitaceae	*Ibervillea* sp.	thokob ts' een	not registered	native	creeping	milpa, wild	no risk	no risk	DS
Cucurbitaceae	*Lagenaria siceraria* (Molina) Standl.	xomom, xómom, kweentu'	guaje	exotic	herb	homegarden	no risk	no risk	DGUS, DRS
Cucurbitaceae	*Melothria pendula* L.	baleeyail an t' eel	not registered	native	creeping	homegarden, milpa, wild	no risk	no risk	NEC
Cupressaceae	*Taxodium mucronatum* Ten.	chuche, chuuche'	sabina, sabino	native	tree	wild	no risk	no risk	PCP, DGUS
Cyperaceae	*Cyperus hermaphroditus* (Jacq.) Standl.	haluk'laab ts' ohool	not registered	native	herb	wild	no risk	no risk	CBS, DS, NEC, DGUS
Cyperaceae	*Eleocharis elegans* (Kunth) Roem. & Schult.	boo' waat toom	tule	native	herb	disturbed	no risk	no risk	CBS
Cyperaceae	*Rhynchospora radicans* (Schltdl. & Cham.) H.Pfeiff.	tathiim toom	not registered	native	herb	wild	no risk	no risk	NEC
Cyperaceae	*Scleria gaertneri* Raddi	t'oyol toom t'unu'	cadillo negro	native	herb	homegarden, wild	least concern (LC)	no risk	DS, DGS, CIPD
Cytinaceae	*Bdallophytum americanum* (R.Br.) Eichler ex Solms	boo'waat wits	not registered	native	herb	wild	no risk	no risk	NEC
Dioscoreaceae	*Dioscorea alata* L.	laab ith	camote real	exotic	herb	homegarden	no risk	no risk	DGUS
Dioscoreaceae	*Dioscorea mexicana* Scheidw.	panil book	not registered	native	herb	homegarden, milpa, sugarcane cultivation	no risk	no risk	PCP, MBND
Dryopteridaceae	*Thelypteris puberula* (Baker) C.V.Morton	tatil mabak	not registered	endemic	herb	wild	no risk	no risk	NEC

Ebenaceae	*Diospyros nigra* (J.F.Gmel.) Perrier	múnec, munek'	chocoyito, muneque, zapote negro	native	tree	homegarden, milpa, wild	no risk	no risk	CBS
Euphorbiaceae	*Acalypha phleoides* Cav.	tsak bohol ts'ohool	not registered	native	herb	not registered	no risk	no risk	DGS
Euphorbiaceae	*Astraea lobata* (L.) Klotzsch	No registrado	not registered	native	herb	milpa, wild	no risk	no risk	DS
Euphorbiaceae	*Bernardia dodecandra* (Sessé ex Cav.) Govaerts	tsinat mahul, tzinatamajuste	not registered	native	tree	wild	no risk	no risk	CBS, DGUS
Euphorbiaceae	*Cnidoscolus multilobus* (Pax) I.M.Johnst.	ac, ak, ak'	mala mujer, ortiga, ortiga blanca	native	tree	milpa, wild	least concern (LC)	no risk	DS, DGS, DGUS, FIHS, IPEC
Euphorbiaceae	*Croton ciliatoglandulifer* Ortega	luc, xoliiman	not registered	native	shrub	homegarden, wild	no risk	no risk	CBS, DS, DMSC
Euphorbiaceae	*Croton cortesianus* Kunth	pushual, puthwal	puhual	native	shrub	milpa, wild	least concern (LC)	no risk	DBBO, DS, DVS, NEC, DGS, DGUS, CIPD, IPEC
Euphorbiaceae	*Croton draco* Schltdl.	xitxte, xixte, xiix te'	chorro de sangre, llorasangre, sangre de grado, sangregrado	native	shrub	milpa, wild	least concern (LC)	no risk	DS, DGS
Euphorbiaceae	*Croton niveus* Jacq.	oli, olíth, thak oliy	algodoncillo, huilote, olí, palo blanco, rama blanca, vara blanca, vidrioso	native	shrub	wild	least concern (LC)	no risk	DS, NEC, DGS
Euphorbiaceae	*Croton reflexifolius* Kunth	oliy	vara blanca	native	shrub	homegarden, milpa, wild	least concern (LC)	no risk	DBBO, DS, DGS
Euphorbiaceae	*Croton soliman* Cham. & Schltdl.	luk	yerba soliiman	native	shrub	homegarden, wild	no risk	no risk	CBS, DGS, DS, DMSC
Euphorbiaceae	*Dalechampia scandens* L.	kw' aat'	not registered	native	climbing	wild	least concern (LC)	no risk	NEC

Euphorbiaceae	*Euphorbia colletioides* Benth.	t' ot' oy ts' ohool	not registered	native	shrub	homegarden	no risk	no risk	DS, NEC
Euphorbiaceae	*Euphorbia dioscoreoides* Boiss.	ehtiil mithith	not registered	endemic	herb	disturbed	no risk	no risk	DS, NEC
Euphorbiaceae	*Euphorbia heterophylla* L.	hab ichiich	nela	native	herb	homegarden, milpa, wild	least concern (LC)	no risk	CBS, DCS, NEC, DGS, PCP, DRS
Euphorbiaceae	*Euphorbia hirta* L.	xa' ts' ohool	not registered	native	herb	disturbed	no risk	no risk	DS
Euphorbiaceae	*Euphorbia hypericifolia* L.	leetsa ts' ohool tsako'	not registered	native	herb	homegarden	no risk	no risk	DS
Euphorbiaceae	*Euphorbia lancifolia* Schltdl.	leetsa ts' ohool	corona de cristo	native	herb	homegarden, wild	no risk	no risk	NEC, DGS, PCP
Euphorbiaceae	*Euphorbia pulcherrima* Willd. ex Klotzsch	oot' wits	noche buena	native	shrub	homegarden	least concern (LC)	no risk	CBS, DGUS, IPEC
Euphorbiaceae	*Euphorbia serpens* Kunth	xaa' uts' aal	not registered	native	herb	homegarden	no risk	no risk	DS, DEMP, DGS, PCP, DRS, CIPD
Euphorbiaceae	*Euphorbia tithymaloides* L.	ácan-tele, akant'ele, ácan-tzácam, akan t'ele', tzójel-túbud, tzójol-tútub	tamaulipa, tamaulipas	native	shrub	homegarden	least concern (LC)	no risk	DS, PCP, IPEC
Euphorbiaceae	*Garcia nutans* Vahl ex Rohr	thocob-otel, thokob ot' el	pimientillo, piñón, piñoncillo	native	shrub	wild	least concern (LC)	no risk	MBND
Euphorbiaceae	*Jatropha curcas* L.	thakpeen te'	piñon	native	shrub	homegarden	least concern (LC)	no risk	DBBO, DS, NEC, DGS
Euphorbiaceae	*Manihot esculenta* Crantz	tinché, t' inche'	yuca	native	shrub	homegarden, milpa, sugarcane cultivation, wild	no risk	no risk	NEC, CIPD

Euphorbiaceae	*Ricinus communis* L.	thikeela', thiquelá	higuera, higuerilla	exotic	shrub	homegarden, wild	no risk	no risk	DS, NEC, DGS, PCP, DRS
Euphorbiaceae	*Tragia mexicana* Müll.Arg.	tiplay'	ortiguilla	native	herb	Wild	no risk	no risk	CBS, DS, CIPD, DMSC
Fabaceae	*Acaciella angustissima* (Mill.) Britton & Rose	xixit	barba de chivo	native	tree	milpa, wild	no risk	no risk	DS, NEC, DGS, CIPD, DMSC
Fabaceae	*Ateleia gummifera* (DC.) D.Dietr.	ta' te'	not registered	native	shrub	wild	endangered (EN)	no risk	DS
Fabaceae	*Bauhinia divaricata* L.	akan pakaax, tatil bichim, tatilbíchim, tzetzemólon	pata de cabra, pata de vaca	native	tree	homegarden, milpa, wild	least concern (LC)	no risk	CBS, DBBO, DS, NEC, DGUS
Fabaceae	*Caesalpinia pulcherrima* (L.) Sw.	San Husee te' (árbol de San José)	not registered	exotic	shrub	homegarden, wild	least concern (LC)	no risk	MBND
Fabaceae	*Calliandra houstoniana* (Mill.) Standl.	huitot, wiit oot'	barba de viejo, cabello de ángel	native	tree	homegarden, milpa, wild	least concern (LC)	no risk	CBS, NEC, DGS, DRS, CIPD, IPEC
Fabaceae	*Canavalia villosa* Benth.	koxol wits	not registered	native	climbing	wild	no risk	no risk	DS
Fabaceae	*Centrosema sagittatum* (Willd.) L.Riley	hik' elom ts' ohool	not registered	native	climbing	homegarden, wild	no risk	no risk	CBS
Fabaceae	*Centrosema virginianum* (L.) Benth.	tsakam koxol wits	not registered	native	climbing	not registered	no risk	no risk	DS
Fabaceae	*Crotalaria vitellina* Ker. Gawl.	thootil tsan	yerba cascabel	native	herb	milpa, wild	no risk	no risk	CBS
Fabaceae	*Dalea scandens* var. *paucifolia* (J.M.Coult.) Barneby	tsakam chilab	yerba azul, yerba de burro	native	shrub	wild	no risk	no risk	DBBO, DEMP, NEC, DGS, IPEC
Fabaceae	*Desmodium affine* Schltdl.	bakaaol t' eel	not registered	native	herb	wild	no risk	no risk	NEC
Fabaceae	*Desmodium axillare* (Sw.) DC.	ts' at' ts' ohool	not registered	native	herb	wild	no risk	no risk	DS
Fabaceae	*Desmodium incanum* DC.	t' apay thekw'eel	yerba de sapo	native	herb	wild	no risk	no risk	DS, NEC, IPEC
Fabaceae	*Desmodium* sp.	xutsun t' eel	not registered	native	herb	not registered	no risk	no risk	IPEC

Fabaceae	*Diphysa americana* (Mill.) M.Sousa	chichath	not registered	native	tree	homegarden, milpa, sugarcane cultivation, wild	no risk	no risk	CBS, DBBO, DS, NEC, PCP, DGUS, CIPD
Fabaceae	*Enterolobium cyclocarpum* (Jacq.) Griseb.	tíyohu, tiyow, tiyuhu	orejón	native	tree	homegarden, wild	no risk	no risk	DRS
Fabaceae	*Erythrina americana* Mill.	hutukuu'	pemoche	endemic	tree	homegarden	no risk	no risk	NEC, DNS, PCP, FIHS
Fabaceae	*Erythrina standleyana* Krukoff	tsamnek hutukuu'	not registered	native	tree	homegarden, wild	least concern (LC)	no risk	CBS, NEC, DGS, PCP, FIHS
Fabaceae	*Eysenhardtia polystachya* (Ortega) Sarg.	chilab	palo azul	native	tree	wild	least concern (LC)	no risk	CBS
Fabaceae	*Guilandina bonduc* L.	eelaa'	haba amarilla, haba de mar	native	shrub	wild	no risk	no risk	NEC, DGS
Fabaceae	*Harpalyce arborescens* A.Gray	cánte, can-té, k' ante', itzcante	brasil, carne de gallina, chicharillo, palo de brasil, quebrache, quebra ache	endemic	tree	milpa, wild	least concern (LC)	no risk	DGS
Fabaceae	*Indigofera suffruticosa* Mill.	manat-yax, tsakam yaax	not registered	native	shrub	wild	no risk	no risk	DBBO, DGS, DNS, MBND
Fabaceae	*Leucaena pulverulenta* (Schltdl.) Benth.	thuc, thuk', tzuqui, xucte, xucté	barba de chivo, palo huax, palo seco	native	tree	milpa, wild	least concern (LC)	no risk	CBS, NEC
Fabaceae	*Lysiloma acapulcense* (Kunth) Benth.	huáyal, wayal	huayatl, rajador colorado, tepehuaje	native	tree	wild	least concern (LC)	no risk	CBS, DS, NEC, DGS, DNS, DGUS, MBND, ENMD
Fabaceae	*Marina scopa* Barneby	tiith olom	not registered	native	shrub	disturbed	no risk	no risk	NEC
Fabaceae	*Mimosa albida* Willd.	chobeem	verguenzosa	native	shrub	wild	least concern (LC)	no risk	IPEC

Fabaceae	*Mimosa pigra* L.	chóben, tsobeem	choveno	native	shrub	milpa, wild	least concern (LC)	no risk	PCP, MBND
Fabaceae	*Mucuna argyrophylla* Standl.	mooxoox	not registered	native	climbing	wild	no risk	no risk	CBS, DS, NEC, CIPD
Fabaceae	*Nissolia fruticosa* Jacq.	bo' xekel	not registered	native	shrub	wild	no risk	no risk	CBS, DS, NEC, IPEC
Fabaceae	*Oxyrhynchus volubilis* Brandegee	wal pooy	bejuco de frijoles	native	climbing	homegarden, wild	least concern (LC)	no risk	DVS
Fabaceae	*Pachyrhizus erosus* (L.) Urb.	cobem, kobeem	jícama	native	climbing	homegarden	no risk	no risk	DS
Fabaceae	*Phaseolus vulgaris* L.	malte' , tsanakw', tzanam, tzanaco	frijol, frijol común, frijol negro, frijol (vaina)	native	herb	homegarden, milpa	least concern (LC)	no risk	DGS
Fabaceae	*Piscidia piscipula* (L.) Sarg.	ts' ihol	chijol	native	tree	homegarden, milpa, wild	no risk	no risk	CBS, DBBO, DS, NEC, FIHS
Fabaceae	*Pithecellobium dulce* (Roxb.) Benth.	jumu, umú, úmuh, umuw	guamúchil, huamúchil, pechejumo, umo,	native	tree	homegarden, wild	least concern (LC)	no risk	NEC, DGS
Fabaceae	*Prosopis laevigata* (Willd.) M.C.Johnst.	ut' u	mezquite	native	tree	wild	least concern (LC)	no risk	CIPD
Fabaceae	*Pseudalbizzia tomentosa* (Micheli) E.J.M.Koenen & Duno	thukiim	not registered	native	tree	wild	least concern (LC)	no risk	CBS, NEC
Fabaceae	*Rhynchosia longeracemosa* M.Martens & Galeotti	tsankw'il t'eel	not registered	native	herb	not registered	no risk	no risk	DS
Fabaceae	*Schrankia* sp.	tsakam tsobeem	not registered	native	herb	homegarden, milpa, wild	no risk	no risk	NEC
Fabaceae	*Senna candolleana* (Vogel) H.S.Irwin & Barneby	cáxey-te, caxi-tzíjol, kaxiy ts' ihol	chijol hediondo, palo	exotic	tree	homegarden, wild	no risk	no risk	CBS, NEC

			hediondo, palo zorrillo						
Fabaceae	*Senna hirsuta* (L.) H.S.Irwin & Barneby	itsaan an bichaam	not registered	native	herb	disturbed	no risk	no risk	NEC, DS, DMSC
Fabaceae	*Senna occidentalis* (L.) Link	bichaam ts' ohool, bícham-tzójol	bichon	native	herb	homegarden	least concern (LC)	no risk	DCS, NEC, PCP, CIPD
Fabaceae	*Senna pendula* (Willd.) H.S.Irwin & Barneby	tsuleek' ekwet	not registered	native	shrub	homegarden	least concern (LC)	no risk	DS, NEC, DGS, DRS
Fabaceae	*Tamarindus indica* L.	tamariindu	tamarindo	exotic	tree	homegarden	least concern (LC)	no risk	CBS, NEC, DNS, CIPD
Fabaceae	*Vachelia cornigera* (L.) Seigler & Ebinger	thobem, thóbem	carne suelo, carnizuelo, cornezuelo	native	tree	milpa, wild	no risk	no risk	DCS, DEMP, DGS, PCP, CIPD, IPEC
Fabaceae	*Vachelia farnesiana* (L.) Wight & Arn.	thuhaanom, thujanom, thujánun	huisache, huizache	native	tree	wild	no risk	no risk	NEC, DGS, DS, DRS, DMSC
Gentianaceae	*Eustoma exaltatum* (L.) Salisb.	bioleta	not registered	native	herb	disturbed	no risk	no risk	NEC
Heliconiaceae	*Heliconia schiedeana* Klotzsch	thúlub, ts' umts' um, tzumtzum	papatla, papatlilla	native	herb	wild	no risk	no risk	DBBO
Iridaceae	*Alophia drummondii* (Graham) R.C.Foster	tsakam apats'	not registered	native	herb	milpa, wild	no risk	no risk	CIPD
Iridaceae	*Eleutherine bulbosa* (Mill.) Urb.	tsakam apats'	not registered	native	herb	homegarden, milpa, wild	no risk	no risk	NEC, CIPD
Lamiaceae	*Callicarpa acuminata* Kunth	elte'	not registered	native	shrub	homegarden, milpa, wild	least concern (LC)	no risk	NEC, DGS, PCP, DGUS
Lamiaceae	*Clerodendrum chinense* (Osbeck) Mabb.	ts' een kwiniimte'	not registered	exotic	shrub	wild	least concern (LC)	no risk	DS
Lamiaceae	*Clinopodium brownei* (Sw.) Kuntze	ts' ots' on ts' ohool	not registered	native	herb	homegarden	no risk	no risk	CBS, NEC, DGS, DS, DMSC

Lamiaceae	*Hedeoma drummondii* Benth.	maal t'eel	polello, poleo	native	herb	homegarden	no risk	no risk	CBS, NEC, DGS, PCP, DRS
Lamiaceae	*Mentha* sp.	elbeenax	menta, yerbabuena, yerba buena	exotic	herb	homegarden	no risk	no risk	NEC, DGS, PCP
Lamiaceae	*Mesosphaerum pectinatum* (L.) Kuntze	tsak maape'	not registered	exotic	herb	milpa, wild	no risk	no risk	DS
Lamiaceae	*Ocimum basilicum* L.	laab thekw'eel	albácar, albahaca, albajaque	native	herb	homegarden	no risk	no risk	NEC, DNS
Lamiaceae	*Ocimum campechianum* Mill.	tsin thekw'eel	albajaca, albajaca del monte	native	herb	homegarden, wild	no risk	no risk	CBS, NEC, DGS, DNS
Lamiaceae	*Ocimum carnosum* (Spreng.) Link & Otto ex Benth.	ik' ts'ohool	not registered	native	herb	wild	no risk	no risk	NEC
Lamiaceae	*Origanum* sp.	mehorana	mejorana	exotic	climbing	homegarden	no risk	no risk	CBS
Lamiaceae	*Salvia coccinea* Buc'hoz ex Etl.	hut'ut' wits	mirto	native	herb	homegarden, milpa, sugarcane cultivation, wild	no risk	no risk	CBS, DVS, NEC, DNS, DGUS, CIPD
Lamiaceae	*Scutellaria seleriana* Loes.	elbeenax ts'ohool	not registered	native	herb	wild	no risk	no risk	CBS, NEC, DGS, DS, DMSC
Lamiaceae	*Teucrium cubense* Jacq.	tihtsan kw'eet	apazotillo, epazotillo, gallina ciega, hierba del negro,hierb a de la gallina, ipazotillo	native	herb	homegarden, milpa, wild	no risk	no risk	DCS, DBBO, DS, NEC, DGS
Lauraceae	*Persea americana* Mill.	oh, oj, uh, uj	aguacate, aguacate chico, aguacate criollo, aguacate oloroso	native	tree	homegarden, wild	least concern (LC)	no risk	DS, DRS

Loasaceae	*Mentzelia hispida* Willd.	thekw'em ch' ohool, thekw'em ts'ojol	not registered	native	herb	not registered	no risk	no risk	DS, MBND
Loranthaceae	*Psittacanthus schiedeanus* (Schltdl. & Cham.) G.Don	ok' lom te' puulik	not registered	native	epiphyte	wild	no risk	no risk	DS, NEC, DGS, PCP, IPEC
Loranthaceae	*Struthanthus crassipes* (Oliv.) Eichler	ok' lom te'	not registered	native	shrub	not registered	no risk	no risk	DGS
Loranthaceae	*Struthanthus quercicola* (Schltdl. & Cham.) D.Don	ok' lom te'	secapalo	native	shrub	homegarden	no risk	no risk	DCS, NEC
Lygodiaceae	*Lygodium venustum* Sw.	cútil-papá, k'util papaam	not registered	native	herb	milpa, sugarcane cultivation, wild	no risk	no risk	NEC, DGS, PCP, MBND, ENMD, CIPD
Lythraceae	*Cuphea decandra* Dryand.	arroz ts' ohool	not registered	native	shrub	wild	no risk	no risk	DS
Lythraceae	*Cuphea salicifolia* Schltdl. & Cham.	No registrado	not registered	endemic	shrub	wild	no risk	no risk	NEC
Lythraceae	*Heimia salicifolia* (Kunth) Link	maan witsiil	jarilla	native	shrub	not registered	no risk	no risk	CBS, NEC
Lythraceae	*Lythrum gracile* Benth.	lehem ts' ohool	jarilla	native	shrub	wild	no risk	no risk	CIPD
Lythraceae	*Punica granatum* L.	laab bek	granada	exotic	tree	homegarden	least concern (LC)	no risk	NEC
Malpighiaceae	*Malpighia glabra* L.	k' ak' al ilaal	capulín	native	shrub	homegarden, wild	least concern (LC)	no risk	NEC, DRS
Malvaceae	*Abelmoschus moschatus* Medik.	kwinim ilaal	café castilla, café de castila	exotic	herb	homegarden	no risk	no risk	DCS, NEC, DGS, DS, DRS, DMSC
Malvaceae	*Abutilon hypoleucum* A.Gray	hupchil, tzacotzójol, tzacotxójol	vara blanca	native	herb	disturbed	no risk	no risk	NEC, DGS
Malvaceae	*Allosidastrum pyramidatum* (Desp. ex Cav.) Krapov., Fryxell & Bates	t' unu' ix bek' em, thipom, thipon, thípon	malva, malvita	native	shrub	wild	no risk	no risk	PCP

Malvaceae	*Anoda cristata* (L.) Schltdl.	bakan ts'ohool	violeta	native	herb	homegarden, milpa, wild	no risk	no risk	DS, NEC, ENMD, CIPD
Malvaceae	*Ceiba pentandra* (L.) Gaertn.	unup, únup	ceiba	native	tree	wild	least concern (LC)	no risk	DS, NEC
Malvaceae	*Corchorus siliquosus* L.	pehtsul kw' eet, thípon	not registered	native	shrub	homegarden, milpa, wild	no risk	no risk	CBS, DS, DVS, NEC, DGS, PCP, DGUS, IPEC
Malvaceae	*Gossypium hirsutum* L.	cuinim, thak kwinim, tsokoy	algodón	endemic	shrub	homegarden	vulnerable (VU)	no risk	DCS, NEC, DGS, DS, PCP, DGUS, DRS, CIPD, DMSC
Malvaceae	*Guazuma ulmifolia* Lam.	akich, áquich	aquiche, guásima, guazima, guázima	native	tree	homegarden, milpa, wild	least concern (LC)	no risk	DS, NEC, DGS, PCP, DRS, CIPD, IPEC
Malvaceae	*Heliocarpus donnellsmithii* Rose	bat, baat, jolol	jonote, jonote baboso, malva	native	tree	homegarden, milpa, wild	no risk	no risk	CBS, DCS, NEC, DGS, PCP, DRS, CIPD, IPEC
Malvaceae	*Herissantia crispa* (L.) Brizicky	No registrado	not registered	native	herb	wild	no risk	no risk	DGUS
Malvaceae	*Hibiscus lavateroides* Moric.	thoot wits	not registered	native	herb	homegarden	no risk	no risk	PCP, DGUS
Malvaceae	*Hibiscus rosa-sinensis* L.	tulipaan	tulipaan, tulipán	exotic	herb	homegarden	no risk	no risk	PCP
Malvaceae	*Malachra capitata* (L.) L.	pulik thipon	not registered	native	herb	wild	no risk	no risk	NEC
Malvaceae	*Malvastrum americanum* (L.) Torr.	thipon	not registered	native	herb	milpa, wild	no risk	no risk	DS, NEC, DGS, IPEC
Malvaceae	*Malvastrum coromandelianum* (L.) Garcke	lantha thipon	not registered	native	herb	milpa, wild	no risk	no risk	DS, CIPD
Malvaceae	*Malvaviscus arboreus* Dill. ex Cav.	ix bek' em	aguate	native	shrub	homegarden, wild	least concern (LC)	no risk	DCS, NEC, DGS, DS, PCP, DGUS, DRS, DMSC
Malvaceae	*Melochia nodiflora* Sw.	tsakam akich	not registered	native	shrub	wild	no risk	no risk	NEC, PCP, DGUS, CIPD

Malvaceae	*Melochia pyramidata* L.	ehtiil i tsakam akich	not registered	native	shrub	disturbed	least concern (LC)	no risk	NEC
Malvaceae	*Pavonia schiedeana* Steud.	ts'ikiy uxum	not registered	native	herb	wild	no risk	no risk	CBS, DS, NEC, DGS, PCP, DGUS, DRS, CIPD, DMSC
Malvaceae	*Phymosia umbellata* (Cav.) Kearney	tsak kwiniimte'	not registered	native	herb	wild	least concern (LC)	no risk	CIPD
Malvaceae	*Pseudobombax ellipticum* (Kunth) Dugand	mococ, mócoc, mokok	coquito, huauchilol, mocoque	native	tree	homegarden, wild	least concern (LC)	no risk	CBS, NEC, DGS, DNS, DGUS, MBND, CIPD
Malvaceae	*Sida acuta* Burm.f.	thak thipon	not registered	native	shrub	homegarden, milpa, wild	no risk	no risk	CBS, DS, DGS, IPEC
Malvaceae	*Triumfetta semitriloba* Jacq.	thipaxi', thipaxi, thípaxi	cardillo, hierba de la hormiga	native	shrub	homegarden, milpa, sugarcane cultivation, wild	least concern (LC)	no risk	NEC, DGS, PCP, DGUS, DRS, CIPD
Marantaceae	*Maranta arundinacea* L.	t' aaw'	hoja de sahagu, sagu, papatlitla	native	herb	homegarden	no risk	no risk	CBS, NEC, DGS, DGUS, CIPD
Marantaceae	*Maranta gibba* Sm.	t' aaw' ok	not registered	native	herb	homegarden, wild	no risk	no risk	NEC, CIPD
Martyniaceae	*Martynia annua* L.	itsik' kuxkum, ítsik' kuxkum	not registered	native	herb	homegarden	no risk	no risk	DRS, CIPD
Melanthiaceae	*Schoenocaulon officinale* (Schltdl. & Cham.) A.Gray	sebadiya	cebadilla	native	herb	milpa, wild	no risk	no risk	DS
Melastomataceae	*Miconia petiolaris* (Schltdl.) Michelang.	tsakam chikab ts' ohool	not registered	native	shrub	wild	no risk	no risk	DCS, NEC
Meliaceae	*Cedrela odorata* L.	icte, ik' te'	cedro, cedro colorado	native	tree	homegarden, wild	vulnerable (VU)	subject to special protection (PR)	CBS, DBBO, NEC, DRS, CIPD, IPEC

Meliaceae	*Trichilia havanensis* Jacq.	colobte, cololte, cólol-te, thokob saantu	ciruelillo, estrebilla, estribillo, naranjillo, ¿naranjillo?, palo de cuchara	native	tree	homegarden, milpa, wild	least concern (LC)	no risk	CBS, DBBO, NEC, DRS
Menispermaceae	*Cissampelos pareira* L.	huálec-tzójol, k' on k' ach	hierba de la víbora	native	climbing	homegarden, milpa, sugarcane cultivation, wild	no risk	no risk	CBS, NEC
Menispermaceae	*Cocculus diversifolius* DC.	lek' ab t' iim	brillosa	native	climbing	homegarden, milpa, wild	no risk	no risk	CBS, DCS, NEC, DGS
Montiaceae	*Talinum fruticosum* (L.) Juss.	thak akw'aal	quelite	native	herb	homegarden	no risk	no risk	DGS, DGUS, CIPD
Montiaceae	*Talinum paniculatum* (Jacq.) Gaertn.	pitsits wal paktha'	quelite	native	herb	homegarden	no risk	no risk	CBS, DGS
Moraceae	*Brosimum alicastrum* Sw.	ohox, ojox, ojx	ojite, ramón	native	tree	homegarden, milpa, wild	least concern (LC)	no risk	DGS
Moraceae	*Castilla elastica* Cerv.	pem, peem	hule, palo de hule	native	tree	homegarden, wild	least concern (LC)	no risk	NEC, DGS, DS, DRS, IPEC, DMSC
Moraceae	*Dorstenia contrajerva* L.	k' ubak kw' a'	contra hierba	native	herb	wild	no risk	no risk	CBS, NEC, DGS, DS, DGUS, DMSC
Moraceae	*Ficus americana* Aubl.	ts' uh	higeron chico	native	tree	homegarden, wild	least concern (LC)	no risk	DBBO, NEC, DS, IPEC, DMSC
Moraceae	*Ficus maxima* Mill.	hopoy	higeron grande, nacapule	native	tree	wild	least concern (LC)	no risk	NEC, DGS, IPEC
Moraceae	*Ficus obtusifolia* Kunth	hopoy ts' uh, jópoy, tejópoy, tzuj	jalamate, jopoy, higo, higuerón,	native	tree	wild	least concern (LC)	no risk	NEC, DGS, DS, IPEC, DMSC

			laurel, palo de higo						
Moraceae	*Ficus pertusa* L.f.	tsakam ts' uh	not registered	native	tree	homegarden, wild	least concern (LC)	no risk	DS, NEC, DRS, CIPD, IPEC, DMSC
Moraceae	*Maclura tinctoria* (L.) D.Don ex Steud.	tsitsil, tsitsiy, tzitzi, tzitziy, tzitzil	mora, mora amarilla	native	tree	wild	least concern (LC)	no risk	CBS, DS, NEC, DGS, DS, DGUS, FIHS, MBND, DRS, DMSC
Moraceae	*Trophis racemosa* (L.) Urb.	tsumte', tzumte	chumte, mora, ramoncillo, zacate ramón	native	tree	milpa, wild	least concern (LC)	no risk	CBS, DGS
Muntingiaceae	*Muntingia calabura* L.	puam, tsakam puwaamte'	guinda, puan	native	tree	disturbed	no risk	no risk	DGUS
Musaceae	*Musa paradisiaca* L.	it' ath, ítath	plátano, plátano chaparro	exotic	tree	homegarden, sugarcane cultivation	no risk	no risk	DS, CIPD
Myrtaceae	*Eugenia capuli* (Schltdl. & Cham.) Hook. & Arn.	chuk ba' im, pehte', piste	capulin, capulín, capulincillo	native	tree	milpa, wild	least concern (LC)	no risk	NEC, DGS, DRS
Myrtaceae	*Eugenia karwinskyana* O.Berg	akan koox	not registered	endemic	tree	wild	least concern (LC)	no risk	CBS
Myrtaceae	*Eugenia oerstediana* O.Berg	ha' pehte'	not registered	native	shrub	homegarden, wild	least concern (LC)	no risk	DGS
Myrtaceae	*Psidium guajava* L.	bec, bek	guayaba, guayabo, guayabillo	native	tree	homegarden, wild	least concern (LC)	no risk	NEC, DGS, DGUS, DRS, CIPD
Myrtaceae	*Psidium guineense* Sw.	bekil tooro	guayaba de burro	native	tree	wild	least concern (LC)	no risk	DGS
Nyctaginaceae	*Bougainvillea spectabilis* Willd.	buganbiya	bugambilia, buganvilla	exotic	shrub	homegarden	no risk	no risk	DBBO

Nyctaginaceae	*Mirabilis jalapa* L.	ch' uyeem	not registered	native	herb	homegarden, wild	no risk	no risk	CBS, NEC, PCP, DRS, CIPD
Nyctaginaceae	*Neea psychotrioides* Donn.Sm.	k' ak' al xeklek	not registered	native	shrub	wild	least concern (LC)	no risk	NEC, CIPD
Nyctaginaceae	*Pisonia aculeata* L.	loh, loj	caltute, granjeno, granjeno prieto, tumtum prieto, uña de gato	native	shrub	wild	least concern (LC)	no risk	DS, NEC
Nymphaeaceae	*Nymphaea ampla* (Salisb.) DC.	pulul	not registered	native	herb	wild	no risk	no risk	NEC, PCP
Oleaceae	*Jasminum* sp.	hasmiin	jasmin	exotic	tree	homegarden	no risk	no risk	NEC, DRS
Onagraceae	*Oenothera kunthiana* (Spach) Munz	waakal mo'eel	not registered	native	herb	homegarden, wild	no risk	no risk	NEC, DGS
Onagraceae	*Oenothera rosea* L'Hér. ex Aiton	ts'een waakal mo'eel	yerba de golpe	native	herb	wild	no risk	no risk	IPEC
Onagraceae	*Oenothera tetraptera* Cav.	waakal mo'eel	yerba de golpe, yerba de colico	native	herb	homegarden	no risk	no risk	DCS, NEC, DGS, DNS
Orchidaceae	*Epidendrum difforme* Jacq.	tsooy pathum	not registered	native	herb	wild	no risk	no risk	DCS, NEC
Orchidaceae	*Isochilus linearis* (Jacq.) R.Br.	toom	not registered	native	herb	homegarden, wild	no risk	no risk	CIPD
Orchidaceae	*Kionophyton seminuda* (Schltr.) Garay	kw'itool ts'ohool	not registered	endemic	herb	wild	no risk	no risk	PCP
Orchidaceae	*Trichocentrum carthagenense* (Jacq.) M.W.Chase & N.H.Williams	xutsun buuru	not registered	native	herb	wild	no risk	no risk	NEC
Oxalidaceae	*Oxalis dillenii* Jacq.	kital ichiich	not registered	native	herb	not registered	no risk	no risk	CBS
Oxalidaceae	*Oxalis latifolia* Kunth	hilil ts'ohool	not registered	native	herb	homegarden, milpa, wild	no risk	no risk	DS, DGS
Pandanaceae	*Pandanus odorifer* (Forssk.) Kuntze	bathuch	cardoncillo, guapilla	exotic	rosetophila	homegarden, wild	least concern (LC)	no risk	DS, IPEC, DMSC

Papaveraceae	*Argemone mexicana* L.	tsolich, tzólich	chicalote	native	herb	disturbed	no risk	no risk	DVS
Papaveraceae	*Bocconia frutescens* L.	ts'ix te'	mano de león, palo santo	native	shrub	wild	least concern (LC)	no risk	DGS
Passifloraceae	*Passiflora coriacea* Juss.	ocobithut, okoob thut'	murciélago	native	herb	wild	no risk	no risk	DS, DEMP, NEC, CIPD
Passifloraceae	*Passiflora foetida* L.	pok' pok'	maracuyá	native	herb	homegarden, milpa, wild	no risk	no risk	NEC, DGS, PCP, DGUS, CIPD
Petiveriaceae	*Petiveria alliacea* L.	pathaam, pátham	hierba del zorrillo, pegarropa	native	herb	homegarden, wild	no risk	no risk	CBS, NEC, DGS, IPEC
Petiveriaceae	*Rivina humilis* L.	itzil-cua, taa' t'ele'	baja tripa, hierba de la víbora	native	herb	homegarden, wild	no risk	no risk	CBS, DS, NEC, CIPD, IPEC
Phyllanthaceae	*Phyllanthus adenodiscus* Müll.Arg.	pok' thoot	cascabel	endemic	shrub	homegarden, milpa, wild	no risk	no risk	DS, NEC, CIPD
Phyllanthaceae	*Phyllanthus niruri* L.	kux ichiich	tamarindillo	native	herb	homegarden, wild	no risk	no risk	CBS, DS, NEC, MBND
Picramniaceae	*Picramnia antidesma* Sw.	thal te'	not registered	native	tree	wild	no risk	no risk	DS
Pinaceae	*Pinus teocote* Schied. ex Schltdl. & Cham.	pithomlaab	not registered	native	tree	wild	least concern (LC)	no risk	CBS, NEC, DGS
Piperaceae	*Peperomia glabella* (Sw.) A.Dietr.	tsakam ix tuyuum	not registered	native	herb	wild	no risk	no risk	IPEC
Piperaceae	*Peperomia liebmannii* C. DC.	wiyab ts'ohool	not registered	native	herb	wild	no risk	no risk	CBS
Piperaceae	*Peperomia rotundifolia* (L.) Kunth	boton ts'ohool, (Alcorn)	hierba botón	native	herb	wild	no risk	no risk	NEC
Piperaceae	*Peperomia* sp.	homte' ts'ohool	hierba del árbol de copal	native	herb	wild	no risk	no risk	CBS, NEC
Piperaceae	*Piper amalago* L.	kw' alal its' aamal, yaxal, yaxil	cordoncillo, rodilla de venado	native	tree	homegarden, wild	least concern (LC)	no risk	CBS, DS, NEC, PCP, DRS, DMSC

Piperaceae	*Piper auritum* Kunth	lácap-uxcue, tiiya'	acuyo, rama de queso, tepacántaro	native	tree	homegarden, wild	least concern (LC)	no risk	NEC, DGS
Piperaceae	*Piper schiedeanum* Steud.	paktha yexal	not registered	native	tree	wild	least concern (LC)	no risk	CIPD
Piperaceae	*Piper umbellatum* L.	bakaaol a iits'	not registered	native	tree	wild	no risk	no risk	DS, NEC, DGS, PCP, DRS, CIPD, IPEC, DMSC
Plantaginaceae	*Mecardonia procumbens* (Mill.) Small	tsakam wiichab	not registered	native	herb	homegarden	no risk	no risk	CBS, NEC, MBND, ENMD, CIPD
Plantaginaceae	*Russelia equisetiformis* Schltdl. & Cham.	kutsiilte'	not registered	native	shrub	homegarden	no risk	no risk	CBS, NEC
Plantaginaceae	*Russelia sarmentosa* Jacq.	kwayab ts'aale	not registered	native	shrub	milpa, wild	no risk	no risk	CBS, DGUS
Plumbaginaceae	*Plumbago zeylanica* L.	hurika	eurika, jurica	native	herb	not registered	no risk	no risk	DCS, IPEC
Poaceae	*Arundinella berteroniana* (Schult.) Hitchc. & Chase	itse' toom	not registered	native	herb	wild	no risk	no risk	CBS
Poaceae	*Arundo donax* L.	pacab, pakaab	carrizo	exotic	tree	sugarcane cultivation, wild	least concern (LC)	no risk	CBS, DS, CIPD, DMSC
Poaceae	*Cenchrus echinatus* L.	t' oyol k' iith	cadillo	native	herb	wild	least concern (LC)	no risk	CBS, NEC, DGS, DGUS, CIPD
Poaceae	*Cenchrus preslii* (Kunth) ined.	pakaab	carrizo	native	herb	sugarcane cultivation, wild	no risk	no risk	CBS, NEC, CIPD
Poaceae	*Cymbopogon citratus* (DC.) Stapf	limoon toom	zacate limón	exotic	herb	homegarden	no risk	no risk	NEC, DGS, DGUS
Poaceae	*Hilaria ciliata* (Scribn.) Sohns	No registrado	not registered	endemic	herb	wild	no risk	no risk	DGS
Poaceae	*Imperata brasiliensis* Trin.	ataa toom	zacate de casa	native	herb	homegarden, wild	no risk	no risk	DGS, DGUS, CIPD

Poaceae	*Lasiacis ruscifolia* (Kunth) Hitchc. ex Chase	pacab, tseey kw'a'	carrizillo	native	herb	wild	no risk	no risk	NEC, DGS, DS, DGUS, DMSC
Poaceae	*Lithachne pauciflora* (Sw.) P.Beauv.	tsakam pakaab	not registered	native	herb	wild	no risk	no risk	NEC, PCP
Poaceae	*Panicum virgatum* L.	itse' toom	not registered	native	herb	wild	no risk	no risk	DRS
Poaceae	*Paspalum paniculatum* L.	huchum toom	not registered	native	herb	Wild	least concern (LC)	no risk	NEC
Poaceae	*Paspalum* sp.	belal toom	not registered	native	herb	not registered	no risk	no risk	DS
Poaceae	*Saccharum officinarum* L.	pacab, pakab, uhuatl	caña, caña de azúcar	exotic	herb	homegarden, sugarcane cultivation	no risk	no risk	CIPD
Poaceae	*Setaria liebmannii* E.Fourn.	weew ok	cola de zorra	native	herb	disturbed	no risk	no risk	DGS
Poaceae	*Zea mays* L.	em, eem	maíz	native	herb	homegarden, milpa	no risk	no risk	CBS, DS, NEC, DGS, DGUS, DRS, ENMD
Polygalaceae	*Polygala nitida* var. *lithophila* (S.F. Blake) T. Wendt	tsakam tsak mokok	not registered	native	herb	disturbed	no risk	no risk	DS, DGUS, FIHS
Polygalaceae	*Securidaca diversifolia* (L.) S.F.Blake	mamaal tsan	not registered	native	herb	homegarden, milpa, wild	least concern (LC)	no risk	DS, DGS, DMSC
Polygonaceae	*Polygonum mexicanum* Small	ehtiil tok'oy	not registered	native	herb	wild	no risk	no risk	NEC
Polypodiaceae	*Campyloneurum phyllitidis* (L.) C. Presl	wa' ub ik'	not registered	native	epiphyte	wild	no risk	endangere d (A)	CBS, DCS, DEMP, NEC
Polypodiaceae	*Microgramma nitida* (J. Sm.) A.R. Sm.	chu'ub te	not registered	native	herb	homegarden, wild	no risk	no risk	DCS, DS, IPEC, DMSC
Polypodiaceae	*Pecluma plumula* (Humb. & Bonpl. ex Willd.) M.G. Price	ehtiil weew koxol	not registered	native	herb	wild	no risk	no risk	NEC
Polypodiaceae	*Phlebodium aureum* (L.) J. Sm.	cuath, ts'een k'ubak koy	calahuala, canahuala, rejalgar	native	herb	wild	no risk	no risk	NEC, PCP, DRS
Polypodiaceae	*Phlebodium decumanum* (Willd.) J. Sm.	k'ubak koy	canahuala	native	herb	milpa, sugarcane	no risk	no risk	CBS, NEC, DGS, PCP,

						cultivation, wild			DRS, CIPD, IPEC
Polypodiaceae	*Pleopeltis polypodioides* (L.) E.G.Andrews & Windham	koo' te'	not registered	native	herb	homegarden, wild	no risk	no risk	CBS, NEC
Portulacaceae	*Portulaca oleracea* L.	pitsits wal, pitzitzhual	ni (verdolaga), verdolaga	native	herb	homegarden	least concern (LC)	no risk	NEC, DGS
Portulacaceae	*Portulaca pilosa* L.	tsakam tsatsa', tsakam tsatsa'ts'ojol	amor de un rato	native	herb	homegarden	no risk	no risk	DS, NEC, DRS, CIPD
Primulaceae	*Ardisia escallonioides* Schltdl. & Cham.	hualpúchun, pejte, pelat puchun	capulín, capulín agrio, manzanita	native	shrub	wild	least concern (LC)	no risk	NEC, DGS, CIPD
Primulaceae	*Ardisia nigrescens* Oerst.	k'ak'al ilaal paktha'	not registered	native	shrub	wild	least concern (LC)	no risk	NEC
Primulaceae	*Parathesis serrulata* (Sw.) Mez	apulee'	capulín, capulín arribeño	native	tree	wild	no risk	no risk	NEC, DGS
Pteridaceae	*Adiantum poiretii* Wikstr.	acampich, ácampich, akan pich	culantrillo, helecho	native	herb	homegarden, wild	no risk	no risk	CBS, DCS, NEC
Pteridaceae	*Adiantum tenerum* Sw.	akan pich	not registered	native	herb	wild	no risk	no risk	NEC
Pteridaceae	*Adiantum tricholepis* Fée	akan pich	not registered	native	herb	Wild	no risk	no risk	NEC
Pteridaceae	*Hemionitis radiata* (L.) Christenh.	kaxam	not registered	native	herb	wild	no risk	no risk	CBS
Ranunculaceae	*Clematis grossa* Benth.	ithim wahuts	barba de chivo	native	climbing	homegarden, wild	no risk	no risk	DS, DGS, DRS
Rhamnaceae	*Colubrina greggii* S.Watson	alts' een puwaamte', ehtiil tsakam akich	vara prieta	native	shrub	wild	least concern (LC)	no risk	DS, DRS, DMSC
Rhamnaceae	*Gouania polygama* (Jacq.) Urb.	thuhal ts' aah	not registered	native	climbing	wild	no risk	no risk	CBS, DCS, DS
Rhamnaceae	*Karwinskia humboldtiana* (Schult.) Zucc.	itsil, itzil, ítzil	capulín, capulín de corona, capulincillo,	native	tree	homegarden, wild	no risk	no risk	CBS

			carabuyo, chalchanote, negrito, tullidor						
Rosaceae	*Prunus samydoides* Schltdl.	tsak te'	not registered	endemic	tree	wild	least concern (LC)	no risk	NEC, DGS, CIPD
Rosaceae	*Rosa* sp.	gloorya wits	rosa de castilla	exotic	shrub	homegarden	no risk	no risk	NEC, CIPD
Rubiaceae	*Chiococca alba* (L.) Hitchc.	puut' ts' aah	not registered	native	shrub	wild	least concern (LC)	no risk	DBBO, NEC, DS, DMSC
Rubiaceae	*Coffea arabica* L.	capé, kapee	café	exotic	shrub	homegarden, wild	endangered (EN)	no risk	IPEC
Rubiaceae	*Hamelia patens* Jacq.	chac-loc, tsak look', tzacloc	chacloc (o), hierba del toro, madura-plátano, vara prieta, zapote maduro	native	shrub	homegarden, milpa, wild	least concern (LC)	no risk	DBBO, DS, NEC, DGS, DGUS, CIPD, IPEC
Rubiaceae	*Psychotria erythrocarpa* Schltdl.	baina Ts' ohool	not registered	native	shrub	wild	no risk	no risk	DS, NEC, DGS, CIPD
Rubiaceae	*Psychotria nervosa* Sw.	tse' tsem ts 'ohool	not registered	native	shrub	homegarden, wild	least concern (LC)	no risk	CBS, DS, NEC, DRS, CIPD
Rubiaceae	*Psychotria* sp.	thuyum olom	not registered	native	shrub	wild	no risk	no risk	DS
Rubiaceae	*Randia laetevirens* Standl.	tsotsoob olom	not registered	native	shrub	wild	least concern (LC)	no risk	NEC, DGS, IPEC
Rutaceae	*Citrus aurantiifolia* (Christm.) Swingle	hiliy lemoon	limón	exotic	tree	homegarden, wild	no risk	no risk	CBS, DS, NEC, DGS, PCP, DRS, CIPD
Rutaceae	*Citrus limon* (L.) Osbeck	chuuchu' liima	not registered	exotic	tree	homegarden, wild	no risk	no risk	DRS
Rutaceae	*Citrus* x *aurantium* L.	jily-lanax, jiliy lánax,	naranja agria, naranjo agrio	exotic	tree	homegarden, wild	no risk	no risk	CBS, DCS, DS, NEC, DGS, DNS,

		thimaloon lanaax							MBND, DRS, CIPD
Rutaceae	*Decatropis bicolor* (Zucc.) Radlk.	bichaam te', tzatzubtzá	cigarillo, rosa amarilla, Santo Domingo	endemic	herb	homegarden, wild	no risk	no risk	CBS, NEC, DGS
Rutaceae	*Esenbeckia berlandieri* Baill.	lanaax te'	hueso de tigre	native	tree	wild	endangered (EN)	no risk	NEC
Rutaceae	*Zanthoxylum fagara* (L.) Sarg.	huipuy, Wi'puuy	limoncillo, naranjillo, uña de gato	native	tree	homegarden, wild	least concern (LC)	no risk	NEC, DGS, DS, PCP, CIPD, DMSC
Salicaceae	*Casearia aculeata* Jacq.	chumak chul	not registered	native	tree	wild	no risk	no risk	DS, DRS
Salicaceae	*Casearia laetioides* (A.Rich.) Warb.	atamte, bolantin, thácamte	volador, volantín	native	tree	milpa, wild	no risk	no risk	DVS, NEC
Salicaceae	*Populus mexicana* Sarg.	itsow	álamo	endemic	tree	wild	near threatened (NT)	no risk	NEC, MBND
Salicaceae	*Salix humboldtiana* Willd.	tocoy, tócoy, tok'oy	sauce, sauz	native	tree	wild	least concern (LC)	no risk	NEC
Salicaceae	*Xylosma flexuosa* (Kunth) Hemsl.	tsak k'iith	espina de la corona, granedilla	native	shrub	homegarden, milpa, wild	least concern (LC)	no risk	DGS, DS, DRS, CIPD, IPEC, DMSC
Santalaceae	*Phoradendron quadrangulare* (Kunth) Griseb.	ok' lom te' yexu', óclomte	huevo de iguana, injerto, oclomte, secapalo	native	tree	homegarden, wild	no risk	no risk	CBS, NEC
Sapindaceae	*Exothea copalillo* (Schltdl.) Radlk.	t' il homte'	not registered	endemic	tree	wild	endangered (EN)	no risk	NEC, DNS, MBND
Sapindaceae	*Paullinia tomentosa* Jacq.	t'in kamab	not registered	native	tree	homegarden, milpa, wild	no risk	no risk	DS, NEC, DGS, DGUS, FIHS, CIPD

Sapindaceae	*Sapindus saponaria* L.	huálul, walul	jaboncillo	native	shrub	homegarden, milpa, wild	least concern (LC)	no risk	CBS, NEC
Sapindaceae	*Serjania rachiptera* Radlk.	tsank' ub ts'ohool	not registered	native	herb	milpa, sugarcane cultivation, wild	no risk	no risk	DGS, CIPD
Sapindaceae	*Urvillea ulmacea* Kunth	tsaayleelaab ts'ohool	not registered	native	herb	homegarden, wild	no risk	no risk	NEC
Sapotaceae	*Manilkara zapota* (L.) P.Royen	bólom, bólom-itath, bolom it'ath, tsab it' ath, tzabitatb, tzabitath	chico sapote, chicozapote, chico zapote, mamey	native	tree	homegarden, wild	least concern (LC)	no risk	CIPD
Scrophulariaceae	*Buddleja americana* L.	jacte, pulik elte'	jacte, tepozán, tepusano	native	shrub	homegarden	least concern (LC)	no risk	NEC, CIPD, IPEC
Scrophulariaceae	*Capraria mexicana* Moric. ex Benth.	pulik ts' itsiimbe ts' ohool	yerba del caballo	native	shrub	wild	no risk	no risk	DGS, DNS
Scrophulariaceae	*Capraria saxifragifolia* Schltdl. & Cham.	akan t' ot	not registered	endemi c	herb	wild	no risk	no risk	DCS, DBBO
Selaginellaceae	*Selaginella martensii* Spring	chuklaab ts'ohool	yoxon	native	herb	wild	no risk	no risk	CBS, NEC, MBND, IPEC
Selaginellaceae	*Selaginella* sp.	mul ichiich	not registered	native	herb	homegarden	no risk	no risk	NEC
Smilacaceae	*Smilax domingensis* Willd.	weew uut'	not registered	native	climbing	milpa, wild	no risk	no risk	NEC, DGS, DGUS, CIPD, IPEC
Smilacaceae	*Smilax* sp.	zaarza	sarsa, zarza	native	tree	homegarden, wild	no risk	no risk	DGUS
Solanaceae	*Capsicum annuum* L.	itz, kamab uut', tzacam-itz	chile, chile pico de pájaro, chile piquín, chile piquín chico, chile piquín grande, chile	native	herb	homegarden, milpa, sugarcane cultivation, wild	least concern (LC)	no risk	CIPD

			verde, chiltepín, quipín						
Solanaceae	*Capsicum annuum* var. *glabriusculum* (Dunal) Heiser & Pickersgill	kulum its, tsakam its	chili piquín	native	herb	homegarden, wild	no risk	no risk	CBS, DS, IPEC
Solanaceae	*Cestrum dumetorum* Schltdl.	tsabalte', tzabal-té	alcajuda, arcajuda, horcajuda, orcajuda	native	shrub	homegarden, milpa, wild	no risk	no risk	CBS, DS, NEC, DGS, DNS, PCP, DGUS, DRS, CIPD, IPEC, DMSC
Solanaceae	*Cestrum nocturnum* L.	ehek tsabalte'	orcajuda prieta	native	shrub	milpa, wild	least concern (LC)	no risk	CBS, CIPD
Solanaceae	*Brugmansia* x *candida* Pers.	kampaana wits	floripundio	exotic	shrub	homegarden, wild	no risk	no risk	CBS, DS, MBND, IPEC
Solanaceae	*Datura stramonium* L.	thanab	toloache	native	herb	homegarden	no risk	no risk	CBS, DS, NEC, DGUS, MBND, CIPD
Solanaceae	*Nicotiana tabacum* L.	may, maay	tabaco	native	herb	homegarden	no risk	no risk	DCS, NEC, DGS, IPEC
Solanaceae	*Physalis gracilis* Miers	tuthaayil an t' ot	not registered	native	herb	homegarden, milpa, wild	least concern (LC)	no risk	CIPD
Solanaceae	*Physalis melanocystis* (B.L.Rob.) Bitter	akal k' ak' al ilaal	not registered	native	shrub	homegarden, wild	least concern (LC)	no risk	DEMP, NEC
Solanaceae	*Physalis solanaceus* (Schltdl.) Axelius	tuthaayil an t' iiw	not registered	native	herb	milpa, wild	no risk	no risk	NEC, CIPD
Solanaceae	*Physalis virginiana* Mill.	tuthaayil an t' ot	tomatillo del monte	native	herb	homegarden, milpa, wild	least concern (LC)	no risk	DS
Solanaceae	*Physalis viscosa* L.	tuthaayil an t' ot	tomatillo del monte	native	herb	homegarden, milpa, wild	no risk	no risk	NEC, DGS, CIPD

Solanaceae	*Solandra maxima* (Moc. & Sessé ex Dunal) P.S.Green	tima' wits	bolsa de judas	endemic	tree	homegarden	no risk	no risk	DVS, NEC
Solanaceae	*Solanum americanum* Mill.	ichamal, wal ts'ok	hierbamora, yerba mora	native	herb	homegarden, wild	no risk	no risk	DS, DEMP, CIPD, IPEC
Solanaceae	*Solanum aphyodendron* S.Knapp	t' unu' tsabalte'	not registered	native	shrub	homegarden, wild	no risk	no risk	DS, NEC
Solanaceae	*Solanum diphyllum* L.	tsakam tsabalte'	orcajudo verde	native	tree	homegarden, milpa, wild	no risk	no risk	CBS, DS, DEMP, NEC, DRS
Solanaceae	*Solanum lanceifolium* Jacq.	itsik' mitsu'	not registered	native	tree	wild	no risk	no risk	PCP
Solanaceae	*Solanum torvum* Sw.	muuthuuts'	sosa espinosa	native	tree	homegarden, milpa, wild	no risk	no risk	DS, NEC, DGS, MBND, IPEC
Solanaceae	*Solanum umbellatum* Mill.	maayte'	sauco de zorrillo, sausa	native	tree	homegarden, milpa, wild	no risk	no risk	CBS, NEC, DS, DMSC
Tectariaceae	*Tectaria heracleifolia* (Willd.) Underw.	weew koxol	lengua de cierba	native	herb	wild	no risk	no risk	CBS, DCS, DBBO, NEC, DGS, PCP, CIPD
Urticaceae	*Cecropia obtusifolia* Bertol.	tsulte', tzulte	jarsilla, palo de violín, trompeta, trompetillo	native	tree	wild	least concern (LC)	no risk	DS
Urticaceae	*Pilea imparifolia* Wedd.	tsakam tsahib	not registered	native	herb	wild	no risk	no risk	NEC
Urticaceae	*Pilea microphylla* (L.) Liebm.	ha' il tsan	not registered	native	herb	wild	no risk	no risk	CBS, DS, NEC, DGS, CIPD
Urticaceae	*Pilea pubescens* Liebm.	pux lat' em	chichicastle	native	herb	wild	no risk	no risk	CBS, DVS, NEC, PCP, DRS, CIPD
Urticaceae	*Pouzolzia occidentalis* (Liebm.) Wedd.	uxum ilaal	not registered	native	herb	homegarden	no risk	no risk	CBS, DCS, NEC, DS, PCP, DGUS, DRS, IPEC, DMSC

Urticaceae	*Urera caracasana* (Jacq.) Gaudich. ex Griseb.	aamlay', cocotze, cocotzte, púxlatem, tzac	chaucaquilla (malhombre)	native	tree	wild	least concern (LC)	no risk	DVS, CIPD
Urticaceae	*Urera* sp.	tsooklay'	not registered	native	shrub	homegarden	no risk	no risk	DCS, NEC, DS, DRS, CIPD, DMSC
Urticaceae	*Urtica chamaedryoides* Pursh	la'ix iits'	not registered	native	herb	wild	no risk	no risk	DS, DMSC
Verbenaceae	*Citharexylum berlandieri* S.Watson	wal to' ol te'	not registered	native	shrub	wild	least concern (LC)	no risk	DS, DMSC
Verbenaceae	*Verbena delticola* Small ex Perry	waleklaab ts' ohool	not registered	native	herb	homegarden, sugarcane cultivation, wild	no risk	no risk	CBS, DS, NEC, PCP
Verbenaceae	*Lantana achyranthifolia* Desf.	thak patelax	frutilla	native	shrub	milpa, wild	no risk	no risk	NEC, DGS
Verbenaceae	*Lantana camara* L.	patelaxhuitz, tsak patelax	chancaquilla	native	shrub	homegarden, milpa, wild	no risk	no risk	CBS, DS, DEMP, NEC, DGS, DGUS, CIPD, IPEC
Verbenaceae	*Lantana involucrata* L.	baron	orozúz, peonia	native	shrub	wild	least concern (LC)	no risk	NEC
Verbenaceae	*Lippia myriocephala* Schltdl. & Cham.	anaamte', ánamte, anamté, ananté	corazón amarillo	native	tree	homegarden, milpa, wild	least concern (LC)	no risk	DS, NEC, DGS, DGUS, CIPD
Verbenaceae	*Lippia origanoides* Kunth	ananté, (Alcorn)	orégano	native	shrub	homegarden	no risk	no risk	DGS
Verbenaceae	*Petrea volubilis* L.	tháthub, thathup ts'aah	bejuco de caballo, cualmecate, raspasombrer o	native	shrub	homegarden, milpa, wild	no risk	no risk	NEC, DGS, PCP, DGUS, MBND, DRS, CIPD
Verbenaceae	*Priva lappulacea* (L.) Pers.	t'apay ts' ohool	not registered	native	herb	homegarden, milpa, wild	no risk	no risk	DGS
Verbenaceae	*Tamonea curassavica* (L.) Pers.	ook' t' ithith	not registered	native	herb	milpa, wild	no risk	no risk	DGS, DGUS

Vitaceae	*Cissus microcarpa* Vahl	kux tsamnek	not registered	native	climbing	wild	no risk	no risk	CIPD
Vitaceae	*Cissus verticillata* (L.) Nicolson & C.E.Jarvis	yax tsamnek	not registered	native	climbing	milpa, wild	least concern (LC)	no risk	DS, IPEC
Zamiaceae	*Ceratozamia mexicana* Brongn.	konlib, tzalam-thipac	not registered	native	herb	homegarden, wild	critically endangere d (CR)	endangere d (A)	DGUS
Zamiaceae	*Zamia* sp.	tsalaam thipaak	not registered	native	shrub	wild	no risk	no risk	CBS, NEC, DGUS, ENMD, CIPD, IPEC
Zingiberaceae	*Curcuma longa* L.	azafraan	azafraan, azafrán, camotito	exotic	herb	homegarden	data deficient (DD)	no risk	DS, DRS, DMSC
Zingiberaceae	*Zingiber officinale* Roscoe	labitz, laab its	ajenjible, jengibre	exotic	herb	homegarden	data deficient (DD)	no risk	NEC, DGS, DS, DRS, DMSC
